# Multiomics Approach Reveals an Important Role of BNIP3 in Myocardial Remodeling and the Pathogenesis of Heart Failure with Reduced Ejection Fraction

**DOI:** 10.3390/cells11091572

**Published:** 2022-05-06

**Authors:** Antoine H. Chaanine, LeeAnn Higgins, Lothar Lauterboeck, Todd Markowski, Qinglin Yang, Patrice Delafontaine

**Affiliations:** 1 Department of Medicine, Tulane University, New Orleans, LA 70112, USA; pdelafon@tulane.edu; 2 Department of Physiology, Tulane University, New Orleans, LA 70112, USA; 3 Department of Biochemistry, Molecular Biology, and Biophysics, University of Minnesota Medical School, Minneapolis, MN 55455, USA; higgi022@umn.edu (L.H.); marko025@umn.edu (T.M.); 4 Department of Pharmacology, Louisiana State University Health Sciences Center, New Orleans, LA 70112, USA; lothar.lauterboeck@thermofisher.com (L.L.); qyang1@lsuhsc.edu (Q.Y.); 5 Department of Pharmacology, Tulane University, New Orleans, LA 70112, USA

**Keywords:** heart failure, myocardial remodeling, metabolic remodeling, calcium cycling, BNIP3, PKA signaling, apoptosis

## Abstract

Previous work showed a role of BNIP3 in myocardial remodeling and progression to HFrEF. We utilized a multiomics approach to unravel BNIP3-related molecular mechanisms in the pathogenesis of HFrEF. BNIP3 knockdown in HFrEF improved glycolysis, pyruvate metabolism, branched-chain amino acid catabolism, and oxidative phosphorylation, and restored endoplasmic reticulum (ER)–mitochondrial (mt) calcium and ion homeostasis. These effects of BNIP3 on cardiac metabolism were related to its interaction and downregulation, and/or phosphorylation, of specific mt-proteins involved in the aforementioned metabolic pathways, including the MICOS and SLC25A families of carrier proteins. BNIP3 affected ER–mt-calcium and ion homeostasis via its interaction-induced VDAC1 dimerization and modulation of VDAC1 phosphorylation at Ser104 and Ser241, and the downregulation of LETM1. At the ER level, BNIP3 interacted with the enzyme SERCA2a and the PKA signaling complex, leading to the downregulation of SERCA2a and PKA-mediated Ser16 phospholamban phosphorylation. Additionally, BNIP3 attenuated AMPK and PRKCE activity by modulating AMPK phosphorylation at Ser485/491 and Ser377 residues, and PRKCE phosphorylation at Thr521 and Thr710 residues. BNIP3 also interacted with sarcomeric, cytoskeletal, and cellular transcription and translation proteins, and affected their expression and/or phosphorylation. In conclusion, BNIP3 modulates multiple pathobiological processes and constitutes an attractive therapeutic target in HFrEF.

## 1. Introduction

Heart failure with reduced ejection fraction (HFrEF) is associated with eccentric myocardial remodeling, increases in LV volumes, and a depressed LV ejection fraction (LVEF), secondary to complex structural and molecular changes at the cellular level [1]. These changes take place after an insult to the myocardium, such as myocardial infarction and/or prolonged hypertension, or as a consequence of drug-induced myocardial toxicity or inherited mutations that affect sarcomeric or cytoskeletal proteins [1]. Consequently, the altered workload due to biomechanical and neurohumoral stress activates complex, maladaptive cellular signaling that drives the initiation of myocardial remodeling and the progression towards HFrEF [2]. This is due to derangements in calcium cycling and ion homeostasis, as well as metabolic remodeling [3] and extracellular matrix remodeling [4], leading to enhanced apoptosis and myocardial stiffness, respectively.

The Bcl-2 nineteen kilodalton interacting protein 3 (BNIP3) has been shown to play a role in mitophagy and apoptosis [5,6,7,8]. BNIP3 expression was upregulated under conditions of cardiac stress [9] and in human HFrEF [10]. The intensity of its expression correlated with mitochondrial (mt) fragmentation, increases in LV volumes, and a decrease in LVEF [10]. The targeted ablation of BNIP3 in a murine myocardial infarction model restrained postinfarction remodeling by attenuating apoptosis in the peri-infarct and remote regions [11]. Moreover, BNIP3 knockdown in a rat pressure overload HFrEF model reversed myocardial remodeling, enhanced contractility, and restored endoplasmic reticulum (ER)–mt-calcium homeostasis [12]. The molecular mechanisms related to BNIP3-mediated myocardial remodeling have not been fully elucidated. In this study, we utilized a multiomics approach to globally assess and unravel mechanistic insights into the role of BNIP3 in promoting myocardial remodeling and dysfunction.

## 2. Materials and Methods

### 2.1. Production of Recombinant Adenoviruses and Adeno-Associated Virus

The production of adenoviruses (Ad) encoding for Null (control), BNIP3, or BNIP3 shRNA (ShBNIP3) was performed by Vector Biolabs (Malverin, PA, USA), as previously described [13]. The production of adeno-associated virus serotype 9 (AAV9) encoding for luciferin ShRNA (ShLuc) or ShBNIP3 was performed as previously described [12,14].

### 2.2. Isolation and Culture of Adult Rat Cardiomyocytes and Design of In Vitro Experiments

The isolation of rat adult cardiac myocytes (ACM) was performed in male Sprague Dawley rats weighing 250–350 g, as previously described [13]. All reagents for ACM isolation were purchased from Sigma-Aldrich (St. Louis, MO, USA). The procedure was approved by the Animal Care and Use Committee of Tulane University, and adhered to the Guide for the Care and Use of laboratory Animals published by the National Institutes of Health. Following heart digestion, ACM separation, and calcium reintroduction, ACM were plated on laminin-coated culture dishes in full nutrient M199 medium, and were allowed to attach for 1 h in humidified 5% CO_2_, 95% air at 37 °C. Subsequently, they were washed once with M199 medium to remove unattached and dead cells. The ACM were then transfected with Ad-Null, Ad-BNIP3, or Ad-ShBNIP3, with an infectious dose of 100 multiplicity of infection, for 36 h to allow for sufficient gene transduction before the following experiments were performed.

#### 2.2.1. Assessment of Mt-Reactive Oxygen Species (ROS) and Mt-Membrane Potential

The ACM were loaded with the red mt-superoxide indicator (5 μmol/L MitoSOX—Thermo Fisher Scientific, Waltham, MA, USA) and tetra-methylrhodamine methyl ester perchlorate (50 nmol/L TMRM—Thermo Fisher Scientific, Waltham, MA, USA) for 10 min at 37 °C to assess mt-ROS and mt-membrane potential, respectively. Cells were then washed with M199 medium and baseline live images were taken at 20× magnification using an Olympus IX71 fluorescent microscope (Olympus, Tokyo, Japan). Subsequently, cells were stressed with phenylephrine (10 μmol/L PE—Sigma-Aldrich, St. Louis, MO, USA) and imaged at 2 h and 12 h following PE stress. Photomicrographs were taken from at least 5 fields per condition per group. Fluorescence intensity was analyzed in ImageJ. Data were obtained from at least 3 independent experiments.

#### 2.2.2. Measurement of Mt-Oxygen (O_2_) Flux (J_O2_) and Oxidation Phosphorylation (OXPHOS)

Mitochondrial respiration was assessed and analyzed with an Oroboros oxygraph-2k, using an appropriate respiration buffer (MiR05—Innsbruck, Austria) at high sample dilutions. The titration method of substrates, inhibitors, and uncouplers was utilized for the measurement of state 2 and 3 of respiration and respiratory capacity at multiple levels of the respiratory chain, as previously described [15,16]. Briefly, ACM were washed with PBS, then scraped and permeabilized with Saponin (final concentration 30 μg in 1 mL MiR05) for 5 min. The cells were then centrifuged at 700 rpm for 20 s, and then resuspended in MiR05 medium. The cells were subsequently counted, and 2 × 10^4^ rod-shaped ACM were added to each chamber of the Oroboros instrument. Glutamate (10 mmol/L) and malate (2 mmol/L) were added to produce NADH and to activate the ETC complex I (state 2-LEAK phase). ADP (2.5 mmol/L) was then added to maximally stimulate respiration, in the presence of glutamate and malate, to assess the OXPHOS phase (state 3). Next, succinate (5 mmol/L) was added to produce FADH2, which, together with glutamate and malate, allow for the assessment of complex I and II activity combined, and, therefore, maximal OXPHOS capacity. Afterwards, Rotenone (1 μmol/L) was added to inhibit complex I and measure complex II activity only. Oligomycin (2 μmol/L) was then added to inhibit ATP synthase activity (state 4 respiration), followed by the addition of carbonyl cyanide-p-trifluoromethoxyphenyl-hydrazone (2 μmol/L FCCP) as an uncoupler to assess electron transfer system (ETS) capacity. Thereafter, Antimycin A (5 μmol/L) was added to inhibit complex III activity for the determination of residual O_2_ consumption. Finally complex IV activity was measured by adding N,N,N′,N′-tetramethyl-p-phenylenediamine dihydrochloride (0.5 mmol/L TMPD). All reagents were purchased from Sigma-Aldrich (St. Louis, MO, USA). Data were obtained from 7 independent experiments.

### 2.3. Animal Model of Ascending Aortic Banding and Gene Delivery

All procedures involving the handling of animals were approved by the Animal Care and Use Committee of Tulane University, and adhered with the Guide for the Care and Use of laboratory Animals published by the National Institutes of Health. Sprague Dawley rats weighing 180–200 g underwent ascending aortic banding (AAB), as previously described [17]. The effects of AAB in rats were followed using echocardiography to identify changes in LV structure and function, as previously described [12]. Systolic HF (HFrEF) developed at 8 weeks post-AAB, and the animals were randomized to receive 5E11 DNase-resistant particles of AAV9-ShLuc vs. AAV9-ShBNIP3 via tail vein injection. The rats were then followed for 1 month until an endpoint was reached. Sham-operated animals underwent the same procedure, although without the creation of AAB, and were subjected to saline tail vein injection. Echocardiographic data for all groups are presented in Supplementary Table S1.

### 2.4. Discovery-Based Proteomic and Phosphoproteomic Quantitative Analysis Using Isobaric Labeling and Liquid Chromatography–Mass Spectrometry

Protein extraction from frozen LV and right ventricular (RV) myocardia was performed as previously described [18]. Following protein extraction, a 210 μg aliquot of each sample was transferred to a new 1.5 mL microfuge tube and brought to the same volume with protein extraction buffer plus 8 mM chloroacetamide. All samples were diluted 5-fold with water, and then trypsin (Promega, Madison, WI, USA) was added in a 1:40 ratio of trypsin to total protein for in-solution proteolytic digestion, as previously described [18]. Subsequently, a 200 μg aliquot of each sample was made, and peptides in each sample were labeled with the corresponding TMT10plex™ isobaric label reagent, 0.8 mg (Thermo Scientific, Watham, MA, USA) per the manufacturer’s protocol, as previously described [18]. After labeling, all the samples within the same TMT™ experiment were multiplexed together into a new 1.5 mL microfuge tube. To look at the global proteome and phosphorylated proteome, the pooled TMT™ sample was split 10:90 global-to-phospho, respectively, to provide an aliquot for global TMT™ protein quantification and an aliquot for phosphopeptide TMT™ quantification. Phosphopeptides were enriched with the High-Select™ TiO2 phosphopeptide kit and the High-Select™ Fe-NTA phosphopeptide kit using the High-Select™ SMOAC (sequential enrichment of metal oxide affinity chromatography) protocol, per the manufacturer’s instructions (Thermo Fisher Scientific, Rockford, IL, USA). The TiO2 and Fe-NTA samples were reconstituted and run separately on a Thermo Orbitrap Fusion™ Tribrid™ mass spectrometer (Thermo Fisher Scientific, Waltham, MA, USA). Global TMT sample fractionation, Orbitrap Fusion liquid chromatography–mass spectrometry (LC–MS) peptide and phosphopeptide analysis, and database searching was performed as previously described [18].

Protein and phosphoprotein identification was performed in Proteome Discoverer (Thermo Fisher Scientific, Waltham, MA, USA), as previously described [18]. We used Scaffold Q+ (version 4.9, Proteome Software Inc., Portland, OR, USA) for validation of MS-based peptide and protein identification, and for quantification, as described previously [18]. Briefly, channels were corrected according to the algorithm described in i-Tracker [19] in all samples, and normalization was performed on intensities across samples and spectra, as described previously [20]. Median values were used for averaging. Spectra data were then log_2_-transformed, stripped of those matched to multiple proteins, and weighted by an adaptive intensity weighting algorithm. Differentially expressed proteins across experimental groups were determined by applying a permutation test with Benjamini–Hochberg correction for multiple comparisons [21]. An unadjusted *p*-value of <0.05 was considered significant. Phosphopeptide quantification was performed in Scaffold PTM (version 3.2, Proteome Software Inc., Portland, OR, USA), as previously described [18], the results of which were normalized with the global protein quantification results from the related TMT experiment using protein XML exports from Scaffold Q+. Further details on phosphopeptide quantification in Scaffold PTM are described elsewhere [18].

The normalized, raw, log_2_-transformed, MS data were exported from Scaffold or Scaffold PTM, and were then uploaded to Qlucore bioinformatics software for the visualization and presentation of the analyzed proteomic or phosphoproteomic data in Scaffold or Scaffold PTM, respectively. Data visualization was accomplished via the generation of heat maps, PCA plots, and Venn diagrams. Bioinformatics analysis of the aforementioned datasets was performed in Ingenuity Pathway Analysis (IPA) bioinformatics software by utilizing IPA’s “Core Analysis” function with a cutoff *p*-value of <0.05 for each of the two-group comparisons: ShLuc vs. Sham and ShBNIP3 vs. ShLuc. Analyzed datasets were then compared using IPA’s “Comparison Analyses” function to yield the most enriched canonical pathways and upstream regulators that were shared between the two-group comparisons. Protein–protein interaction (PPI) networks and enriched reactome pathways, by q-value, of the commonly identified mt-proteins in both, LV and RV, proteomic datasets that were differentially downregulated in ShLuc vs. Sham and differentially upregulated in ShBNIP3 vs. ShLuc, was performed in the STRING database. A q-value of < 0.05 was considered significant.

IPA analysis content information for the LV and RV proteomic datasets were the following: Analysis Creation Date: 17 September 2021; Content version: 65367011 (Release Date: 4 June 2021; Analysis IDs: 38809479 and 38809526, respectively. IPA analysis content information for the LV and RV p-proteomic datasets were the following: Analysis Creation Date: 7 September 2021 and 26 September 2021, respectively; Content version: 65367011 (Release Date: 4 June 2021) and 68752261 (Release Date: 6 September 2021), respectively; Analysis IDs: 38776621 and 38833098, respectively.

### 2.5. Discovery-Based Non-Targeted Central Carbon Metabolism and Acyl-Carnitine Analysis via LC–MS

Sample preparation for the non-targeted metabolomics and acyl-carnitine LC–MS analysis was performed as described elsewhere [18]. A total of 50 ± 2 mg tissue was used for both experiments. Metabolites were identified by matching the retention time and mass (+/− 10 ppm) to authentic standards. For the non-targeted metabolomics analysis, peak areas were integrated using Profinder v8.00 (Agilent Technologies, Santa Clara, CA, USA). Data were normalized to urine creatinine levels, and Loess drift correction was applied using the area of each metabolite in the control samples (pools) for correction, as previously described using MetaboDrift 1.0 [22]. Non-targeted data analysis was performed using Agilent’s MassHunter Find by Molecular Feature workflow (v7.0) with recursion using Agilent’s Mass Profiler Pro (v8.0.). The data were processed using Binner [23], as previously described [18]. Duplicate identified metabolites that had the same mass and retention time, and similar abundance among the studied biological samples, were removed. For the acyl-carnitine analysis, isotope peak areas were integrated using MassHunter Quantitative Analysis vB.07.00 (Agilent Technologies, Santa Clara, CA, USA) of metabolite fluxes for acyl-carnitine.

MS raw data were uploaded to Qlucore bioinformatics software, and then log_2_-transformed. Statistical analysis was performed in Qlucore using one-way ANOVA and multiple comparison correction by Tukey. A *p*-value of <0.05 was considered statistically significant. Data visualization (heat maps, volcano plots, PCA plots, and Venn diagrams) of the statistically analyzed dataset was also performed in Qlucore. Bioinformatics analysis of the non-targeted metabolomics datasets was performed by utilizing IPA’s “Core Analysis” function with a cutoff *p*-value of <0.05 for each of the two-group comparisons: ShLuc vs. Sham and ShBNIP3 vs. ShLuc. Analyzed datasets were compared using IPA’s “Comparison Analyses” function, as described above. Enrichment pathway analysis was performed in MetaboAnalyst v5.0 bioinformatics software.

IPA analysis content information for the metabolomics dataset analyzed in Qlucore were the following: Analysis Creation Date: 28 September 2021; Content version: 68752261 (Release Date: 6 September 2021); Analysis ID: 38841804.

### 2.6. Co-Immunoprecipitation and MS-Based Protein Identification

The co-immunoprecipitation (Co-IP) experiment was performed as previously described [24]. Briefly, protein lysates were prepared from rat LV myocardia of Sham (n = 3) and pressure overload (PO)-induced HFrEF (n = 3) groups, as well as from a tissue bank of human HFrEF LV myocardia (n = 3) obtained from patients at the time of LVAD implantation. Consent was obtained from the patients prior to collecting tissue, and their clinical characteristics were published in our previous work [10]. A 500 μg aliquot from each sample was incubated with 10 μg mouse monoclonal BNIP3 primary antibody (Abcam, Cambridge, MA, USA; Cat# ab10433). The Co-IP eluate from each sample was boiled for 15 min, and then ran on SDS-PAGE 12% separating gel, followed by trypsin (Promega, Madison, WI, USA) in-gel digestion and protein identification via the nano-liquid chromatography (LC)–MS/MS platform (Ultimate 3000 nano UHPLC system—Thermo Fisher Scientific, Waltham, MA, USA).

Mass Spectrometry: The full scan was performed on a Q Exactive Orbitrap mass spectrometer between 300–1650 *m*/*z* at a resolution of 60,000 at 200 *m*/*z*. The automatic gain control target for the full scan was set to 3e6. The MS/MS scan was operated in top 20 mode using the following settings: resolution of 15,000 at 200 *m*/*z*; automatic gain control target 1e5; maximum injection time 19 ms; normalized collision energy at 28%; isolation window of 1.4 Th; charge state exclusion unassigned, 1, >6; dynamic exclusion 30 s. The MS raw files were analyzed and searched against rat or human protein databases using Maxquant (1.6.2.6). The parameters were set as follows: the protein modifications were carbamidomethylation (C) (fixed), oxidation (M) (variable), phospho (STY) (variable); the enzyme specificity was set to trypsin; the precursor ion mass tolerance was set to 10 ppm; MS/MS tolerance was 0.5 Da. Label-free quantification was used to quantify protein expression among the different samples. MS raw data were then uploaded and log_2_-transformed in Qlucore for data visualization. Differentially expressed proteins across experimental rat HFrEF and Sham groups was determined by applying Student’s *t*-test with a cutoff *p*-value of <0.05 vs. <0.1. Bioinformatic analysis of the statistically analyzed rat BNIP3 interactome dataset was performed by utilizing IPA’s “Core Analysis” function with a cutoff *p*-value of <0.05 vs. <0.1. The two analyses were then compared using IPA’s “Comparison Analyses” function, as described above. The PPI networks and enriched reactome pathways, by q-value, of the identified BNIP3 interacting proteins in rat or human LV myocardium, and those that were commonly identified in both, was performed in the STRING database. A q-value of < 0.05 was considered significant.

IPA analysis content information for the rat BNIP3 interactome, HFrEF vs. Sham, were the following: Analysis Creation Date: 19 October 2021; Content version: 68752261 (Release Date: 6 September 2021); Analysis ID: 38908519 (HFrEF vs. Sham, *p* < 0.05) and 38908520 (HFrEF vs. Sham, *p* < 0.1).

### 2.7. Validation by Western Blot

Western blot was performed as previously described [12]. The following primary antibodies were used: total OXPHOS rodent antibody cocktail, HADHA, HADHB, MCAD, VDAC1, SLC25A4, SLC25A5, SLC25A11 (Abcam, Boston, MA, USA); BNIP3 rodent specific, GAPDH, AMPKα, p-S485/491-AMPKα, p-T197-AMPKα, GSK3α, p-S21-GSK3α, GSK3β, p-S9-GSK3β, TNNI3, p-S23/24-TNNI3, and PP1a (Cell signaling, Danvers, MA, USA); PGC-1α (Novus Biologicals, Centennial, CO, USA); LETM1 (Proteintech, Rosemont, IL, USA); SERCA2A, PLN, and p-S16-PLN (Badrilla, Leeds, UK). The p-S79-BNIP3, p-S104-VDAC1, and p-S241-VDAC1 antibodies were custom-designed by Thermo Fisher (Waltham, MA, USA; 1:1000 dilution). All antibody dilutions were performed as per the manufacturer’s manual for each of the purchased antibodies.

### 2.8. Validation by Co-IP and Western Blot

The Co-IP and western blot experiment was performed as previously described [12] using the Dynabeads Protein G IP kit as per the manufacturer’s protocol (Thermo Fisher Waltham, MA, USA). Protein lysates were prepared from normal rat ACM (n = 3), rat LV myocardia of Sham (n = 3), and PO-induced HFrEF (n = 3) groups, as well as from the tissue biobank of human HFrEF LV myocardia (n=3), as described above. A 250 μg aliquot from each sample was incubated with 5 μg mouse monoclonal BNIP3 primary antibody (Abcam, Cambridge, MA, USA; Cat# ab10433). The Co-IP eluate from each sample was boiled for 5 min, and then ran on SDS-PAGE gel. Thereafter, the experiment was completed as previously described [12]. The following primary antibodies were used for the western blot part of the experiment: SERCA2A (Badrilla, Leeds, UK); BNIP3 rodent specific and BNIP3 human specific (Cell signaling, Danvers, MA, USA); LETM1 (Proteintech, Rosemont, IL, USA).

### 2.9. Statistical Analysis

The statistical methods used for the analysis of the discovery-based proteomics, phosphoproteomics, and untargeted metabolomics, as well as for the rat BNIP3 interactome, HFrEF vs. Sham, were described in detail in the above corresponding sections. The western blot analysis data, MitoSOX and TMRM fluorescence intensities, and mt-O_2_ flux and OXPHOS analysis data were presented as mean ± standard deviation. Statistical analyses were performed in Prism software version 9.1.0 using one-way ANOVA with Benjamini correction method. A *p*-value < 0.05 was considered significant.

## 3. Results

### 3.1. Echocardiographic Phenotyping of the Studied Animals in Proteomic Studies

Echocardiographic parameters of the studied animals in Sham, ShLuc, and ShBNIP3 groups are presented in Supplementary Table S1 and Supplementary Figure S1A–D. At week (wk)3 post-AAB, there was evidence of concentric hypertrophy, with over 1.5-fold increases in both LV septal and posterior wall thicknesses in both (ShLuc and ShBNIP3) HFrEF groups vs. Sham. The LV end-diastolic (LVEDV) and end-systolic (LVESV) volumes were decreased and LVEF was increased in both HFrEF groups vs. Sham. At wk8 post-AAB, the time point of systolic heart failure development and gene delivery, both groups retained the same degree of concentric hypertrophy vs. Sham, but developed significant increases in LVEDV and LVESV, and a decrease in LVEF vs. Sham and wk3 post-AAB (Supplementary Table S1 and Supplementary Figure S1A–D). At wk4 post-Rx (wk12 post-AAB), the LV volumes (LVEDV and LVESV) and LVEF increased and decreased further, respectively, in the ShLuc group vs. Sham and wk8 post-AAB; meanwhile, the LV volumes and LVEF decreased and increased, respectively, in ShBNIP3 vs. ShLuc and wk8 post-AAB (Supplementary Table S1 and Supplementary Figure S1B–D). The heart weight, LV weight, and RV weight to body weight (BW) ratios increased in both HFrEF groups vs. Sham (Supplementary Table S1). The RV weight to BW ratio decreased in ShBNIP3 vs. ShLuc (Supplementary Table S1).

### 3.2. Visualization of the LV and RV Proteomic Datasets

Heat maps and PCA plots of the total identified proteins in LV and RV TMT-labeled shotgun proteomic datasets are presented in Supplementary Figure S2A,B. The PCA plots show clustering of biological samples within each group. They also show that the highest variance was between the Sham and HFrEF groups biological-sample-based LV > RV proteomic datasets. Moreover, there was variance between the ShBNIP3 and ShLuc groups, with the ShBNIP3 biological samples being slightly closer to Sham than ShLuc. This suggests an overall improvement in the ShBNIP3-treated group, which was more pronounced in the RV than the LV myocardium. The majority of proteins and mt-proteins that were identified in the LV proteomic dataset were also identified in the RV proteomic dataset, as shown in the Venn diagrams in Supplementary Figure S2C. This enabled the direct comparison between both (LV and RV) proteomic datasets. The differentially expressed proteins in ShLuc vs. Sham and ShBNIP3 vs. ShLuc in the LV and RV proteomic datasets are shown in Supplementary Excel file S1. Visualization of the differentially expressed proteins and mt-proteins in ShLuc vs. Sham is shown in Supplementary Figures S3A–F and S4B,C. The heat maps and PCA plots in Supplementary Figures S3A,B and S4B,C show the differential log_2_-fold change and the variance in biological samples in Sham, ShLuc, and ShBNIP3 groups, for the differentially expressed proteins and mt-proteins in ShLuc vs. Sham, respectively. Similar variance can be seen between the three-group biological samples, as discussed above, but the degree of variance was more pronounced between Sham and the HFrEF groups in both proteomic datasets, LV > RV. The Venn diagrams in Supplementary Figure S3C show the total number of proteins that were differentially decreased or increased in abundance in ShLuc vs. Sham in both proteomic datasets. For simplicity, we will hereafter use the terms downregulated and upregulated. They also show that the mt-proteins were predominantly downregulated rather than upregulated in HFrEF and ShLuc vs. Sham, in both the LV (380 vs. 22) and RV (235 vs. 32) proteomic datasets. The Venn diagrams in Supplementary Figure S3D–F show the total number of proteins and mt-proteins that were differentially expressed in ShLuc vs. Sham, and how many of these were common between the LV and RV proteomic datasets. The data suggest that there were more differentially expressed proteins in ShLuc vs. Sham in LV vs. RV (1827 vs. 1355); 910 of these were common between both proteomic datasets. Moreover, the number of mt-proteins that were downregulated in ShLuc vs. Sham was greater in LV vs. RV (380 vs. 235); 205 of these were common between both proteomic datasets. Heat maps, PCA plots, and PPI networks of the 205 commonly identified mt-proteins that were differentially downregulated in ShLuc vs. Sham are presented in Supplementary Figure S5A–C. The PPI network shows three predominant clusters of protein networks involved in OXPHOS (green color), mt-metabolism (blue), and mt-28S and 39S ribosomal proteins (red). The bar graph in Supplementary Figure S5D (see also Supplementary Excel file S2) shows the enriched reactome pathways, by q-value, in the 205 commonly identified mt-proteins that were differentially downregulated in ShLuc vs. Sham.

**Figure 1:**
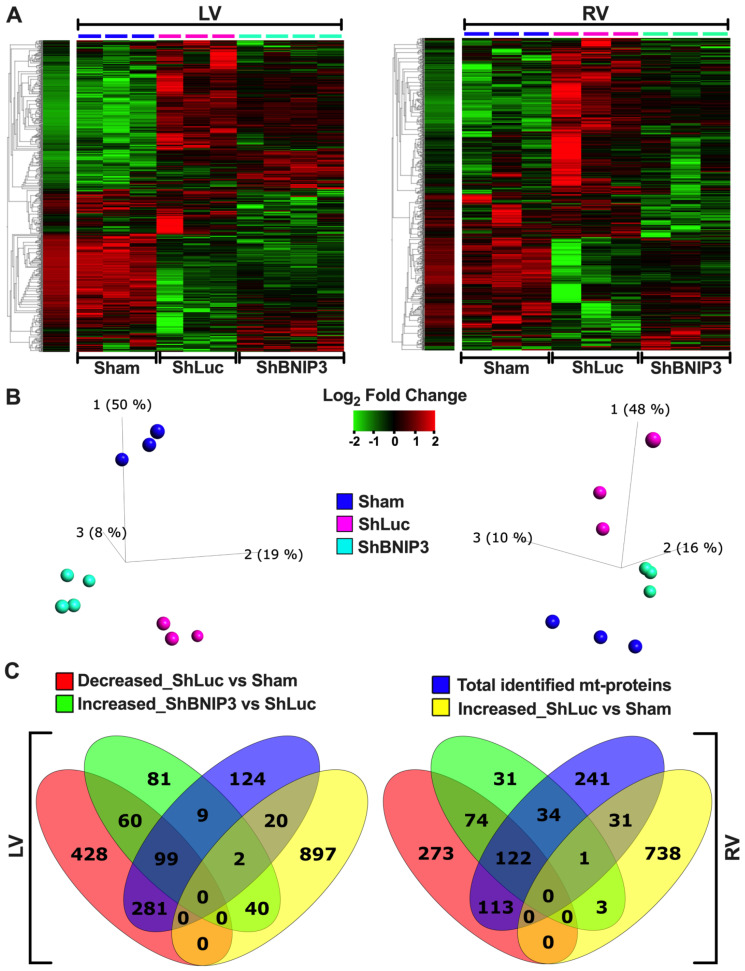

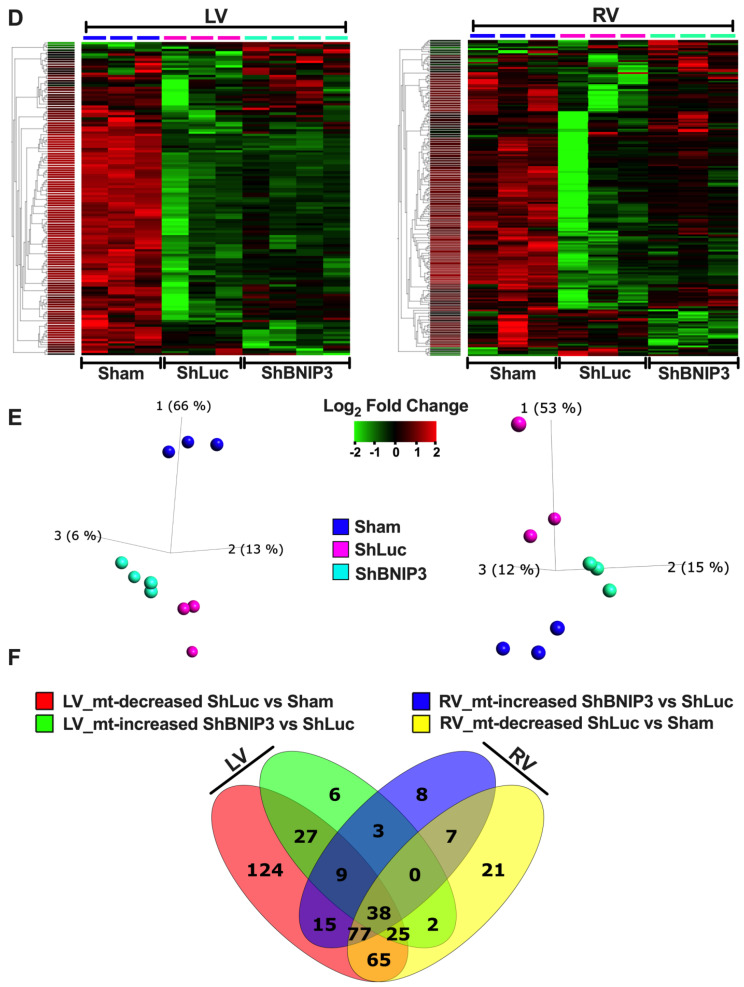
Visualization of the differentially expressed proteins in ShBNIP3 vs. ShLuc groups in the LV and RV myocardium proteomic datasets. (**A**,**B**). Heat maps and PCA plots show the relative log_2_-fold expression and the variance in biological samples, respectively, in Sham and HFrEF, and ShLuc and ShBNIP3 groups of the differentially expressed proteins in ShBNIP3 vs. ShLuc in LV (left) and RV (right) shotgun proteomic datasets. (**C**). Venn diagrams show the number of proteins that were differentially downregulated (red) or upregulated (yellow) in ShLuc vs. Sham in LV (left) and RV (right) shotgun proteomic datasets, and how many of these were mitochondrial (mt)-proteins (intersection of blue with red or yellow diagrams, respectively). They also show the number of proteins that were differentially upregulated in ShBNIP3 vs. ShLuc (green), and how many of these were mt-proteins (intersection of blue with green diagrams). (**D**,**E**). Similar to A and B, with the exception that the heat maps and PCA plots represent the differentially expressed mt-proteins in ShBNIP3 vs. ShLuc groups. (**F**). Venn diagrams show the number of mt-proteins that were differentially downregulated in ShLuc vs. Sham (red and yellow), and those that were differentially upregulated in ShBNIP3 vs. ShLuc (green and blue) in LV and RV shotgun proteomic datasets, respectively. Note: the stand-alone column in the presented heat maps shows the hierarchical clustering of variables and the average log_2_-fold change in Sham biological samples. This is applicable for all the subsequently presented heat maps.

Visualization of the differentially expressed proteins and mt-proteins in ShBNIP3 vs. ShLuc is shown in Figure 1A–F, respectively. The PCA plots (Figure 1B,E) show that the ShBNIP3 biological samples are now more separated from the ShLuc biological samples, and are getting closer to Sham in the RV > LV proteomic datasets. This suggests an overall beneficial effect in the ShBNIP3-treated HFrEF group. The Venn diagrams in Figure 1C and Figure S4A show the number of proteins and mt-proteins that were differentially upregulated (Figure 1C) or downregulated (Supplementary Figure S4A) in ShBNIP3 vs. ShLuc in relation to those that were differentially downregulated and upregulated in ShLuc vs. Sham. The data suggest a predominant opposite directional change in ShBNIP3 vs. ShLuc toward Sham in both proteomic datasets, RV > LV. For example, in the LV proteomic dataset, out of the 291 proteins (110 mt-proteins) that were differentially upregulated in ShBNIP3 vs. ShLuc, 159 (99) and 42 (2) of these were downregulated and upregulated, respectively, in ShLuc vs. Sham; meanwhile, the remaining 90 (9) proteins did not change in ShLuc vs. Sham. Similarly, in the RV proteomic dataset, out of the 265 proteins (157 mt-proteins) that were differentially upregulated in ShBNIP3 vs. ShLuc, 196 (122) and 4 (1) of these were downregulated and upregulated, respectively, in ShLuc vs. Sham, whereas the remaining 65 (34) proteins did not change in ShLuc vs. Sham. The Venn diagrams in Supplementary Figure S4D–F show the total number of proteins and mt-proteins that were differentially abundant in ShBNIP3 vs. ShLuc, as well as the proteins/mt-proteins detected in common between the LV and RV proteomic datasets. The data show that the mt-proteins were predominantly differentially upregulated, rather than downregulated, in ShBNIP3 vs. ShLuc in both proteomic datasets, RV > LV. The Venn diagrams in Figure 1F show the number of mt-proteins that were downregulated in ShLuc vs. Sham, and how many of these increased in ShBNIP3 vs. ShLuc, in both proteomic datasets. Out of the 380 vs. 235 mt-proteins that were differentially downregulated in ShLuc vs. Sham in LV vs. RV, 99 vs. 122 of these were differentially upregulated in ShBNIP3 vs. Sham. Moreover, there were 50 common mt-proteins that were differentially upregulated in both proteomic datasets. Their respective heat maps, PCA plots, and PPI networks are presented in Supplementary Figure S5E–G. The bar graph in Supplementary Figure S5H (see also Supplementary Excel file S2) shows the enriched reactome pathways, by q-value, in the 50 commonly identified mt-proteins that were differentially upregulated in ShBNIP3 vs. ShLuc.

### 3.3. Validation of the LV and RV Proteomic Findings

**Figure 2:**
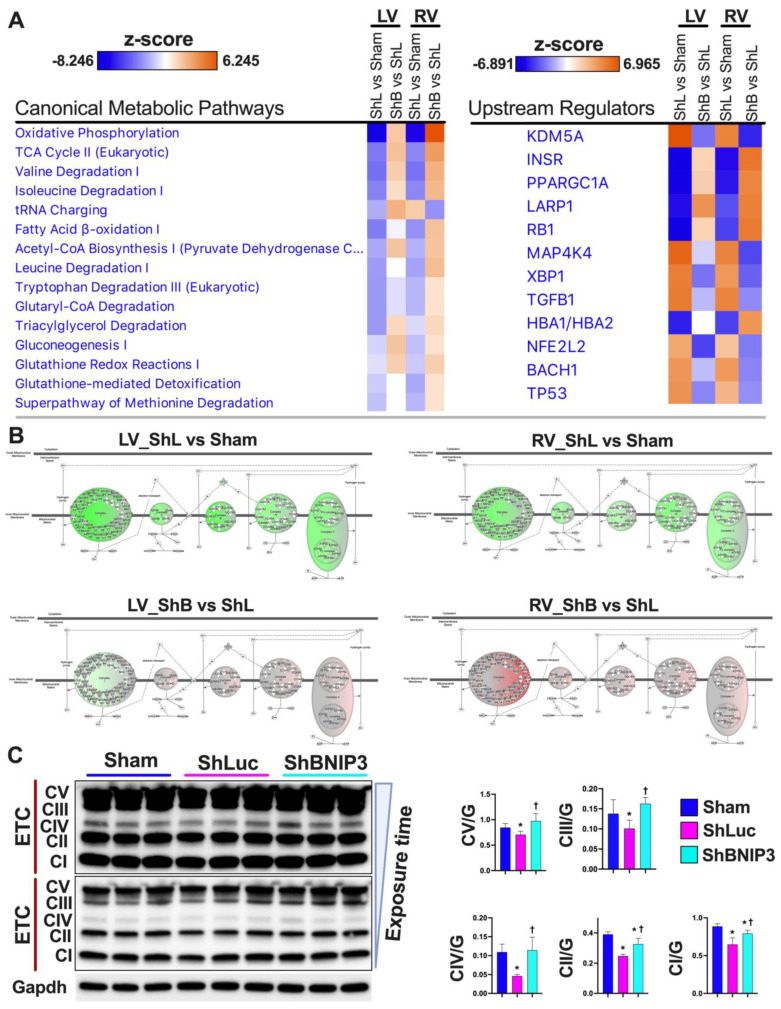

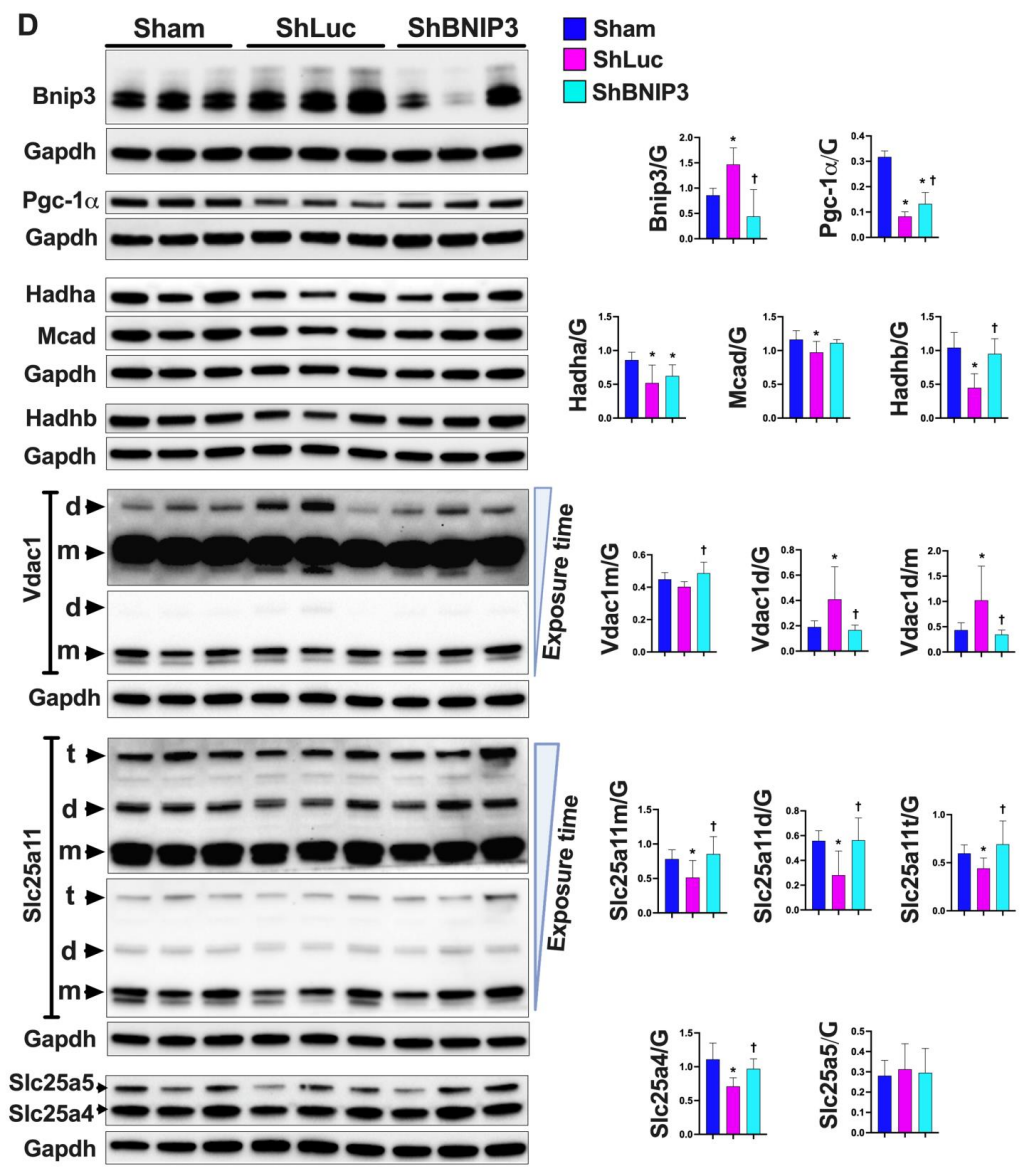
Validation of the LV and RV proteomic findings. (**A**). “Core Analyses” was performed in IPA for each of the two-group comparisons, i.e., ShLuc vs. Sham and ShBNIP3 vs. ShLuc, with a cutoff *p*-value < 0.05. Analyzed datasets were then compared with each other in IPA’s “Comparison Analyses” function to yield the most enriched *Canonical Metabolic Pathways* (left) and *Upstream Regulators* (right) that were shared among the two-group comparisons. The respective z-score-based heat maps indicate shared *Canonical Pathways* and *Upstream Regulators* that were upregulated/activated or downregulated/inhibited in the two-group comparisons, with orange and blue color intensities representing the z-score-based extent of upregulation/activation or downregulation/inhibition, respectively. (**B**). Oxidative phosphorylation (OXPHOS) network of the electron transport chain (ETC) complexes I–V transport system presented in ShLuc vs. Sham (upper panel) and ShBNIP3 vs. ShLuc (lower panel) in LV (left) and RV (right) proteomic datasets. The network highlights mt-OXPHOS proteins in each ETC complex that were downregulated (green) or upregulated (red) or were unchanged (grey) within the two-group comparisons, and those that were not identified (white) in the LV and RV proteomic datasets. The darker the green or red color, the higher the degree of decrease or increase in relative expression, respectively. (**C**,**D**). Expression of the ETC complexes I–V, BNIP3, and molecular markers involved in mt-biogenesis and metabolism by western blot, * *p* < 0.05 vs. Sham and ^†^ *p* < 0.05 vs. ShLuc. Abbreviations: ShL, ShLuc; ShB, ShBNIP3.

Validation of the proteomic findings was performed in IPA bioinformatics software using the IPA Knowledge Base database. Heat maps of the metabolic pathways and upstream regulators that were upregulated/activated or downregulated/inhibited in the two-group comparisons (ShLuc vs. Sham and ShBNIP3 vs. ShLuc) in the LV and RV proteomic datasets are presented in Figure 2A. The orange and blue color intensities represent the z-score-based extent of upregulation/activation or downregulation/inhibition, respectively. A larger list of the canonical pathways and upstream regulators that were upregulated/activated or downregulated/inhibited in the two-group comparisons are shown in Supplementary Figure S6A–B. The data are presented from the highest to the lowest degree of downregulation or upregulation by z-score. OXPHOS, tricarboxylic acid (TCA) cycle, branched-chain amino acid (BCAA) catabolism, and pyruvate metabolism were among the highest metabolic pathways that were downregulated in ShLuc vs. Sham and improved in ShBNIP3 vs. ShLuc, RV > LV. OXPHOS network of the electron transport chain (ETC) complexes I–V (Figure 2B) show that all five ETC complexes were downregulated in ShLuc vs. Sham, LV > RV, and that their expression increased in ShBNIP3 vs. ShLuc, RV > LV (especially for complex I). The darker the green or red color intensity, the higher the degree of downregulation or upregulation, respectively. Western blot of the ETC complexes validates the above proteomic findings (Figure 2C). Other metabolic pathways, such as gluconeogenesis, triacylglycerol (TAG) degradation, and glutathione redox reactions and detoxification, were also downregulated in ShLuc vs. Sham and improved in ShBNIP3 vs. ShLuc. The effect of BNIP3 knockdown on fatty acid (FA) β-oxidation and amino acid degradation was ambivalent in LV and RV proteomic datasets. It is most likely that the improvement seen in RV, but not in LV, for these metabolic pathways to be related overall to improvement in RV function secondary to improvement in LV function and not to a direct effect of BNIP3 on these metabolic pathways. Western blot (Figure 2D and Figure S6C) show BNIP3 expression in Sham and HFrEF groups in LV and RV myocardia, respectively. Western blot analysis of the enzymes involved in mt-FA β-oxidation (Hadha, Hadhb, and Mcad) (Figure 2D) showed that all were downregulated in ShLuc vs. Sham; however, there was some improvement in the expression of the mt-trifunctional enzyme, Hadhb, in ShBNIP3 vs. ShLuc. BNIP3 knockdown in HFrEF attenuated the dimerization of the voltage-dependent anion channel isoform 1 (Vdac1) rather than a change in its expression (as suggested by the proteomic dataset), and improved the expression of the mt-2-oxoglutarate/malate carrier protein (Slc25A11) and the ADP/ATP translocase isoform1 (Slc25A4), but not isoform 2 (Slc25A5) (Figure 2D). BNIP3 knockdown in HFrEF also attenuated the activity of the eukaryotic translation initiation factor 2 (EIF2), Rho family GTPases and RhoA, mammalian target of rapamycin (mTOR), and mitogen-activated protein kinase (MAPK) signaling, as well as ER stress and unfolded protein response (UPR) pathways that were all upregulated in ShLuc vs. Sham.

Regarding the upstream regulators, BNIP3 knockdown in HFrEF enhanced the activity of the peroxisome proliferator-activated receptor gamma coactivator 1-alpha (PGC-1α), La-related protein 1 (LARP1), and the microRNAs miR-30c-5p, miR-155-5p, and miR-16-5p, whose activities were attenuated in ShLuc vs. Sham. BNIP3 knockdown in HFrEF also attenuated the activity of the lysine-specific demethylase 5A (KDM5A), MAPK kinase 4 (MAP4K4), X-box-binding protein 1 (XBP1), transforming growth factor beta-1 (TGF-β1), transcription regulator protein BACH1, cell tumor antigen p53 (TP53), and the microRNA miR-9-5p. A western blot analysis of the molecular marker PGC-1α (Figure 2D) showed that its expression was decreased in ShLuc vs. Sham, and mildly increased in ShBNIP3 vs. ShLuc. Collectively, these data suggest that BNIP3, in addition to regulating cardiac metabolism, affects the pathways and upstream regulators involved in cardiac transcription and translation regulation, cardiac growth, pathological myocardial remodeling and extracellular matrix remodeling, and apoptosis.

### 3.4. Effect of BNIP3 on Mitochondrial Function and Respiration in Cardiac Myocytes

**Figure 3:**
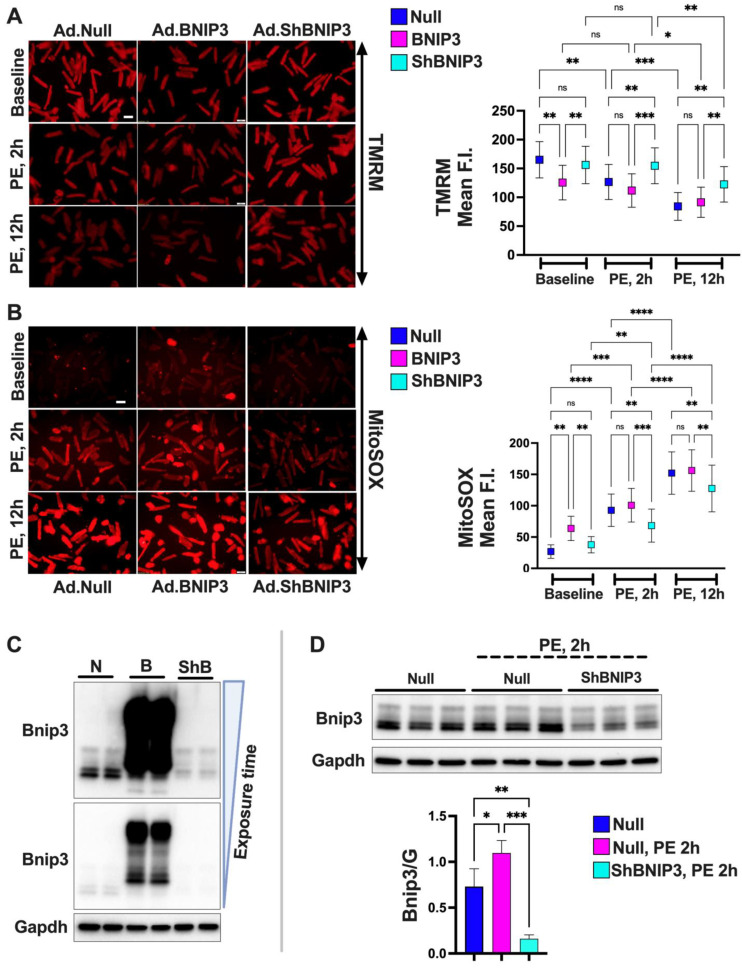

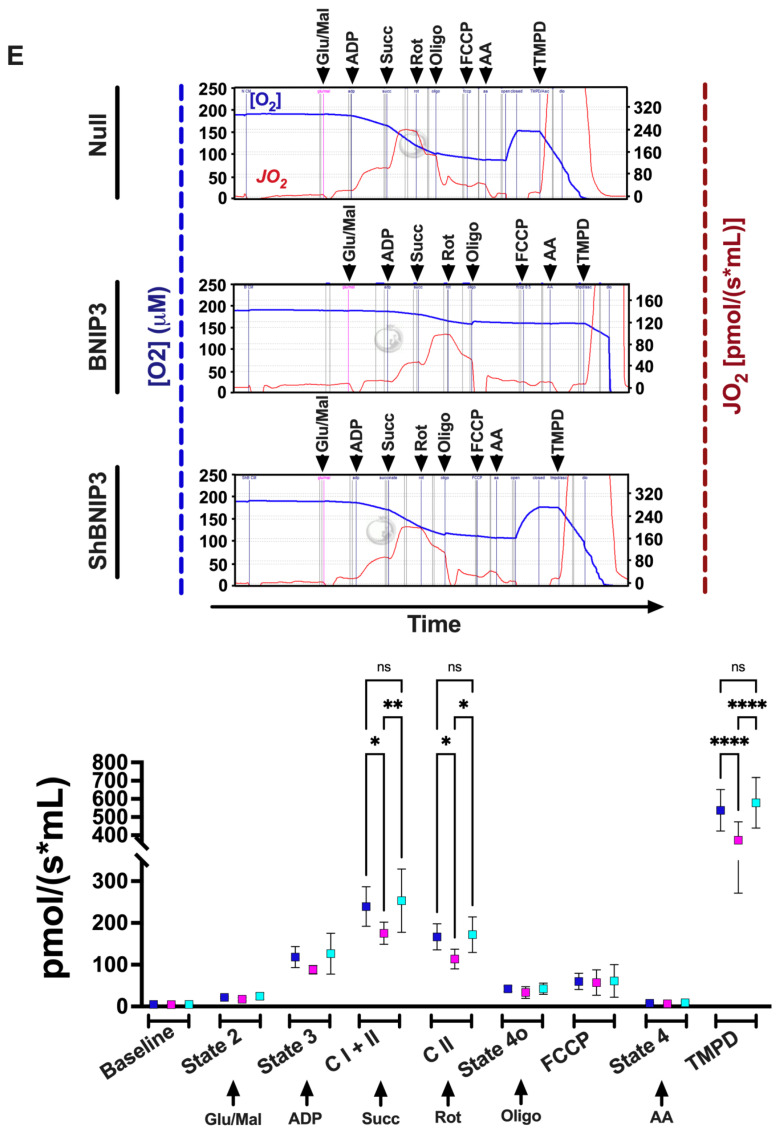
BNIP3 enhances mt-reactive oxygen species production, and attenuates mt-membrane potential and respiration, in cultured adult cardiomyocytes in vitro. (**A**,**B**). Representative fluorescent photomicrographs in cultured adult cardiac myocytes (ACM) transfected with adenovirus encoding for Null, BNIP3, or BNIP3 ShRNA (ShBNIP3), and loaded with the fluorophores TMRM and MitoSOX to assess mt-membrane potential and mt-reactive oxygen species, respectively. Live imaging was acquired at baseline and after 2 and 12 h of phenylephrine (PE, 10 μM) stress. Images are 20× magnified, scale bar 50 μm. Data were obtained from at least 3 independent experiments, and are presented as mean ± SD, * *p* < 0.05, ** *p* < 0.01, *** *p* < 0.001, **** *p* < 0.0001. (**C**,**D**). Western blots show BNIP3 expression in normal (**C**) and in PE-stressed (**D**) ACM transfected with adenovirus Null (N), BNIP3 (B) or ShBNIP3 (ShB), * *p* < 0.05, ** *p* < 0.01, *** *p* < 0.001. (**E**). Representative tracings of mt-respiration by Oroboros oxygraph-2k system in permeabilized ACM transfected with adenovirus Null, BNIP3, or ShBNIP3. Data were obtained from 7 independent experiments and are presented as mean ± SD, * *p* < 0.05, ** *p* < 0.01, **** *p* < 0.0001. Abbreviations: F.I., fluorescence intensity; ns, not significant; Glu/Mal, glutamate/malate; ADP, adenosine diphosphate; Succ, succinate; Rot, rotenone; Oligo, oligomycin; FCCP, carbonyl cyanide 4-(trifluoromethoxy)phenylhydrazone; AA, Antimycin A; TMPD, N,N,N′,N′-tetramethyl-p-phenylenediamine dihydrochloride.

To further validate the effect of BNIP3 on mt-function, i.e., mt-membrane potential and mt-reactive oxygen species (ROS) production, we conducted functional studies in isolated ACM in vitro using the fluorophores TMRM and MitoSOX. BNIP3 overexpression (OE) in ACM, under baseline/normal conditions, attenuated mt-membrane potential and enhanced the production of mt-ROS vs. Ad-Null (control group) (Figure 3A,B). Moreover, BNIP3 knockdown attenuated the decrease in mt-membrane potential and the increase in mt-ROS in PE-stressed ACM vs. PE–Ad-Null and PE–Ad-BNIP3 groups (Figure 3A,B). The protective effect of BNIP3 knockdown was sustained at 2 h and 12 h post-PE stress, PE, 2 h > PE, 12 h. Western blot analysis (Figure 3C,D) shows the increase in BNIP3 expression in Ad-BNIP3 and PE–Ad-Null groups, as well as BNIP3 knockdown in Ad-ShBNIP3 and PE–Ad-ShBNIP3 groups. The mt-respiration studies in ACM (Figure 3E) showed that BNIP3 OE attenuated mt-respiration by primarily affecting the activity of the ETC complexes II and IV, and to some extent complex I, compared to Ad-Null and Ad- ShBNIP3 groups. There was no change in citrate synthase activity or mt-content in all three groups (data not shown).

### 3.5. Visualization and Validation of the Untargeted Metabolomic Dataset in LV Myocardia

**Figure 4:**
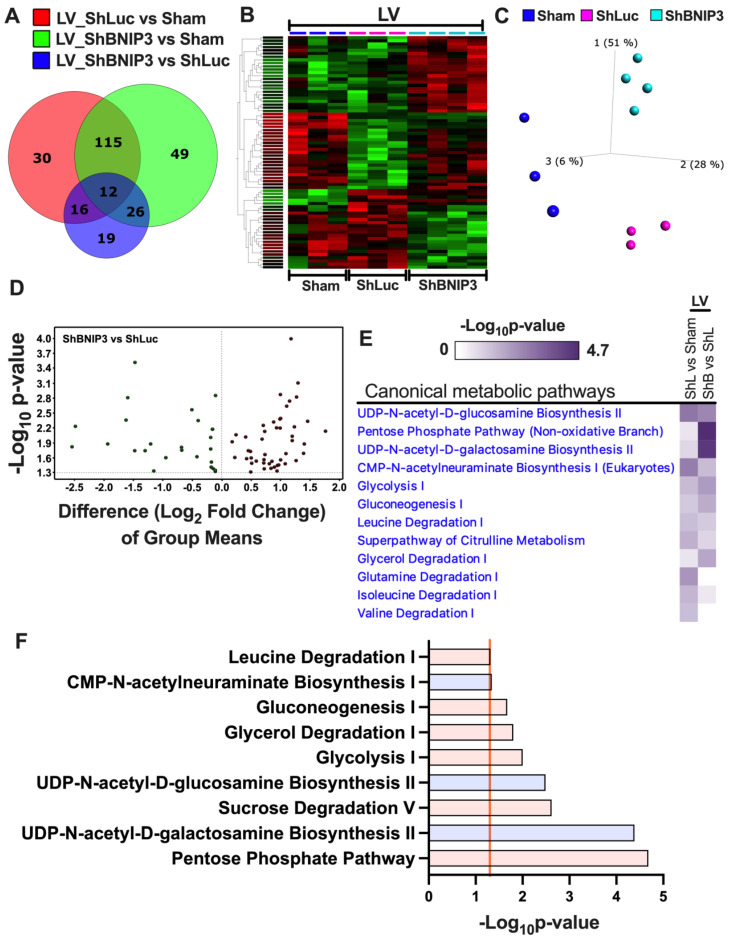

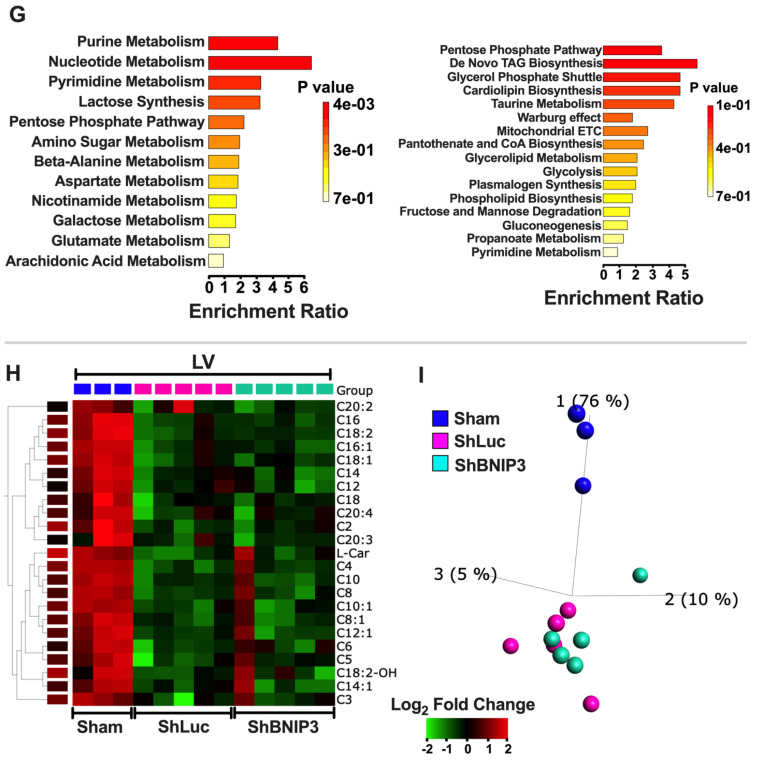
Visualization and validation of the differentially expressed metabolites in ShBNIP3 vs. ShLuc groups in the LV myocardium untargeted metabolomic dataset. (**A**). Venn diagrams show the number of metabolites that were differentially abundant in ShLuc vs. Sham (red), ShBNIP3 vs. Sham (green), and ShBNIP3 vs. ShLuc (blue). (**B**,**C**). Heat maps and PCA plots show the relative log_2_-fold expression and the variance in biological samples, respectively, in Sham, ShLuc, and ShBNIP3 groups of the differentially abundant metabolites in ShBNIP3 vs. ShLuc groups. (**D**). Volcano plot shows the log_2_-fold change in group means for the metabolites whose abundance increased (red) or decreased (green) in the ShBNIP3 vs. ShLuc groups, taking a cutoff −Log_10_ *p*-value of 1.3 (*p* < 0.05). (**E**). Heat maps show the most enriched *Metabolic Pathways* by the −Log_10_ *p*-value of the untargeted metabolomics dataset. The heat maps were generated in IPA after “Core Analyses” of each of the two-group comparisons, which were then compared with each other in IPA’s “Comparison Analyses” function. (**F**). Metabolic pathways that were predicted to be enhanced (orange bar) or attenuated (blue bar) in shBNIP3 vs. ShLuc, taking a cutoff −Log_10_ *p*-value of 1.3 (orange line). (**G**). Enrichment pathway analysis of the metabolites that were differentially downregulated (left) or upregulated (right) in ShBNIP3 vs. ShLuc. Data were generated in MetaboAnalyst 5.0 bioinformatics software using the “Enrichment Analysis” function. (**H**,**I**). Heat maps and PCA plots show the relative log_2_-fold expression and the variance in biological samples, respectively, in Sham, ShLuc, and ShBNIP3 groups of the differentially abundant acyl-carnitines in ShBNIP3 and ShLuc groups vs. Sham.

The metabolites that were differentially abundant in ShLuc vs. Sham and ShBNIP3 vs. ShLuc are presented in Supplementary Excel file S3. The Venn diagrams in Figure 4A show the total number of metabolites that were differentially abundant in ShLuc vs. Sham, ShBNIP3 vs. Sham, and ShBNIP3 vs. ShLuc. Heat maps and PCA plots (Supplementary Figure S7A,B) show the relative log_2_-fold change and the variance in biological samples in Sham, ShLuc, and ShBNIP3 groups of the differentially abundant metabolites in ShLuc vs. Sham. The PCA plot indicates that the highest variance exists between Sham and the HFrEF biological samples. Moreover, the ShBNIP3 biological samples are separated from the ShLuc biological samples, and are closer to Sham, suggesting the improvement of some of these metabolites in the ShBNIP3 group toward Sham. Heat maps and PCA plots (Figure 4B,C) show the relative log_2_-fold change and the variance in biological samples in Sham, ShLuc, and ShBNIP3 groups of the differentially abundant metabolites in ShBNIP3 vs. ShLuc. The PCA plot indicates that, for these metabolites, the highest variance exists between ShBNIP3 and ShLuc biological samples, with Sham being midway between the HFrEF groups. Volcano plots (Supplementary Figure S7C and Figure 4D) show the log_2_-fold change in group means for the metabolites that were differentially upregulated or downregulated in ShLuc vs. Sham and ShBNIP3 vs. ShLuc, respectively. The heat map in Figure 4E shows the metabolic pathways that were enriched by *p*-value in the two-group comparisons: ShLuc vs. Sham and ShBNIP3 vs. ShLuc. The bar graphs in Supplementary Figure S7D and Figure 4F show the metabolic pathways that were enriched by *p*-value, and their predicted activation (orange bars) or inhibition (blue bars), in ShLuc vs. Sham and ShBNIP3 vs. ShLuc, respectively. UDP-N-acetyl-D-glucosamine biosynthesis, CMP-N-acetylneuraminate biosynthesis, and glutamine degradation were predicted to be activated, while as citrulline metabolism, glycolysis, and BCAA catabolism were predicted to be inhibited/downregulated, in ShLuc vs. Sham. The glucosamine and acetylneuraminate biosynthesis pathways were inhibited in ShBNIP3 vs. ShLuc. Moreover, BNIP3 knockdown in HFrEF was associated with the upregulation of the pentose phosphate pathway, sucrose degradation, leucine degradation, and glycolysis. Collectively, these results are in agreement, and complement the directional changes of the enriched metabolic pathways seen in the proteomic datasets. Enrichment pathway analysis (Figure 4G), using MetaboAnalyst bioinformatics software, of the metabolites that were differentially downregulated or upregulated in ShBNIP3 vs. ShLuc, showed that nucleotide metabolism was attenuated, while as TAG and cardiolipin biosynthesis, and taurine metabolism, were enhanced in ShBNIP3 vs. ShLuc. The heat map and PCA plot in Figure 4H,I show the short-, medium-, long-, and very long-chain fatty-acyl-carnitines that were about equally differentially downregulated in ShLuc and ShBNIP3 vs. Sham, without a significant change in ShBNIP3 vs. ShLuc for these metabolites.

### 3.6. Visualization and Validation of the LV and RV Phosphoproteomic Datasets

**Figure 5:**
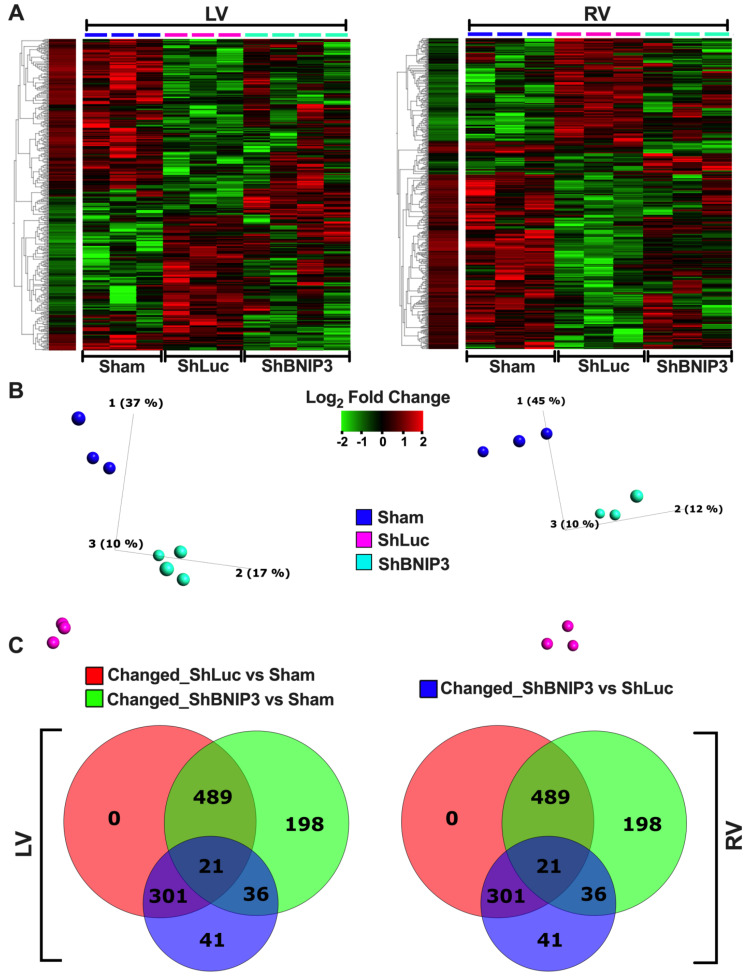

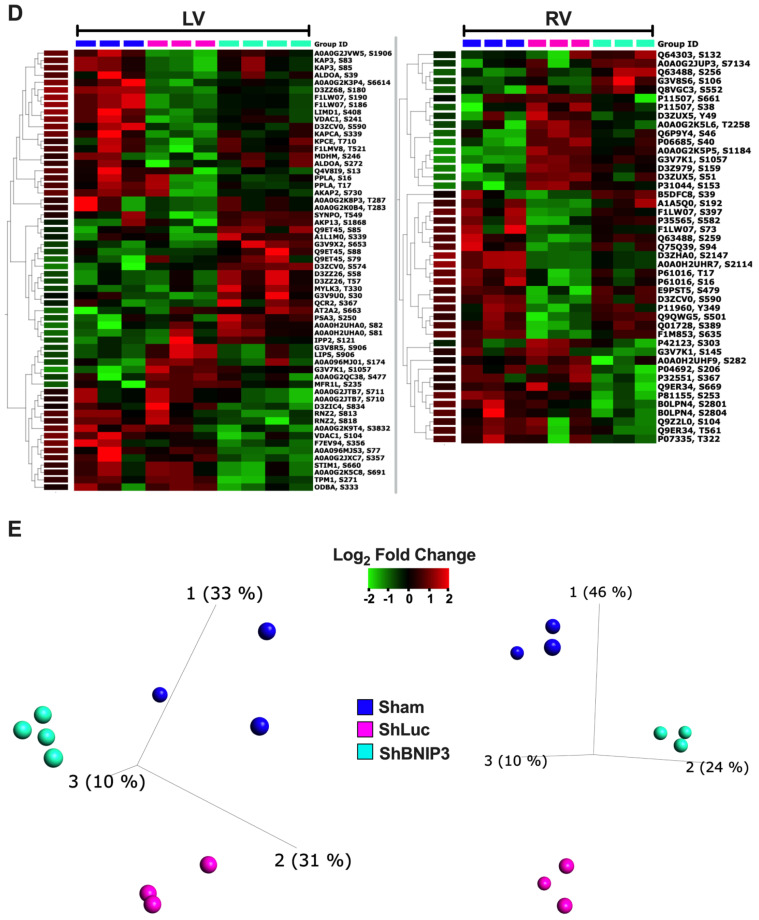
Visualization of the differentially expressed phosphosites in ShBNIP3 vs. ShLuc groups in the LV and RV myocardium p-proteomic datasets. (**A**,**B**). Heat maps and PCA plots show the relative log_2_-fold expression and the variance in biological samples, respectively, in Sham, ShLuc, and ShBNIP3 groups of the differentially expressed p-sites in ShBNIP3 vs. ShLuc groups in LV (left) and in RV (right) shotgun phosphoproteomic dataset© (**C**). Venn diagrams show the number of differentially expressed phosphosites in ShLuc vs. Sham (red), ShBNIP3 vs. Sham (green), and ShBNIP3 vs. ShLuc (blue) in LV (left) and RV (right) shotgun p-proteomic datasets. (**D**,**E**). Heat maps and PCA plots show the relative log_2_-fold expression and the variance in biological samples, respectively, in Sham, ShLuc, and ShBNIP3 groups of some of the important differentially expressed phosphosites in ShBNIP3 vs. ShLuc groups in LV (left) and RV (right) shotgun phosphoproteomic datasets.

The differentially expressed proteins in ShLuc vs. Sham and ShBNIP3 vs. ShLuc in LV and RV phosphoproteomic datasets are shown in Supplementary Excel file S4. Heat maps and PCA plots (Supplementary Figure S8A,B) show the differentially expressed phosphosites in ShLuc vs. Sham. Similar to the proteomic datasets, the highest variance is between Sham and the HFrEF biological samples. Moreover, the ShBNIP3 biological samples are separated from ShLuc, and are closer to Sham, RV > LV, suggesting a differential directional change in some of these phosphosites toward Sham in the ShBNIP3-treated group. Heat maps and PCA plots (Figure 5A,B) show the differentially expressed phosphosites in ShBNIP3 vs. ShLuc. The PCA plots for these phosphosites show a significant improvement and separation of the ShBNIP3 biological samples toward Sham, suggesting an overall beneficial effect of BNIP3 knockdown in HFrEF, RV > LV. The Venn diagrams in Figure 5C show the total number of differentially expressed phosphosites in ShLuc vs. Sham, ShBNIP3 vs. Sham, and ShBNIP3 vs. ShLuc. The heat maps and PCA plots in Figure 5D,E highlight the important phosphosites that were differentially expressed in ShBNIP3 vs. ShLuc, in the LV and RV phosphoproteomic datasets. In both phosphoproteomic datasets, BNIP3 knockdown in HFrEF affected the phosphorylation of proteins involved in calcium cycling and homeostasis, protein kinases A (PkA) and C (PKC), tyrosine protein phosphatases and protein phosphatase 1, sarcomeric and cytoskeletal proteins, and mt-proteins involved in metabolism and OXPHOS (Supplementary Excel files S4 and S5). More importantly, BNIP3 knockdown in HFrEF enhanced the phosphorylation of BNIP3 at serine s 79, 85, and 88 residues, and VDAC1 at serine 241 residue, and attenuated the phosphorylation of VDAC1 at serine 104 residue.

**Figure 6:**
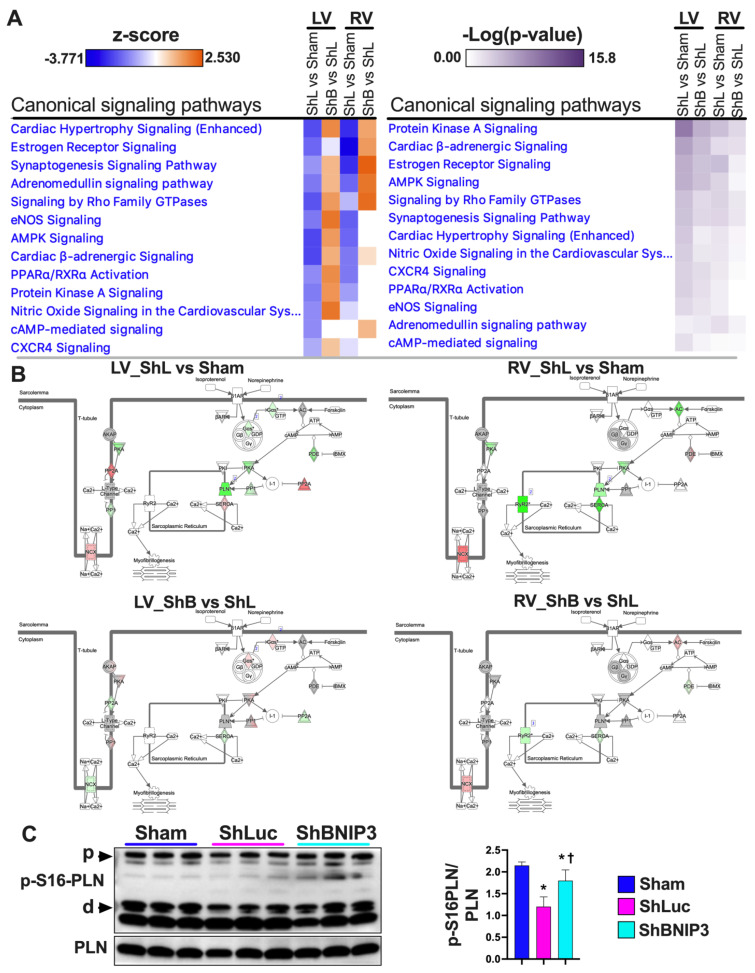

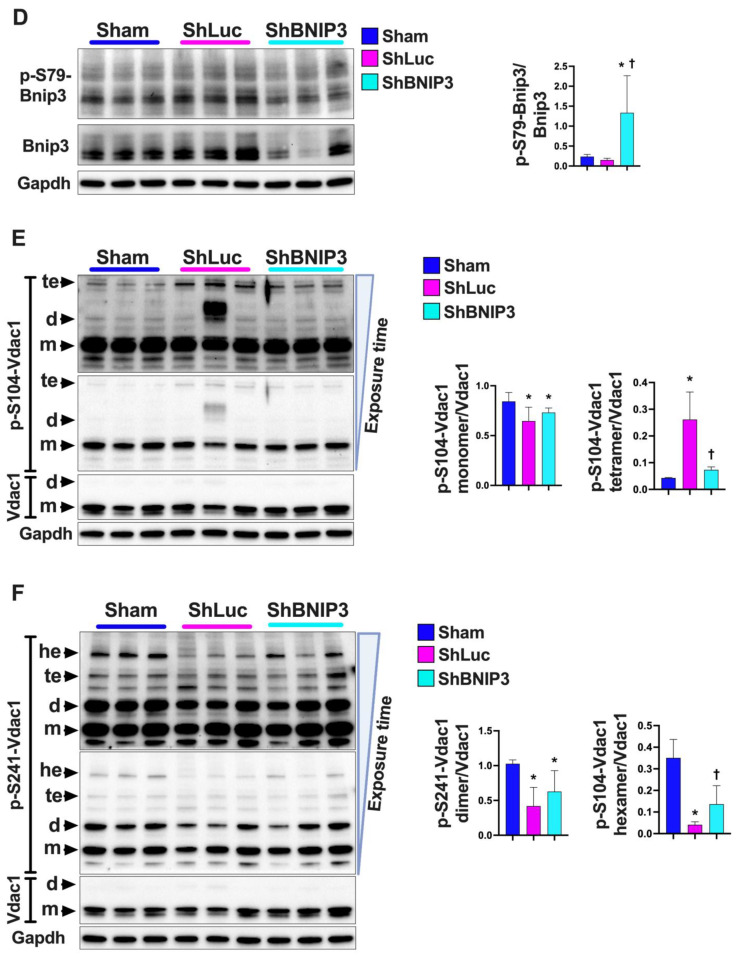
Validation of the LV and RV phosphoproteomic findings. (**A**). Heat maps of the *Canonical signaling pathways* that were enriched by −Log_10_ *p*-value in the phosphoproteomic datasets. The orange and blue color intensities represent the z-score-based extent of upregulation/activation or downregulation/inhibition, respectively. The heat maps were generated in IPA after “Core Analyses” of each of the two-group comparisons, which were then compared with each other in IPA’s “Comparison Analyses” function. (**B**). *Cardiac* *β-adrenergic signaling* network of the phosphoproteins/phosphosites in ShLuc vs. Sham (upper panel) and ShBNIP3 vs. ShLuc (lower panel) in LV (left) and RV (right) phosphoproteomic datasets. The network highlights phosphoproteins/phosphosites that were differentially downregulated (green) or upregulated (red) or were unchanged (grey) in the two-group comparisons, and those that were not identified (white) in the LV and RV phosphoproteomic datasets. The darker the green or red color, the higher the degree of decrease or increase in relative expression, respectively. (**C**). Western blot validating the change in phospholamban (PLN) phosphorylation at serine 16 residue (p-S16-PLN) in Sham, ShLuc, and ShBNIP3 experimental groups, * *p* < 0.05 vs. Sham and ^†^ *p* < 0.05 vs. ShLuc. (**D**–**F**). Western blot validating the change in BNIP3 and VDAC1 phosphorylation at serine 79 (p-S79-BNIP3), serine 104 (p-S104-VDAC1) and serine 241 (p-S241-VDAC1) residues, respectively, in Sham, ShLuc, and ShBNIP3 experimental groups, * *p* < 0.05 vs. Sham and ^†^ *p* < 0.05 vs. ShLuc. Abbreviations: p, pentamer; m, monomer; d, dimer; te, tetramer; he, hexamer.

Validation of the phosphoproteomic datasets was performed using IPA’s Knowledge Base database. Heat maps (Figure 6A) show the signaling pathways that were enriched by *p*-value, and the degree of their upregulation/activation or downregulation/inhibition by z-score, in the two-group comparisons (ShLuc vs. Sham and ShBNIP3 vs. ShLuc) in the LV and RV phosphoproteomic datasets. The orange and blue color intensities represent the z-score-based extent of upregulation/activation or downregulation/inhibition, respectively. PKA, cardiac β-adrenergic, and AMPK signaling were amongst the highest enriched signaling pathways in the two-group comparisons, LV > RV. These signaling pathways were predicted to be downregulated in ShLuc vs. Sham and upregulated in ShBNIP3 vs. ShLuc, LV > RV. Cardiac β-adrenergic networks (Figure 6B) show the phosphosites/phosphoproteins that were upregulated (red) or downregulated (green) in ShBNIP3 vs. ShLuc in both the LV and RV phosphoproteomic datasets. The phosphorylation of phospholamban at serine 16 residue (Figure 6C) was attenuated in ShLuc vs. Sham, and increased in ShBNIP3 vs. ShLuc. The phosphorylation of AMPK at Thr172 residue, i.e., the enhancement in its enzymatic activity (Supplementary Figure S9), was unchanged in the Sham and the HFrEF groups, while its phosphorylation at serine 485/491 residues, i.e., the inhibition of its enzymatic activity, was increased in ShLuc vs. Sham, and was attenuated in ShBNIP3 vs. ShLuc (Supplementary Figure S9). This suggests a net decrease in AMPK activity in ShLuc vs. Sham and an increase in its activity in ShBNIP3 vs. ShLuc. The phosphorylation of downstream PKA targets (p-S21-GSK3α (glycogen synthase kinase-3 alpha), p-S9-GSK3β (glycogen synthase kinase-3 beta), and p-S23/24-TNNI3 (troponin I, cardiac muscle)) at different cellular subcompartments was attenuated in ShLuc vs. Sham, and did not differ significantly in ShBNIP3 vs. ShLuc (Supplementary Figure S9). The p-S23/24-TNNI3 uptrended in ShBNIP3 vs. ShLuc, but did not reach statistical significance. The expression of protein phosphatase 1a (PP1a), a serine/threonine phosphatase that antagonizes PKA enzymatic activity, was increased in ShLuc and ShBNIP3 vs. Sham (Supplementary Figure S9). Collectively, these data suggest an overall improvement in AMPK activity in ShBNIP3 vs. ShLuc, whereas the improvement in PKA activity was more confined to the ER–mt interface subcompartment.

Validation of the phosphoproteomic findings of BNIP3 phosphorylation at serine 79 residue and VDAC1 at serine 104 and 241 residues, respectively, by immunoblotting is shown in Figure 6D–F. There was no significant change in p-S79-BNIP3 to total BNIP3 ratio in ShLuc vs. Sham, whereas it was enhanced in ShBNIP3 vs. ShLuc (Figure 6D). The p-S104-VDAC1 monomer to total VDAC1 ratio was downregulated in both ShLuc and ShBNIP3 groups vs. Sham; meanwhile, the p-S104-VDAC1 tetramer to total VDAC1 ratio increased in ShLuc vs. Sham, and was attenuated in ShBNIP3 vs. ShLuc. The p-S241-VDAC1 dimer to total VDAC1 ratio decreased in ShLuc and ShBNIP3 vs. Sham, whereas the p-S241-VDAC1 hexamer to total VDAC1 ratio decreased in ShLuc vs. Sham and increased in ShBNIP3 vs. ShLuc.

### 3.7. Visualization and Validation of BNIP3 Interactome in Rat and Human LV Myocardia

**Figure 7:**
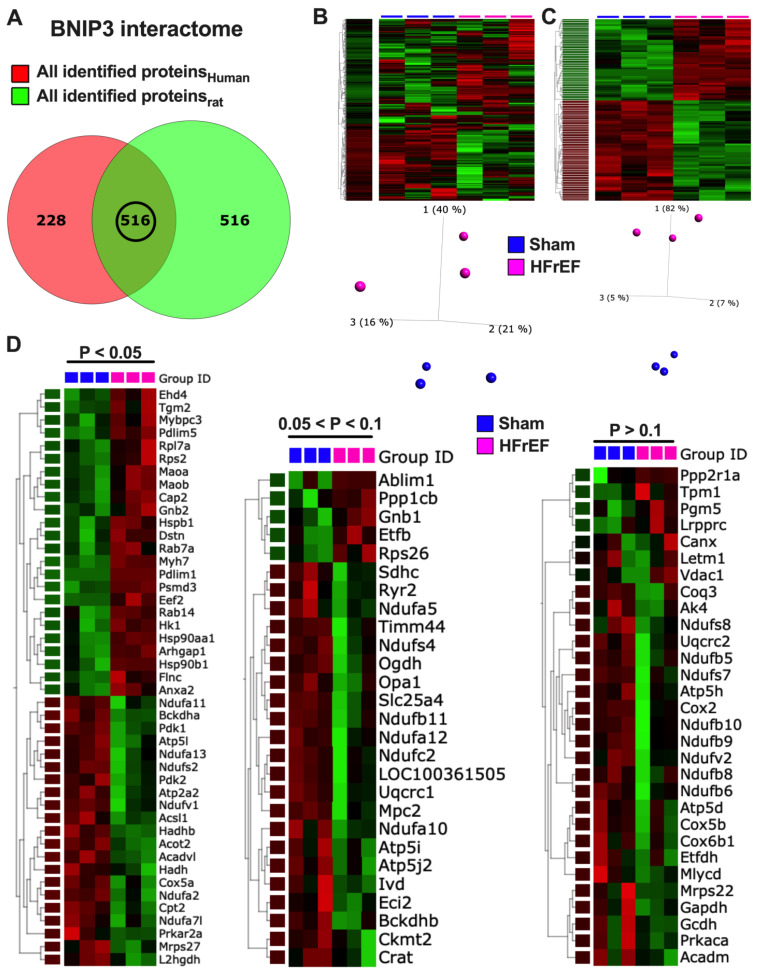

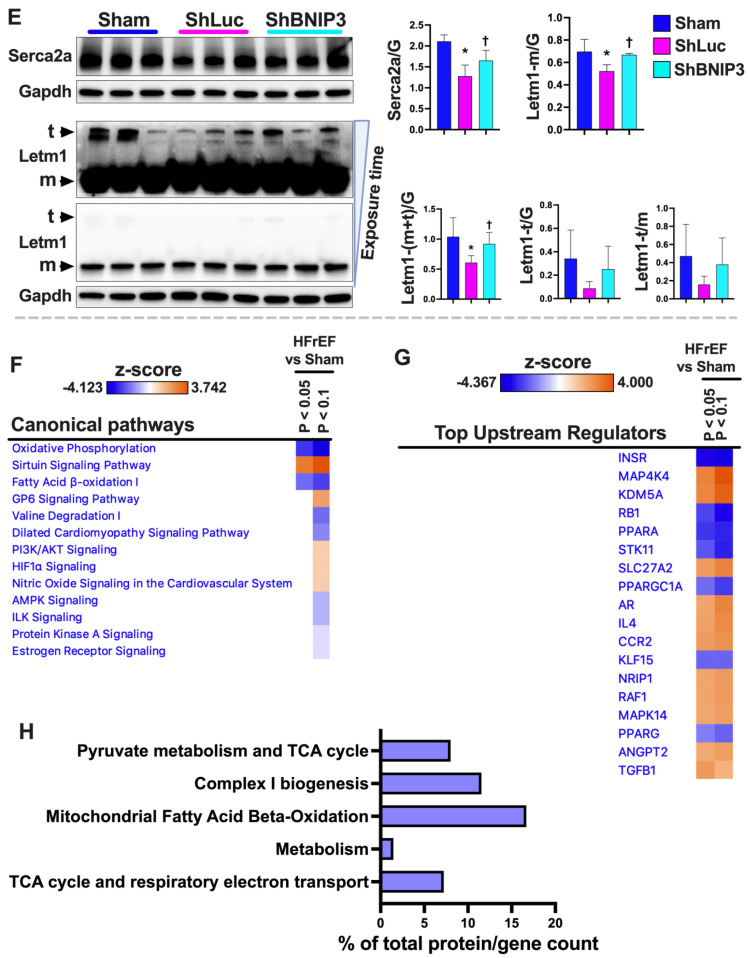

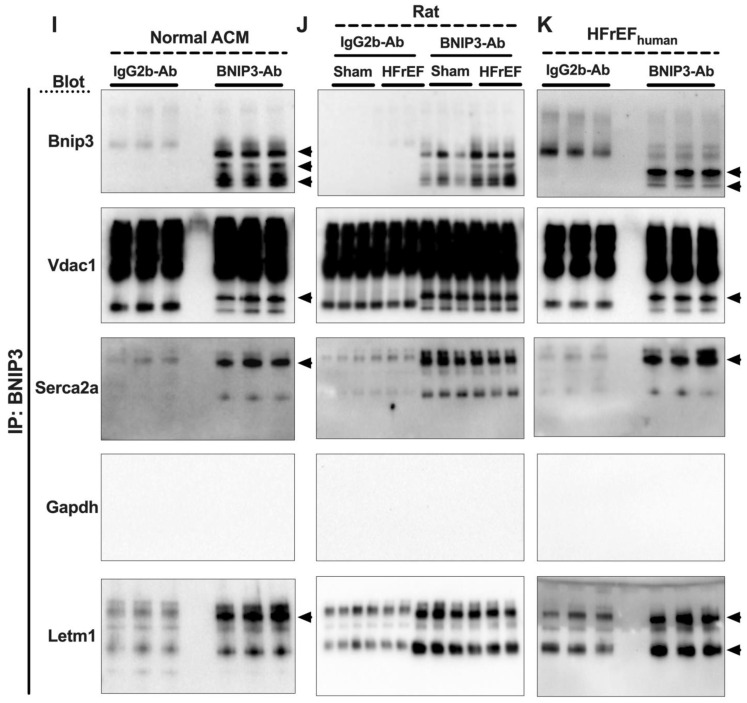
BNIP3 interactome in human and rat LV myocardia identified via BNIP3 co-immunoprecipitation (Co-IP) and mass spectrometry. (**A**). Venn diagrams show the total number of identified BNIP3 interacting proteins by mass spectrometry in the human (red) and rat (green) HFrEF LV myocardium. The intersection between the two Venn diagrams (black circle) shows the number of commonly identified BNIP3 interacting proteins in human and rat samples. (**B**,**C**)**.** Heat maps and PCA plots show the relative log_2_-fold expression and the variance in biological samples, respectively, in Sham and HFrEF in the rat LV myocardium of the 516 common identified BNIP3 interacting proteins (left), including those that were differentially expressed in HFrEF vs. Sham, taking a cutoff *p*-value of <0.05 (right). (**D**). Heat maps show the relative log_2_-fold expression in Sham and HFrEF in the rat LV myocardium of some of the important identified BNIP3 interacting proteins that were commonly identified in rat and human HFrEF samples, presented by HFrEF vs. Sham cutoff *p*-value < 0.05 (left), 0.05 < *p* < 0.1 (middle), and *p* > 0.1 (right). (**E**). Western blot showing the expression of sarco/endoplasmic reticulum calcium ATPase 2a (SERCA2a) and the mt-proton/calcium exchanger protein (LETM1) in Sham, ShLuc, and ShBNIP3, * *p* < 0.05 vs. Sham and ^†^ *p* < 0.05 vs. ShLuc; m, monomer; t, trimer. (**F**,**G**). Heat maps of the top *Canonical Pathways* and *Upstream Regulators* that were upregulated/activated or downregulated/inhibited in HFrEF vs. Sham rat samples. The orange and blue color intensities represent the z-score-based extent of upregulation/activation or downregulation/inhibition, respectively. The heat maps were generated in IPA after “Core Analyses” of each of the two-group comparisons (HFrEF vs. Sham), taking a cutoff *p*-value of <0.05 vs. <0.1, which were then compared with each other in IPA’s “Comparison Analyses” function. (**H**). BNIP3 interactome enriched metabolic reactome pathways, by q-value, that were attenuated (blue bars) in HFrEF vs. Sham. Data are presented as percentage of total protein count per reactome pathway. (**I**–**K**). Validation of some of the BNIP3 interacting proteins by Co-IP and western blot in Normal ACM (**I**), Rat LV myocardium, Sham vs. HFrEF (**J**), and human HFrEF LV myocardium (**K**).

Visualization of the total identified BNIP3 interacting proteins in rat LV myocardia, including those that were differentially expressed in HFrEF vs. Sham, are presented in Supplementary Figure S10A–F. Please refer to the figure legend for detail. The canonical pathways that were enriched by *p*-value and the canonical pathways and upstream regulators that were predicted to be upregulated or downregulated by z-score in the two-group comparison, HFrEF vs. Sham, are shown in Supplementary Figure S11A,B. Some of the reactome pathways that were enriched in the rat BNIP3 interactome, including those that were upregulated or downregulated in HFrEF vs. Sham, are shown in Supplementary Figure S11C,E and Supplementary Excel file S2). Visualization of the total identified BNIP3 interacting proteins in human HFrEF LV myocardia, and the enriched reactome pathways, are presented in Supplementary Figure S12A–C and Supplementary Excel file S2. The Venn diagrams in Figure 7A show the total number of identified BNIP3 interacting proteins in rat and human LV myocardia. There were 516 commonly identified BNIP3 interacting proteins between rat and human BNIP3 interactomes (intersection between both Venn diagrams and Supplementary Excel file S6). Heat maps and PCA plots of the 516 common BNIP3 interacting proteins, including those that were differentially expressed in HFrEF vs. Sham, are shown in Figure 7B,C and Figure S13A. The PCA plot in Figure 7B shows a significant variance in the BNIP3 interactome between Sham and HFrEF, which, of course, becomes more pronounced when the differentially expressed proteins are filtered by *p*-value (Figure 7C and Figure S13A). The PPI network of the common proteins between rat and human BNIP3 interactomes (Supplementary Figure S13B) shows that there are four predominant clusters of proteins. The biggest cluster is denoted by the yellow color, representing mt-proteins with a predominant biological function in OXPHOS (TCA cycle and respiratory chain) and metabolism. Metabolic pathways (Supplementary Figure S14C) related to OXPHOS, complex I biogenesis, BCAA catabolism, pyruvate metabolism and regulation, and mt-FA β-oxidation, were among the highest enriched reactome pathways that were identified. Mitochondrial calcium ion transport was also enriched in the BNIP3 interactome. Other mt-proteins involved in mt-translation/elongation (the 28S ribosomal proteins, such as MRPS22, MRPS34, and MRPS36), the mt-dynamic protein optic atrophy 1 (OPA1), mt-SLC25A family of carrier proteins (such as SLC25A11, SlC25A3, and SLC25A4), mt-contact site and cristae organizing system ((MICOS) – TOMM22, TIMM44, and SAMM50), and the mt-Lon protease homolog, LONP1, were also identified to be part of the BNIP3 interactome in both rat and human LV myocardia. LONP1 is an ATP-dependent serine protease that selectively degrades misfolded, unassembled, and/or oxidatively damaged, short-lived regulatory proteins in the mt-matrix. The second largest cluster is denoted by the red color, representing proteins with biological function in translation (the 40S and 60S ribosomal proteins) and eukaryotic translation elongation (such as the eukaryotic translation initiation factors, EIF4A1 and EIF3L). The remaining two clusters, green and blue in color, represent proteins with biological function in metabolism (cytoplasm), autophagy, apoptosis, calcium cycling, and muscle contraction. The reactome pathways (Supplementary Figure S14C) related to metabolism of amino acids and their derivatives, muscle contraction, and the regulation and execution of apoptosis, were also enriched in the BNIP3 interactome.

Heat maps (Figure 7D) show the relative log_2_-fold change in the rat (Sham and HFrEF) LV myocardium for some of the BNIP3 interacting proteins with biological functions involved in OXPHOS, metabolism, calcium cycling and homeostasis, and muscle contraction and translation regulation. The heat maps are presented based on differential expression in HFrEF vs. Sham with *p*-value cutoff of < 0.05, between 0.05 and 0.1, and of > 0.1. The sarcoplasmic/endoplasmic reticulum calcium ATPase isoform 2a (SERCA2a) and the mt-proton/calcium exchanger protein, LETM1, were part of the BNIP3 interactome. LETM1 regulates calcium efflux at the inner mt-membrane, while SERCA2a pumps the cytoplasmic calcium back into the sarcoplasmic reticulum during the relaxation phase of the cardiac cycle. The SERCA2a and LETM1 expression was decreased in ShLuc vs. Sham and increased in ShBNIP3 vs. ShLuc (Figure 7E). More importantly, the LETM1 trimer to monomer ratio downtrended in ShLuc vs. Sham and uptrended in ShBNIP3 vs. ShLuc, suggesting post-translational modification and possibly modulation of LETM1 function.

The heat map in Supplementary Figure S14A shows the canonical pathways that were enriched by −Log_10_ *p*-value in the HFrEF vs. Sham BNIP3 interactome using a cutoff *p*-value of <0.05 vs. <0.1. The heat maps in Figure 7F,G and Figure S14B show the canonical pathways and upstream regulators that were predicted to be upregulated/activated or downregulated/inhibited in the HFrEF vs. Sham BNIP3 interactome using a cutoff *p*-value of <0.05 vs. < 0.1. Reactome pathways that were downregulated in the HFrEF vs. Sham BNIP3 interactome are presented in Figure 7H. The presented data suggest that OXPHOS, mt-FA β-oxidation, BCAA catabolism, pyruvate metabolism, and the TCA cycle were among the highest enriched metabolic pathways that were downregulated in the HFrEF vs. Sham BNIP3 interactome. Moreover, AMPK and PKA signaling, and the upstream regulators PPARA, PPARGC1A, and PPARG, were predicted to be downregulated in the HFrEF vs. Sham BNIP3 interactome. Co-IP followed by western blot (Figure 7I–K) confirmed that VDAC1, SERCA2a, and LETM1 were part of BNIP3 interacting proteins in rat ACM, rat LV myocardium, and human LV myocardium. However, GAPDH was not, and, therefore, was a false discovery.

## 4. Discussion

**Figure 8:**
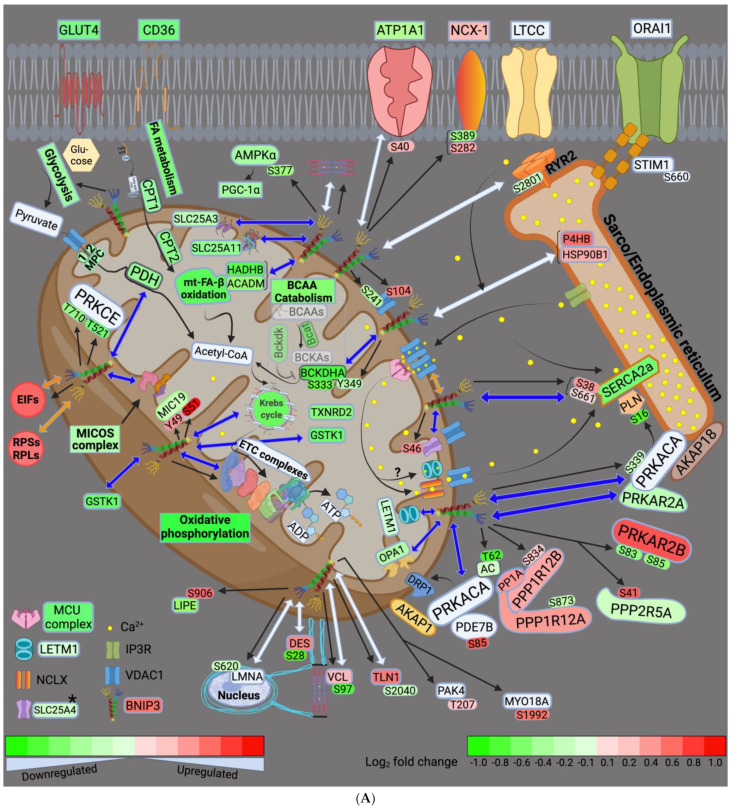

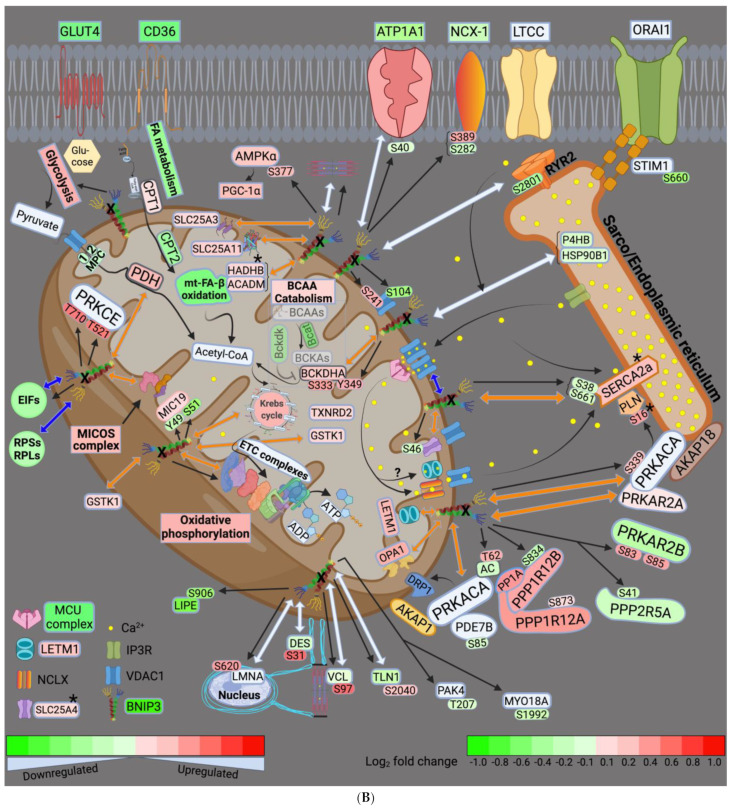
Schematic drawings highlight some of the key findings of the effect of BNIP3 knockdown in the rat pressure overload HFrEF model. These are presented as ShL vs. Sham (**A**) and ShB vs. ShL (**B**). The green and red color intensities show the degree of downregulation and upregulation of activity, respectively, or the log_2_-fold change in protein or phosphosite expression, as noted by the scale bar at the bottom right side of the schematic drawing. The double-headed arrows show the interaction between BNIP3 and its interacting protein. The blue and orange colors show whether there was inhibition/downregulation or activation/upregulation as a result of this interaction. The straight black arrows point to an effect of BNIP3 on protein phosphorylation. The black asterisk denotes changes in protein expression or phosphorylation by western blot only. Abbreviations: GLUT4, glucose transporter member 4; CD36, platelet glycoprotein 4; NCX1, sodium/calcium exchanger 1; LTCC, voltage-dependent L-type calcium channel; ATP1A1, sodium/potassium-transporting ATPase subunit alpha-1; STIM1, stromal interaction molecule 1; ORA1, calcium release-activated calcium channel protein 1; AMPK, 5′-AMP-activated protein kinase; PGC-1α, peroxisome proliferator-activated receptor gamma coactivator 1-alpha; CPT1 and 2, carnitine O-palmitoyltransferase 1 and 2; SLC25a3, mt-phosphate carrier protein; SLC25a11, mt-2-oxoglutarate/malate carrier protein; SLC25a4, ADP/ATP translocase 1; HADHB, mt-trifunctional enzyme subunit beta; FA, fatty acid; Bckdk, mt-3-methyl-2-oxobutanoate dehydrogenase kinase; BCAA, branched-chain amino acid; Bcat, BCAA aminotransferase; BCKAs, branched-chain α-ketoacids; BCKDHA, mt-2-oxoisovalerate dehydrogenase subunit alpha; GSTK1, glutathione S-transferase kappa 1; TXNRD2, mt-thioredoxin reductase 2; BNIP3, BCL2/adenovirus E1B 19 kDa protein-interacting protein 3; VDAC1, voltage-dependent anion channel isoform 1; IP3R, inositol 1,4,5-triphosphate receptor; NCLX, mt-sodium/calcium exchanger; LETM1, mt-proton/calcium exchanger; MCU, mt-calcium uniporter; P4HB, protein disulfide isomerase; HSP90B1, heat shock protein 90 beat member 1; SERCA2a, sarcoplasmic/endoplasmic reticulum calcium ATPase 2a; PLN, phospholamban; RYR2, ryanodine receptor isoform 2; PRKACA, cAMP-dependent protein kinase catalytic subunit alpha; PRKAR2A, cAMP-dependent protein kinase type II-alpha regulatory subunit; PRKAR2B, cAMP-dependent protein kinase type II-beta regulatory subunit; AKAP1 and 18, A-kinase anchor protein 1 and 18; AC, adenylate cyclase; PP1A, serine/threonine-protein phosphatase PP1-alpha catalytic subunit; PPP1R12A and PPP1R12B, PP1 regulatory subunits 12A and 12B; PDE7B, cAMP-specific 3′,5′-cyclic phosphodiesterase 7B; DRP1, dynamin-related protein 1; OPA1, optic atrophy 1; PRKCE, protein kinase C epsilon type; ETC, electron transport chain; MICOS, mt-contact site and cristae organizing system; MIC19, MICOS complex subunit MIC19; EIFs, eukaryotic translation initiation factors; RPSs, 40S ribosomal proteins; RPLs, 60S ribosomal proteins; LIPE, hormone sensitive lipase; DES, desmin; LMNA, lamin A; VCL, vinculin; TLN1, talin-1; PAK4, serine/threonine protein kinase PAK 4; MYO18A, unconventional myosin-XVIIIa.

In this study, we utilized a multiomics approach to globally assess the effect of BNIP3 on myocardial remodeling in HFrEF, and to identify whether these effects of BNIP3 are the result of its selective interaction with neighboring proteins. We showed that BNIP3 largely affects mt-metabolic pathways, and the regulation of ER–mt-calcium cycling and homeostasis. The phosphoproteomics data indicate that BNIP3 modulated three important kinases at the ER–mt interface: the PKA signaling complex, the protein kinase C isoform epsilon (PRKCE), and AMPK. Both AMPK and PRKCE have been shown to regulate mt-metabolic pathways and mt-function, while PKA regulates both mt-function and calcium cycling at the ER–mt interface. The effect of BNIP3 extended beyond metabolism to include the regulation of the MICOS and the mt-SLC25A family of carrier proteins, mt-translation/elongation, cellular translation, ER stress and UPR pathways, oxidative stress, and apoptosis. Moreover, there was interaction of BNIP3 with sarcomeric and cytoskeletal proteins and/or modulation of their function by post-translational phosphorylation. The major effects of BNIP3 are largely summarized in the schematic drawing (Figure 8A,B), and will be summarized and discussed in more detail below.

### 4.1. BNIP3 and Remodeling of the Mt-Proteome

Our data suggest that glycolysis, FA metabolism, and mt-metabolic pathways (pyruvate metabolism, BCAA catabolism, FA β-oxidation, TCA cycle, and OXPHOS) were all downregulated in HFrEF, as previously described [18,25,26,27]. BNIP3 knockdown in HFrEF improved all the aforementioned metabolic pathways except for FA metabolism and mt-FA β-oxidation. The highest BNIP3 interaction was with proteins involved in pyruvate metabolism, the TCA cycle, and OXPHOS. These metabolic pathways had the highest degree of activation/improvement by z-score, with BNIP3 knockdown in HFrEF. Moreover, the phosphorylation of the mt-malate dehydrogenase (MDHM or MDH2) and the mt-cytochrome b-c1 complex subunit 2 (UQCRC2) at serine (Ser)-246 and Ser367, respectively, was enhanced with directional changes towards Sham. BCAA catabolism was improved as well, but to a lesser extent than the aforementioned metabolic pathways. BNIP3 interacted with the mt-enzymes, 2-oxoisovalerate dehydrogenase subunits alpha and beta (BCKDHA and BCKDHB), whose expression was downregulated in HFrEF. These enzymes are known to convert branched-chain alpha-keto acids to acetyl-CoA. BNIP3 knockdown in HFrEF improved their expression, and enhanced p-S333-BCKDHA and p-Y349-BCKDHA, with directional changes towards Sham.

Additional to its direct interaction with and modulation of mt-metabolic pathways, BNIP3 interacted with and its knockdown in HFrEF improved the expression of the mt-carrier proteins SLC25A3, SLC25A11, and attenuated p-S46-SLC25A4, with directional changes towards Sham. These mt-carrier proteins are also known to affect mt-energetics and metabolism by shuffling phosphate, 2-oxoglutarate/malate, and ADP/ATP across the inner mitochondrial membrane, respectively. Recent work has shown that loss of SLC25A3 function induced remodeling of the cardiac mt-protein acylome [28]. The loss of function of SLC25A11 in *C. elegans* was found to control mt-morphology and to directly activate the mt-apoptotic program by sensing the metabolic status of mitochondria [29]. The effect of SLC25A4 phosphorylation at Ser46 is not known. However, mutations in SLC25A4 have been associated with mt-dysfunction-induced muscle weakness and cardiomyopathy [30,31]. The MICOS complex proteins SAMM50, TOMM22, and CHCHD3 (MIC19), and the mt-fusion protein, OPA1, were part of BNIP3 interactome. Their expression was downregulated in HFrEF and improved with BNIP3 knockdown. Interaction of BNIP3 with OPA1 has been previously shown to promote OPA1 degradation and mt-fragmentation [32]. Furthermore, BNIP3 knockdown in HFrEF enhanced p-S94-TOMM70, and attenuated p-Y49-CHCHD3 and p-S51-CHCHD3 with directional changes towards Sham. The expression of TIMM8b and TIMM9 was also improved with BNIP3 knockdown, but our data did not show a direct BNIP3 interaction with them. The aforementioned MICOS complex proteins have been shown to play a role in the transport of mt-precursor proteins through the outer mitochondrial membrane into the IMM and in mt-cristae organization, and in the assembly of the mt-ETC complexes [33,34,35]. BNIP3 was also found to regulate mt-redox homeostasis. Our data suggest that these effects of BNIP3 are mainly related to its interaction with the mt-enzymes glutathione S-transferase kappa1 (GSTK1) and mt-thioredoxin reductase 2 (TXNRD2). Their expression was downregulated in HFrEF and improved with BNIP3 knockdown. The effect of BNIP3 on the mt-superoxide dismutase (SOD2) and the mt-peroxiredoxin-5 (PRDX5, which was identified as part of the BNIP3 interactome) was ambivalent. Their expression was downregulated in both LV and RV proteomic datasets. BNIP3 knockdown improved SOD2 and PRDX5 expression in RV myocardium only, suggesting more of a secondary rather than a direct BNIP3 effect or a false discovery.

Previous work has shown multiple effects of BNIP3 on mitochondria, ranging from the downregulation of the nuclear and mt-encoded ETC complexes to the regulation of mitophagy and apoptosis [5,6,36,37,38,39]. Our findings provide a more in depth understanding into the effect of BNIP3 on the entire mt-proteome and mt-pathobiology, as highlighted above. We show that these effects of BNIP3 are mediated via its direct interaction with specific mt-proteins involved in the above aforementioned pathophysiological processes. Through this direct protein–protein interaction, BNIP3 selectively attenuates the expression of its interacting mt-proteins, and/or alters mt-signaling and their post-translational phosphorylation, rather than leading to a general increase in mt-protease activity and degradation of the mt-proteome, as previously proposed [36]. The effect of BNIP3 on mt-signaling will be discussed in more detail below.

### 4.2. BNIP3 and the Regulation of ER–Mt-Calcium and Ion Homeostasis

Previous work has shown that BNIP3 regulates ER–mt-calcium homeostasis by promoting the dimerization of the VDAC channels [12]. In this study, we show a more fundamental mechanistic insight into how BNIP3 modulates the entire axis of mt-calcium uptake and efflux, and then reuptake into the ER/SR. Our data suggest that BNIP3 interacts with and induces the dimerization of the VDAC1 through a post-translational modification (PTM) mechanism. We found that BNIP3 modulates VDAC1 phosphorylation at Ser104 and Ser241 residues. BNIP3 knockdown in HFrEF attenuated p-S104-VDAC1 and enhanced p-S241-VDAC1, with directional changes towards Sham. Another explanation could be that the increase in BNIP3 expression in HFrEF enhances its interaction and binding to VDAC1, promoting their oligomerization. The Co-IP WB experiment refuted this possibility, as BNIP3 Co-IP was not associated with an increase in VDAC1 pulldown in HFrEF vs. Sham. The phosphorylation of VDAC1 at Ser104 has been shown to stabilize the protein and induce apoptosis [40]. Less is known about the effect of VDAC1 phosphorylation at Ser241. We believe that these effects of BNIP3 on VDAC1 enhance the contact site with the neighboring inositol triphosphate receptors (IP3R) at the ER level, and augment calcium influx into the mt-intermembrane space. This is supported by previous work showing that VDAC1, but not VDAC2 or VDAC3, selectively interacts with the IP3R [41]. This interaction is strengthened by apoptotic stimuli [41], thereby promoting the transfer of apoptotic calcium stimuli into the mitochondria. The mt-calcium uniporter (MCU) complex was downregulated in HFrEF, and BNIP3 did not affect its expression or phosphorylation. Despite the decrease in MCU expression in HFrEF, its permeability to calcium may be enhanced due to the increase in calcium concentration at the mt-intermembrane space [42]. Moreover, previous work has shown that the mt-targeted calcium/calmodulin kinase isoform delta, which is activated in HFrEF, phosphorylates MCU at Ser57 and Ser92 residues and enhances MCU calcium permeability, thereby promoting mt-calcium overload and dysfunction under cardiac stress conditions [43].

The mt-sodium/calcium exchanger (NCLX) is essential for the regulation of mt-calcium homeostasis and efflux, and cellular viability, as previously shown [44,45]. The mt-proton/calcium exchanger protein, LETM1, has also been shown to regulate mt-calcium efflux, mt-tubular networks, and the assembly of mt–ETC supercomplexes [46,47,48,49,50]; thereby playing role in the regulation of mt-calcium homeostasis and OXPHOS. The expression of LETM1 was downregulated in HFrEF. BNIP3 interacted with LETM1 and its knockdown in HFrEF enhanced LETM1 expression. These findings suggest that BNIP3 also regulates mt-calcium efflux and mt-ion homeostasis through its interaction and modulation of LETM1 expression. At the ER level, BNIP3 knockdown enhanced p-S16-PLN, which is the site of phosphorylation, i.e., inhibition, by PKA. BNIP3 interacted with SERCA2a, and its knockdown in HFrEF enhanced SERCA2a expression and attenuated its phosphorylation at Ser38 and Ser661, with directional changes towards Sham. Although BNIP3 was found to interact with the ryanodine receptor isoform 2 (RYR2), BNIP3 did not affect RYR2 expression or its phosphorylation. Interestingly, the phosphoproteomic data suggest that BNIP3 modulated the phosphorylation of the sodium/calcium exchanger 1 (NCX1 or SLC8A1) and the stromal interaction molecule 1 (STIM1). NCX-1 exchanges sodium and calcium ions across the cell membrane based on their intermembrane gradient, while STIM1 mediates the store-operated calcium entry through its interaction with ORAI1 to replenish ER calcium stores upon its depletion. BNIP3 knockdown in HFrEF attenuated p-S282-NCX1 and enhanced p-S389-NCX1, with directional changes towards Sham, whereas it attenuated p-S660-STIM1 vs. ShLuc and Sham. Moreover, BNIP3 interacted with the sodium/potassium-transporting ATPase subunit alpha 1 (ATP1A1), whose expression was downregulated in HFrEF. BNIP3 knockdown in HFrEF attenuated p-S40-ATP1A1, with a directional change towards Sham, whereas its expression was improved in the RV, but not the LV proteomic, datasets. Collectively, the above findings suggest that BNIP3 adversely modulates calcium cycling, and mt-calcium and ion homeostasis.

### 4.3. BNIP3 and PKA, PRKCE, and AMPK Signaling

Our study shows an important role of BNIP3 in regulating cellular signaling that modulates mt-function and calcium cycling in the heart. The cAMP-dependent protein kinase catalytic subunit alpha, PRKACA, and its regulatory subunit, PRKAR2A, were identified as part of the BNIP3 interactome. BNIP3 knockdown improved p-S339-PRKACA, i.e., enhanced its activity, as previously shown [51], and improved PRKAR2A expression, with directional changes towards Sham. In contrary, BNIP3 knockdown in HFrEF attenuated PRKAR2B expression and enhanced its phosphorylation at Ser83 and Ser85 residues, with directional changes towards Sham. Meanwhile, BNIP3 had no effect on PP1A expression and its regulatory subunits PPP1R12A and PPP1R12B, which were upregulated in HFrEF. BNIP3 knockdown enhanced p-S873-PPP1R12A and attenuated p-S834-PPP1R12B, with directional changes towards Sham. Moreover, BNIP3 knockdown attenuated p-S41-PPP2R5A, with a directional change towards Sham. Furthermore, BNIP3 knockdown in HFrEF attenuated p-S85-PDE7B (phosphodiesterase isoform 7B) and enhanced p-T62-AC (adenylate cyclase) with directional changes towards Sham. The BNIP3-mediated improvement in PKA signaling was mainly confined to the ER–mt interface subcellular compartment. This finding could be explained by the existence of multiple cAMP–PKA signaling pathways in distinct subcellular compartments that can be selectively activated, as previously shown [52,53]. This is accomplished by the presence of multiple A-kinase anchor proteins (AKAPs) that anchor PKA to a specific subcellular compartment [54]. It has been shown that AKAP1 and AKAP18 isoform delta anchor PKA to the mitochondria [55] and the SERCA2a-PLN complex [56], respectively. This allows for preferential phosphorylation of a local pool of PKA downstream targets [57]. More importantly, AKAPs possess the ability to gather and coordinate multiple signaling proteins, such as kinases, phosphatases, ACs, and PDEs into one multifunctional transduction complex at different subcellular compartments. This explains the differential activation of the PKA signaling at a specific subcellular compartment depending on the local activity of ACs, PDEs, and phosphatases within that particular multifunctional signaling complex [58]. As highlighted above, BNIP3 interacts with and/or modulates the phosphorylation of the PKA multifunctional complex components at the ER–mt interface subcellular compartment, providing evidence for the enhancement of PKA activity at this subcellular compartment by BNIP3. PKA has been shown to phosphorylate PLN at Ser16, and inhibit its activity, thereby enhancing SERCA2a function [59]. Moreover, the mt-targeted PKA has been shown to protect the mitochondria from toxins and stress [60] by directly phosphorylating and enhancing the function of the ETC complexes I, IV, and V [61,62,63,64], and mt-antioxidant capacity [65]. The mt-targeted PKA also phosphorylates and inhibits the dynamin-related protein 1 (DRP1)-mediated mt-fission and dysfunction [66].

BNIP3 knockdown in HFrEF improved AMPKα signaling, and that of its downstream effector PGC-1α, both of which were downregulated in HFrEF. BNIP3 knockdown enhanced p-S377-AMPKα_2_ and attenuated p-S485/491-AMPKα, with directional changes towards Sham. Although the effect of AMPKα_2_ phosphorylation at Ser377 is not known, its phosphorylation at Ser485/491 exerts an inhibitory effect in the heart, as previously shown [67]. AMPK plays an important role in mt-homeostasis by regulating mt-biogenesis and mt-quality control via mitophagy [68] and metabolism [69,70]. Similarly, PRKCE, which is highly expressed in the heart [71], has been shown to translocate to the mitochondria, where it regulates mt-function and metabolism [72,73,74], and has an overall cardioprotective role [75]. Our findings suggest that PRKCE expression was unchanged; however, its phosphorylation at threonine (Thr)-521 and Thr710 residues was attenuated in HFrEF. BNIP3 knockdown enhanced its phosphorylation at the aforementioned p-sites, with directional changes towards Sham, suggesting an improvement in its activity. The phosphorylation of PRKCE at Thr521 has never been reported. However, its phosphorylation at Thr517 and Thr710 has been shown to enhance its activity [76]. In neurons, previous work has shown a link between cAMP to PRKCE signaling that was mediated via a cAMP-activated guanine exchanger factor Epac [77], but not via PKA. Epac is located upstream of phospholipase C and D, which both are necessary for PRKCE activation and translocation. BNIP3, via the modulation of AC and PDEs at the ER–mt interface, may affect mt-cAMP concentration and mt-cAMP-mediated activation of PKA and PRKCE. The non-targeted metabolomic data showed that 3′,5′ cGMP downtrended, while 3′,5′ cAMP was differentially downregulated, in HFrEF vs. Sham. There was differential upregulation of 3′,5′ cGMP, but not 3′,5′ cAMP, in ShBNIP3 vs. ShLuc. However, we still cannot exclude a local increase in mt-cAMP concentration and signaling, as previous work has shown that intracellular cAMP can be produced by soluble ACs, independent from transmembrane ACs, in response to bicarbonate [78], calcium [79], and change in ATP levels [80]. Another possibility is that the BNIP3-mediated increase in PRKCE phosphorylation and signaling is independent from cAMP signaling.

### 4.4. Other Effects of BNIP3

While the effects of BNIP3 on the regulation of mt-function and metabolism, calcium homeostasis, and signaling at the ER–mt interface, were explicit and coherent, the mechanistic insights by which BNIP3 affects ER stress and UPR, sarcomeric and cytoskeletal proteins, and cellular transcription and translation, are less clear. BNIP3 interacted with the chaperone protein HSP90B1 and the disulfide isomerase protein, P4HB, which were upregulated in HFrEF and are upregulated under conditions of ER-stress-induced UPR and ER-associated degradation (ERAD). The transcription factor X-box-binding protein 1 (XBP1), which regulates the UPR during ER stress, was also activated in HFrEF vs. Sham. BNIP3 knockdown in HFrEF attenuated their expression/activity, thereby suggesting an improvement in ER stress. This is likely a secondary effect of BNIP3-mediated restoration of ER calcium content and calcium homeostasis [12,81], rather than a direct effect of BNIP3 on their transcription and expression. BNIP3 interacted with the cytoskeletal proteins desmin, vinculin, and talin1, which were upregulated in HFrEF vs. Sham. BNIP3 knockdown in HFrEF attenuated their expression and enhanced their phosphorylation at Ser31, Ser97, and Ser2040, respectively. The phosphorylation of desmin at Ser31 by cyclin-dependent kinase-1 has been shown to promote its disassembly [82]. It is unclear whether this effect of BNIP3 on desmin, vinculin, and talin1 is related to BNIP3-mediated changes in signaling and their post-translational phosphorylation, or is secondary to BNIP3-mediated changes in their transcriptional regulation and translation, or a combination of both. Our data suggest that BNIP3 interacted with the 40S and 60S ribosomal proteins, and with the eukaryotic translation initiation factors (EIF), which were upregulated in HFrEF vs. Sham. BNIP3 knockdown in HFrEF attenuated their expression and modulated EIF phosphorylation status. This suggests a role of BNIP3, whether direct or indirect, in the regulation of cellular transcription and translation. The finding that BNIP3 interacted with the protein lamin A, LMNA, and that BNIP3 knockdown in HFrEF enhanced p-S620-LMNA, with a directional change towards Sham, further supports this. LMNA plays an important role in nuclear assembly and chromatin organization. Mutations in LMNA have been shown to cause autosomal, dominant, severe heart disease accounting for 10% of dilated cardiomyopathies [83]. Similarly, mutation or loss of function of desmin, vinculin, and/or talin1 are associated with sudden cardiac death and dilated cardiomyopathies [84,85,86,87]. Moreover, BNIP3 interacted with the cytoskeletal proteins LIM domain-binding protein 3 (LDB3). BNIP3 knockdown in HFrEF attenuated p-S174-LDB3 and attenuated the p-S408-LIMD1 (LIM domain-containing protein 1), with directional changes towards Sham. Our data also suggest a role for BNIP3 in the modulation of sarcomeric proteins and muscle contraction. BNIP3 knockdown attenuated the phosphorylation of the serine/threonine kinase PAK4, and the unconventional myosin-XVIIIa (MYO18A) at threonine 207 and ser1992 residues, respectively, with directional changes towards Sham. BNIP3 interacted with the sarcomeric proteins myomesin-1 (MYOM1) and 2 (MYOM2), and filamin C (FLNC). BNIP3 knockdown in HFrEF attenuated p-S1057-MYOM1 and p-S1184-MYOM2, and enhanced p-S2114-FLNC and p-S2147-FLNC, and p-S192-LMOD2 (leiomodin-2), with directional changes towards Sham. These effects of BNIP3 shows its role in modulating sarcomere organization and function, but the mechanism by which BNIP3 affects the phosphorylation of these sarcomeric proteins remains unclear.

In summary, BNIP3 modulates important pathobiological processes involved in myocardial remodeling and the pathogenesis of HFrEF. We show important molecular mechanisms by which BNIP3 modulates ER–mt signaling, calcium homeostasis, and metabolic remodeling. Our findings also provide suggestions of BNIP3′s role in the regulation of cellular transcription and translation, sarcomere function, and myocardial cytoskeleton. We believe that the potential ability to target BNIP3, and to reverse multiple pathobiological processes involved in the pathogenesis of HFrEF, is a novel finding. This makes BNIP3 an attractive molecular target for the treatment of HFrEF. Our results also provide a framework for future mechanistic investigation and potential therapeutic interventions. We acknowledge the fact that our study has limitations in terms of addressing all the signaling pathways and mechanisms related to BNIP3 that were not elucidated in the bioinformatics analysis, such as BNIP3 interaction with Parkin for the induction of mitophagy. Moreover, the presented work does not differentiate which of the findings are a direct effect of BNIP3 or the consequence of improvement in mitochondrial function and signaling. A more in depth mechanistic work is warranted to address this issue.

## Data Availability

The corresponding has the raw data, which will be available upon request.

## Supplementary Materials

The following supporting information can be downloaded at: https://www.mdpi.com/article/10.3390/cells11091572/s1, Figure S1: Echocardiographic parameters in Sham, ShLuc and ShBNIP3 groups; Figure S2: Visualization of the total identified proteins in LV and RV myocardium proteomic datasets; Figure S3: Visualization of the differentially expressed proteins in ShLuc vs Sham groups in LV and RV myocardium proteomic datasets; Figure S4: Visualization of the differentially expressed mt-proteins in ShLuc vs Sham groups and ShBNIP3 vs ShLuc groups in the LV and RV myocardium proteomic datasets; Figure S5: Visualization of the commonly identified mt-proteins between the LV and RV proteomic datasets; Figure S6: Enriched *Canonical pathways* and *Upstream regulators* in the LV and RV proteomic datasets; Figure S7: Visualization and validation of the differentially expressed metabolites in ShLuc vs Sham in the LV myocardium untargeted metabolomic dataset; Figure S8: Visualization of the differentially expressed phosphosites in ShLuc vs Sham in the LV and RV myocardium p-proteomic datasets; Figure S9: Western blots show the phosphorylation of AMPK at serine 485/491 and threonine 172 residues and downstream targets of PKA as well as the expression of the serine/threonine-protein phosphatase PP1-alpha (PP1a) in Sham, ShLuc and ShBNIP3 groups; Figure S10: BNIP3 interactome in rat LV myocardium by BNIP3 Co-IP and mass spectrometry; Figure S11: BNIP3 interactome enriched *Canonical Pathways*, *Upstream regulators*, and *Reactome pathways* in rat LV myocardium; Figure S12: Visualization of BNIP3 interactome in Human HFrEF LV myocardium; Figure S13: Visualization of the commonly identified BNIP3 interacting proteins in human and rat LV myocardium; Figure S14: Enriched canonical pathways and Upstream regulators of the commonly identified BNIP3 interacting proteins in rat and human LV myocardium; Table S1: Echocardiographic parameters of the animals that were studied in Sham, ShLuc and ShBNIP3 groups; Excel file S1: Proteomic LV and RV datasets; Excel file S2: Reactome pathways; Excel file S3: Untargeted metabolomics dataset; Excel file S4: Phosphoproteomics LV and RV datasets; Excel file S5: List of important phosphoproteins; Excel file S6: Common BNIP3 interactome in rat and Human LV myocardium.
