# Supplementary material for: Multiomics Approach Reveals an Important Role of BNIP3 in Myocardial Remodeling and the Pathogenesis of Heart Failure with Reduced Ejection Fraction
Source: Cells. 2022 May 6;11(9):1572. doi: 10.3390/cells11091572 (PMC9105187; doi:10.3390/cells11091572)
Supplement: Supplementary file 1 [file cells-11-01572-s001.zip › Supplementary Table and Figures_20220402.pdf]

Supplemental Table and Figures

Table S1. Echocardiographic parameters of the animals that were studied in Sham, ShLuc and ShBNIP3 groups.

	Wk3-post-AAB			Wk8-HFrEF development			Wk4-post-Rx			Two-way ANOVA		
**Para-meters**	**Sham n=3**	**ShLuc n=3**	**ShBNIP3 n=3**	**Sham n=3**	**ShLuc n=3**	**ShBNIP3 n=3**	**Sham n=3**	**ShLuc n=3**	**ShBNIP3 n=3**	**Interac-tion**	**Row Factor**	**Column Factor**
**HW/BW (mg/g)**							2.35 ± 0.13	4.67 ± 0.16*	4.76 ± 0.51*	NA	NA	NA
**LVW/BW (mg/g)**							1.66 ± 0.14	3.17 ± 0.04*	3.16 ± 0.27*	NA	NA	NA
**RVW/BW (mg/g)**							0.41 ± 0.03	1.00 ± 0.20*	0.80 ± 0.03*†	NA	NA	NA
**BW (g)**	322 ± 8	318 ± 16	324 ± 15	496 ± 10	478 ± 34	512 ± 62	559 ± 8	503 ± 61	546 ± 62	NS	****	NS
**IVSd (cm)**	0.17 ± 0.001	0.26 ± 0.01*	0.28 ± 0.02*	0.19 ± 0.01	0.27 ± 0.01*	0.28 ± 0.03*	0.19 ± 0.01	0.26 ± 0.02	0.28 ± 0.02	NS	NS	****
**LVPWd (cm)**	0.17 ± 0.01	0.26 ± 0.01*	0.29 ± 0.01*	0.21 ± 0.02	0.28 ± 0.01*	0.29 ± 0.02*	0.2 ± 0.004	0.28 ± 0.04*	0.29 ± 0.01*	NS	NS	****
**LVIDd (cm)**	0.68 ± 0.06	0.58 ± 0.04*	0.57 ± 0.06*	0.69 ± 0.03	0.83 ± 0.03*	0.85 ± 0.04*	0.70 ± 0.01	0.94 ± 0.06*	0.86 ± 0.06*†	****	****	P=0.001
**LVIDs (%)**	0.26 ± 0.04	0.14 ± 0.03*	0.14 ± 0.03*	0.28 ± 0.02	0.47 ± 0.01*	0.49 ± 0.01*	0.27 ± 0.002	0.62 ± 0.07*	0.42 ± 0.07*†	****	****	****
**LVFS (%)**	62 ± 3	78 ± 1*	76 ± 4*	60 ± 2	42 ± 4*	42 ± 4*	62 ± 2	34 ± 3*	52 ± 5*†	****	****	****
**LVEDV (µl)**	472 ± 36	352 ± 45*	333 ± 12*	561 ± 71	901 ± 116*	901 ± 101*	557 ± 36	1068 ± 122*	724 ± 43*†	****	****	****
**LVESV (µl)**	90 ± 15	40 ± 9	37 ± 10	102 ± 23	432 ± 63*	400 ± 36*	104 ± 9	659 ± 81*	220 ± 72*†	****	****	****
**LVEF (%)**	81 ± 2	89 ± 1.4*	89 ± 3*	82 ± 2	52 ± 1*	56 ± 2*	81 ± 1	38 ± 2*	71 ± 6*†	****	****	****

Data are presented as mean ± standard deviation. Statistical analysis was performed in Prism software version 9.1.0. One-way ANOVA with Benjamini, Krieger and Yekuteli correction method for multiple comparisons was used to assess statistical significance for the following parameters: HW/BW, LVW/BW, and RVW/BW. The remaining echocardiographic parameters were analyzed by two-way ANONA with Benjamini, Krieger and Yekuteli correction method for multiple comparisons of cell means regardless of rows and columns to assess for statistical significance and interaction. The rows represent the time points at which echocardiographic parameters were obtained (wk3 post-AAB, wk8-time of HF development and treatment (Rx), and wk4-post Rx). The columns represent the studied groups: Sham, ShLuc, and ShBNIP3. A p-value of < 0.05 was considered significant.

Abbreviations: NA: not applicable, NS: not significant, IVSd: Interventricular septal diameter, LVPWd: left ventricular (LV) posterior wall diameter, LVIDd: LV end-diastolic diameter, LVIDs: LV end-systolic diameter, LVEDV: LV end-diastolic volume, LVESV: LV end-systolic volume, LVEF: LV ejection fraction, BW: body weight, HW: heart weight, LVW: LV weight, and RVW: right ventricular weight.

*P < 0.05 vs Sham
†P < 0.05 vs ShLuc
****P < 0.0001

Figures and figure legends

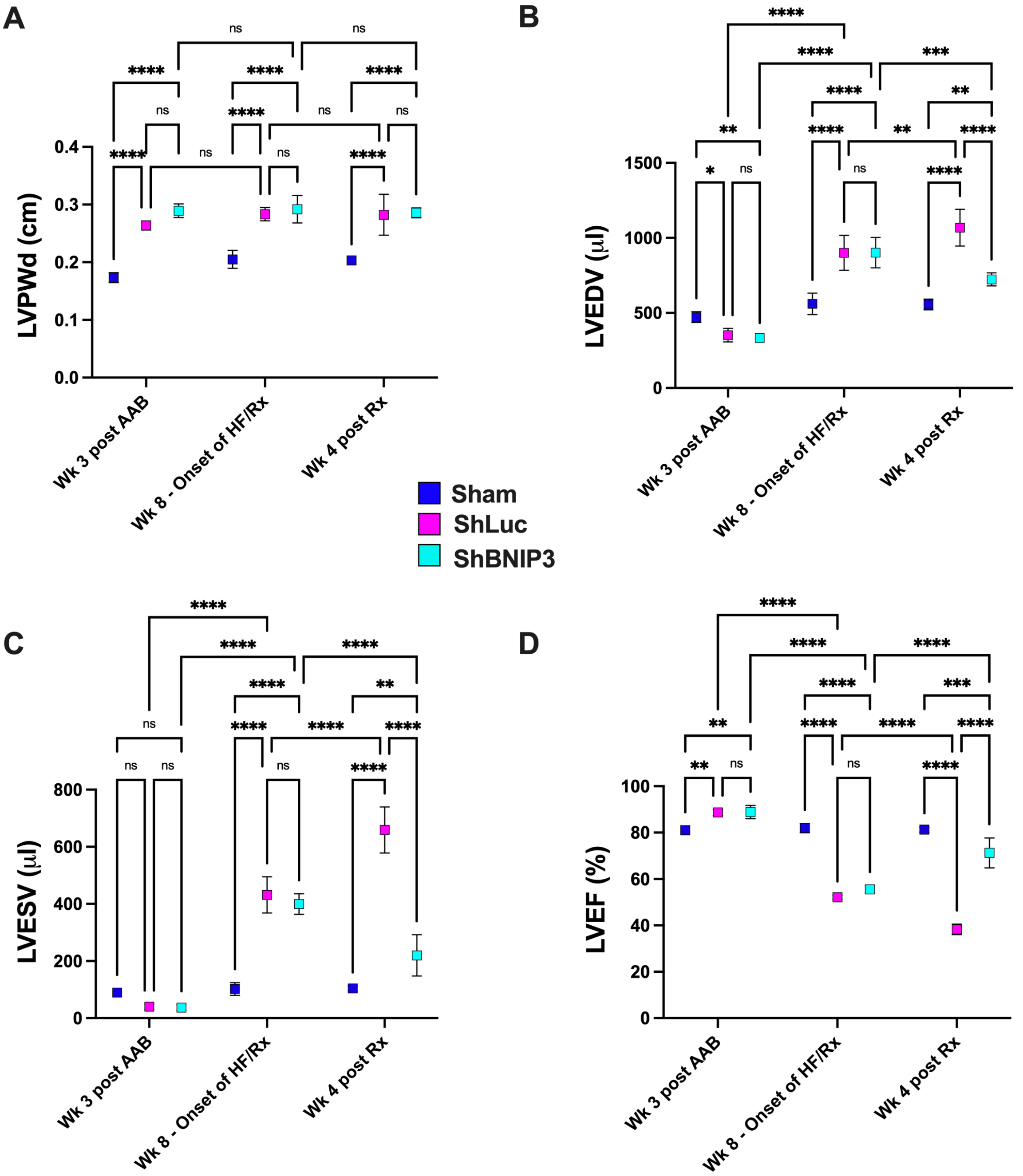

Figure S1. Echocardiographic parameters in Sham, ShLuc and ShBNIP3 groups. LV posterior wall thickness (LVPWd) (**A**), LV end-diastolic (LVEDV) and end-systolic volumes (LVESV) (**B-C**), and LV ejection fraction (LVEF) (**D**) are shown at the time points of: concentric remodeling (week (wk)3 post-AAB), HF development and gene delivery (wk8 post-AAB) and wk4 post treatment (Rx) (wk12 post-AAB). $^{*}P < 0.05$.

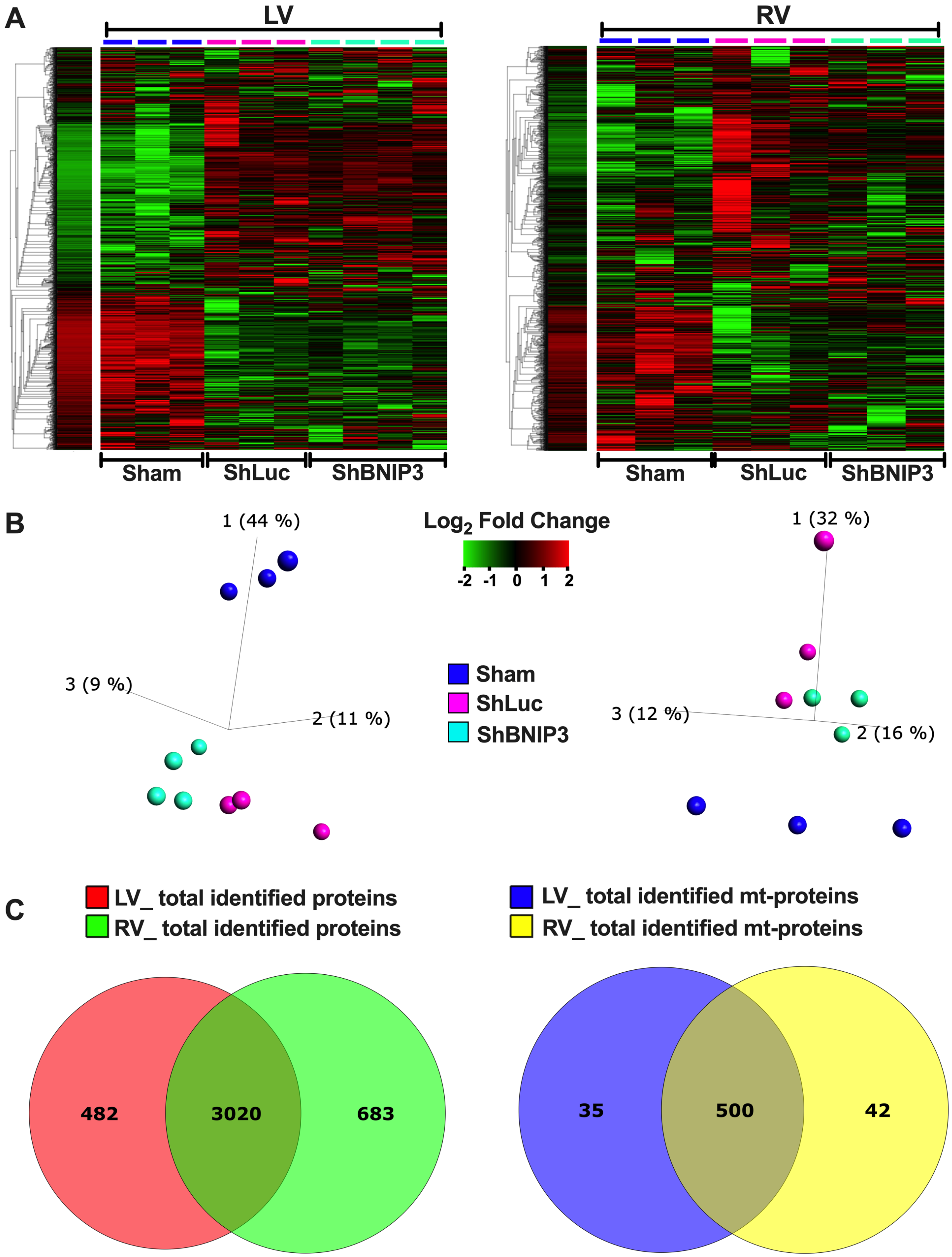

Figure S2. Visualization of the total identified proteins in LV and RV myocardium proteomic datasets. A-B. Heat maps and PCA plots show the relative $\log_2$ fold expression and the variance in biological samples, respectively, in Sham, ShLuc and ShBNIP3 groups of the total identified proteins in LV (left), and RV (right) shotgun proteomic datasets. **C.** Venn diagrams show the number of total identified proteins (left) and mitochondrial (mt)-proteins in LV and RV shotgun proteomic datasets. The intersection between the Venn diagrams show the number of proteins and mt-proteins that were commonly identified in LV and RV shotgun proteomic datasets.

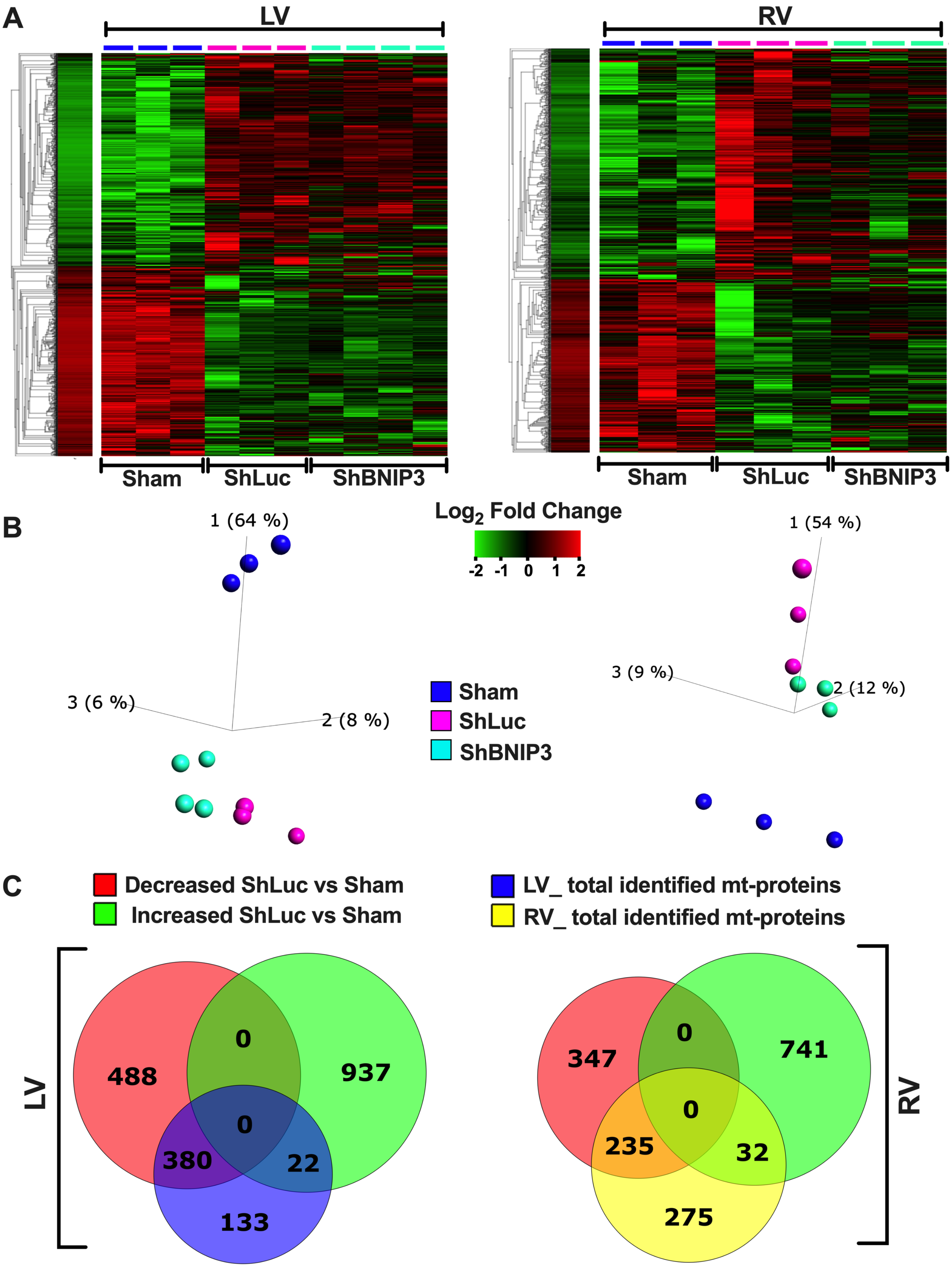

A
LV
RV
Sham
ShLuc
ShBNIP3
B
Log2 Fold Change
-2 -1 0 1 2
1 (64 %)
3 (6 %)
2 (8 %)
1 (54 %)
3 (9 %)
2 (12 %)
Sham
ShLuc
ShBNIP3
C
Decreased ShLuc vs Sham
Increased ShLuc vs Sham
LV_ total identified mt-proteins
RV_ total identified mt-proteins
LV
488
0
937
0
380
22
133
RV
347
0
741
0
235
32
275

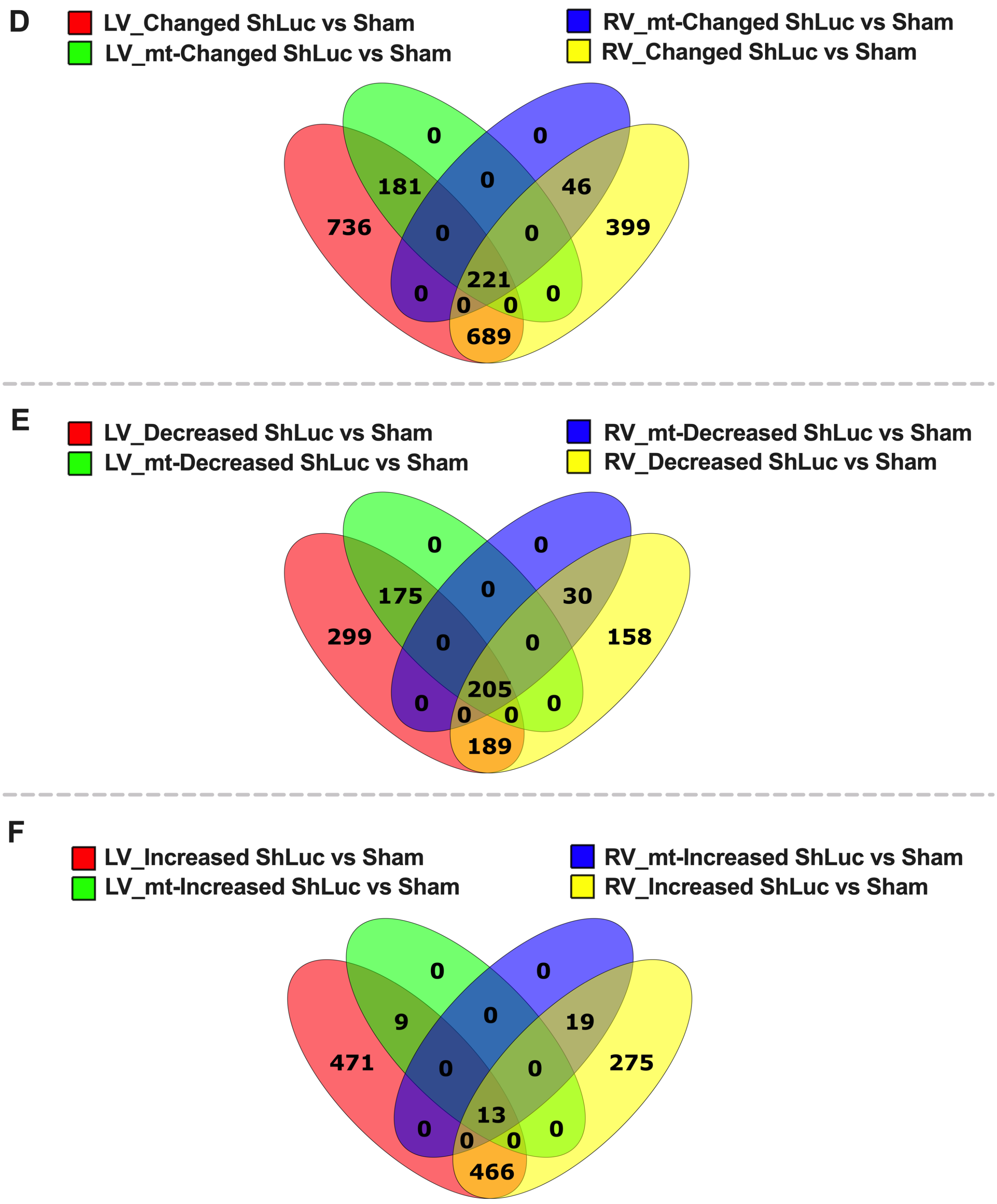

Figure S3. Visualization of the differentially expressed proteins in ShLuc vs Sham groups in LV and RV myocardium proteomic datasets. A-B. Heat maps and PCA plots show the relative $\log_2$ fold expression and the variance in biological samples, respectively, in Sham, ShLuc and ShBNIP3 groups of the differentially expressed proteins in ShLuc vs Sham groups in LV (left), and RV (right) shotgun proteomic datasets. **C.** Venn diagrams show the number of differentially expressed proteins that were downregulated (red) or upregulated (green) in ShLuc vs Sham in LV (left) and RV (right) shotgun proteomic datasets, and how many of these were mitochondrial (mt)-proteins (intersection of blue or yellow with red and green diagrams). **D-F.** Venn diagrams show the total number of differentially expressed proteins and mt-proteins in ShLuc vs Sham (**D**), and how many of these were downregulated (**E**), or upregulated (**F**) in ShLuc vs Sham in the LV (red and green) and RV (yellow and blue) proteomic datasets, respectively. They also show the number of the commonly identified proteins (intersection of red with yellow) and mt-proteins (intersection of green with blue) that were differentially expressed, downregulated or upregulated, between the LV and RV proteomic datasets.

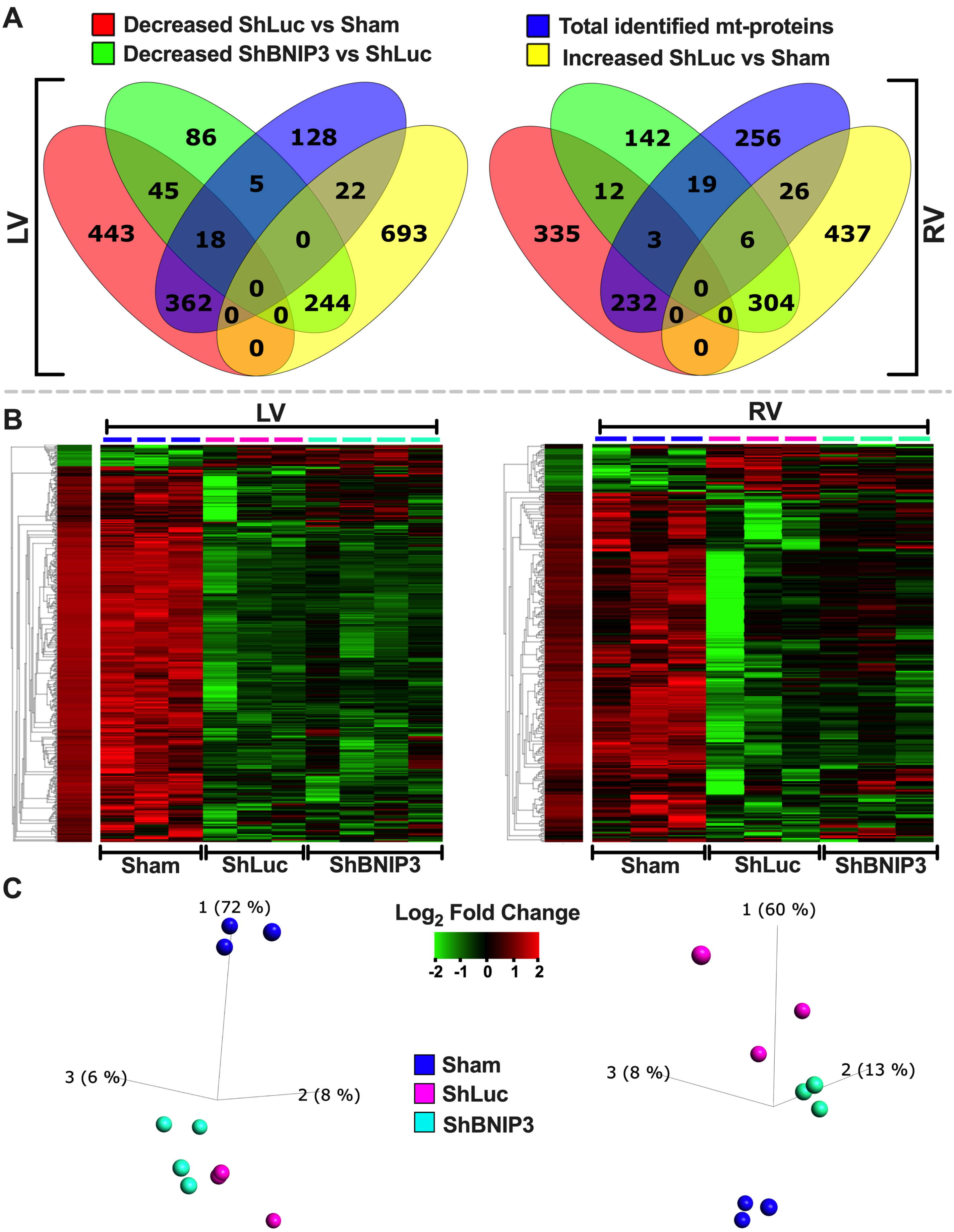
A
Decreased ShLuc vs Sham
Decreased ShBNIP3 vs ShLuc
Total identified mt-proteins
Increased ShLuc vs Sham
LV
86
128
45
5
22
443
18
0
693
0
362
0
0
244
0
142
256
12
19
26
335
3
6
437
0
232
0
0
304
0
RV
B
LV
RV
Sham
ShLuc
ShBNIP3
Sham
ShLuc
ShBNIP3
C
1 (72 %)
3 (6 %)
2 (8 %)
Log2 Fold Change
-2
-1
0
1
2
Sham
ShLuc
ShBNIP3
1 (60 %)
3 (8 %)
2 (13 %)

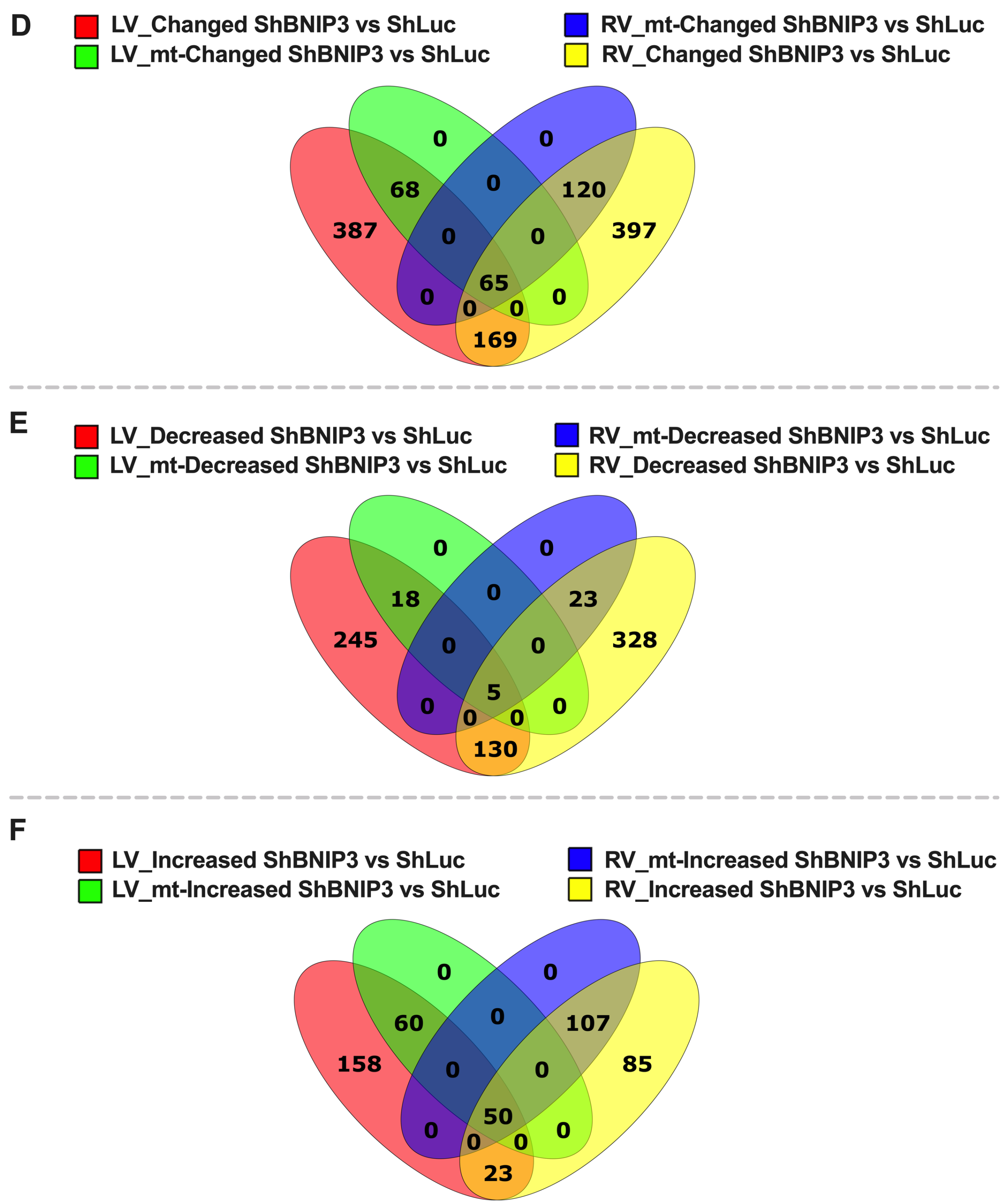

Figure S4. Visualization of the differentially expressed mt-proteins in ShLuc vs Sham groups and ShBNIP3 vs ShLuc groups in the LV and RV myocardium proteomic datasets. A. Venn diagrams show the number of differentially expressed proteins that were downregulated (red) or upregulated (yellow) in ShLuc vs Sham in LV (left) and RV (right) shotgun proteomic datasets, and how many of these were mt-proteins (intersection of blue with red and yellow diagrams). They also show the number of differentially expressed proteins that were downregulated in ShBNIP3 vs ShLuc (green), and how many of these were mt-proteins (intersection of blue with green diagrams). **B-C.** Heat maps and PCA plots show the relative log_2 fold expression and the variance in biological samples, respectively, in Sham, ShLuc and ShBNIP3 groups of the differentially expressed mt-proteins in ShLuc vs Sham in LV (left), and RV (right) shotgun proteomic datasets. **D-F.** Venn diagrams show the total number of differentially expressed proteins and mt-proteins in ShBNIP3 vs ShLuc (**D**), and how many of these were downregulated (**E**), or upregulated (**F**) in ShBNIP3 vs ShLuc in the LV (red and green) and RV (yellow and blue) proteomic datasets, respectively. They also show the number of the commonly identified proteins (intersection of red with yellow) and mt-proteins (intersection of green with blue) that were differentially expressed, downregulated or upregulated, between the LV and RV proteomic datasets.

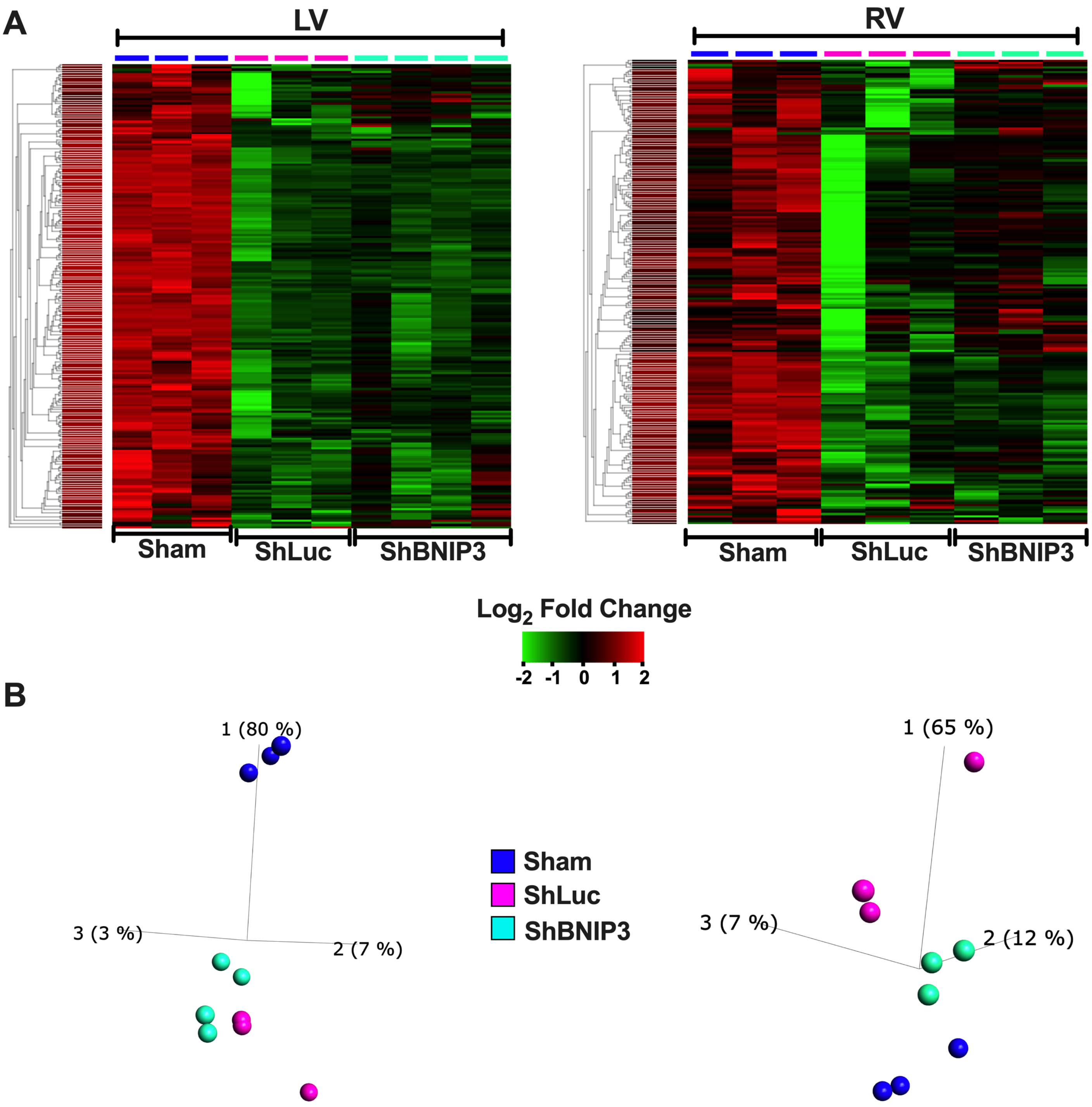
A
LV
RV
Sham
ShLuc
ShBNIP3
Log2 Fold Change
-2 -1 0 1 2
B
1 (80 %)
3 (3 %)
2 (7 %)
1 (65 %)
3 (7 %)
2 (12 %)
Sham
ShLuc
ShBNIP3

C

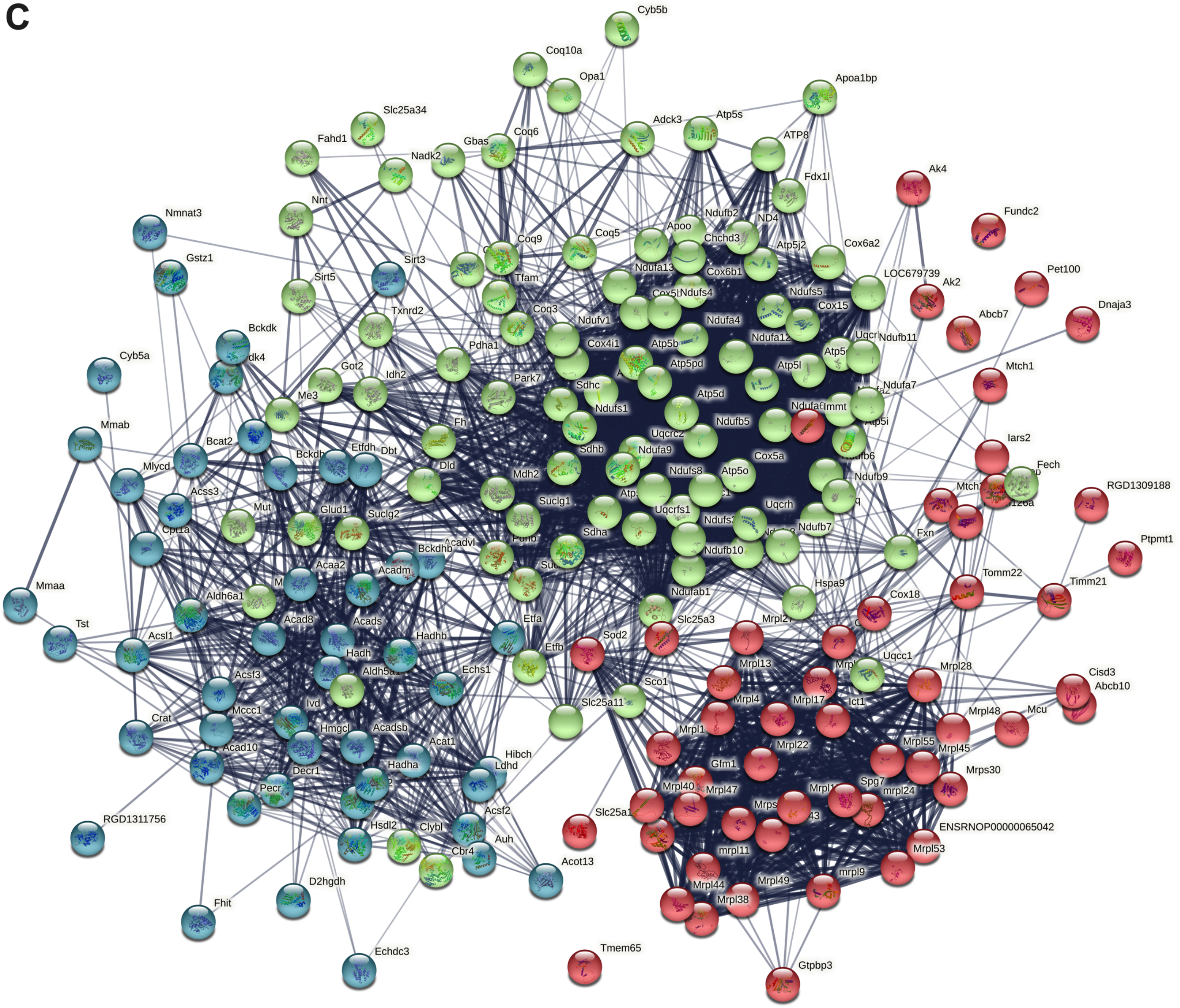

Cyb5b
Coq10a
Opa1
Apoa1bp
Slc25a34
Fahd1
Gbas
Coq6
Adck3
Atp5s
ATP8
Nadk2
Fdx1l
Ak4
Fundc2
Nnt
Nmnat3
Ndufb2
ND4
Apoo
Chchd3
Atp5j2
Cox6a2
Coq9
Coq5
Gstz1
Sirt3
Ndufa13
Cox6b1
LOC679739
Pet100
Sirt5
Tfam
Ndufs4
Ndufs5
Ak2
Dnaja3
Txnrd2
Coq3
Cox15
Abcb7
Bckdk
Ndufv1
Ndufa4
Ndufa12
Ndufb11
Cyb5a
Pdha1
Cox4i1
Atp5b
Atp5
Got2
Idh2
Atp5pd
Atp5l
Mtch1
Park7
Sdhc
Ndufa7
Me3
Atp5d
Immt
Mmab
Ndufs1
Atp5i
Bcat2
Fh
Uqcrc2
Ndufb5
Iars2
Etfdh
Dbt
Sdhb
Ndufa9
Cox5a
Mlycd
Dld
Fech
Mdh2
Ndufs8
Atp5o
Ndufb9
RGD1309188
Acss3
Suclg1
Uqcrh
Mut
Glud1
Suclg2
Uqcrfs1
Ndufb7
Fxn
Ptpmt1
Mmaa
Acadvl
Bckdhb
Sdha
Ndufb10
Acaa2
Acadm
Hspa9
Tomm22
Timm21
Aldh6a1
Ndufab1
Cox18
Tst
Acad8
Acads
Etfa
Slc25a3
Acsl1
Hadhb
Sod2
Hadh
Etfb
Uqcc1
Mrpl13
Mrpl28
Cisd3
Abcb10
Acsf3
Echs1
Sco1
Ivd
Mrpl4
Mrpl17
Ict1
Mccc1
Slc25a11
Mrpl48
Mcu
Crat
Hmgcl
Acadsb
Mrpl1
Acad10
Acat1
Mrpl22
Mrpl55
Mrpl45
Hibch
Ldhd
Decr1
Hadha
Gfm1
Mrps30
Pecr
Mrpl40
Mrpl47
Spg7
mrpl24
Acsf2
RGD1311756
Hsdl2
Clybl
Auh
ENSRNOP00000065042
Cbr4
mrpl11
Mrpl53
Acot13
D2hgdh
Mrpl44
Mrpl49
mrpl9
Fhit
Mrpl38
Tmem65
Echdc3
Gtpbp3

D

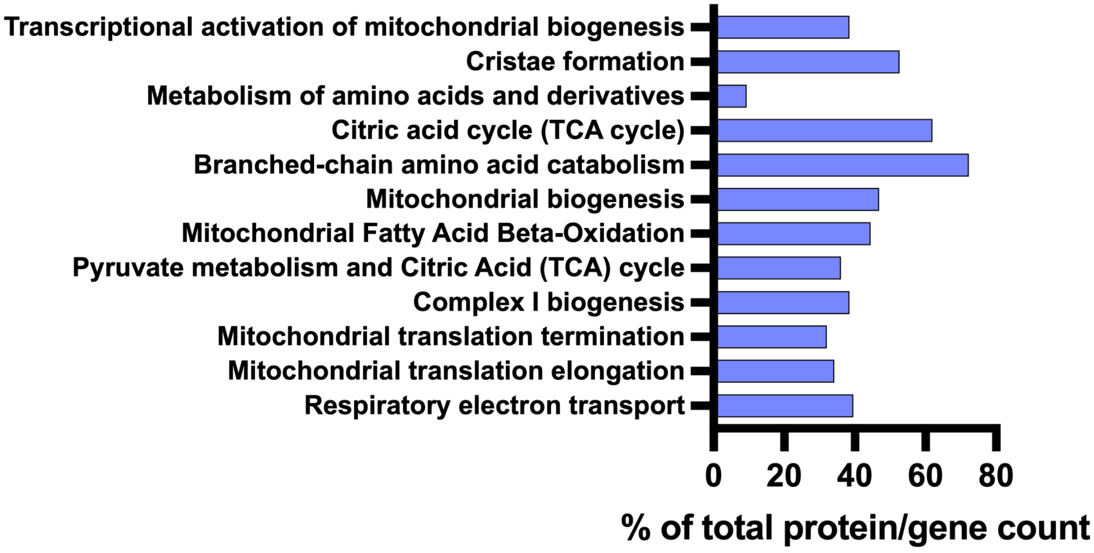

Transcriptional activation of mitochondrial biogenesis
Cristae formation
Metabolism of amino acids and derivatives
Citric acid cycle (TCA cycle)
Branched-chain amino acid catabolism
Mitochondrial biogenesis
Mitochondrial Fatty Acid Beta-Oxidation
Pyruvate metabolism and Citric Acid (TCA) cycle
Complex I biogenesis
Mitochondrial translation termination
Mitochondrial translation elongation
Respiratory electron transport
0
20
40
60
80
% of total protein/gene count

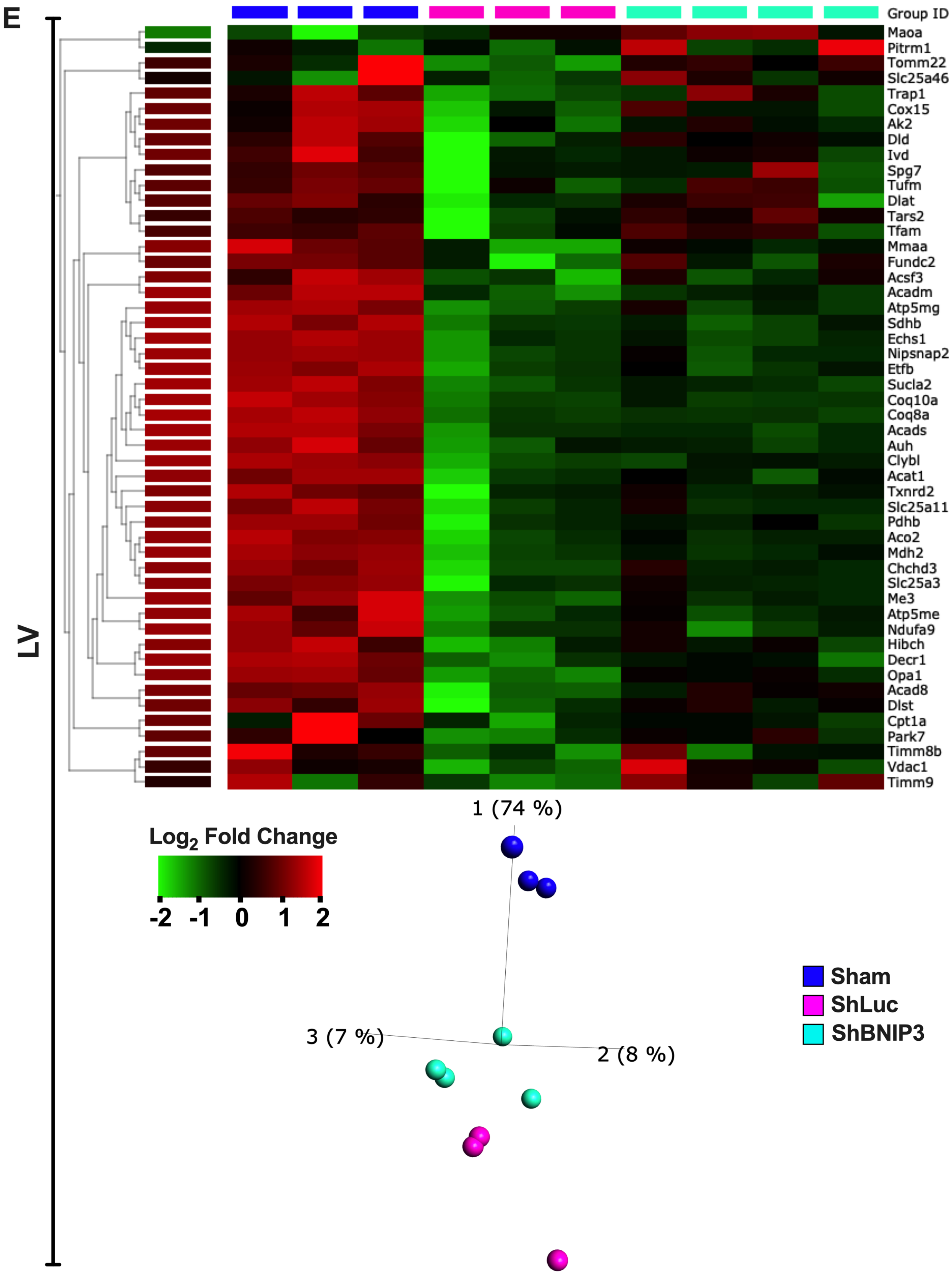
E
LV
Group ID
Maoa
Pitrm1
Tomm22
Slc25a46
Trap1
Cox15
Ak2
Dld
Ivd
Spg7
Tufm
Dlat
Tars2
Tfam
Mmaa
Fundc2
Acsf3
Acadm
Atp5mg
Sdhb
Echs1
Nipsnap2
Etfb
Sucla2
Coq10a
Coq8a
Acads
Auh
Clybl
Acat1
Txnrd2
Slc25a11
Pdhb
Aco2
Mdh2
Chchd3
Slc25a3
Me3
Atp5me
Ndufa9
Hibch
Decr1
Opa1
Acad8
Dlst
Cpt1a
Park7
Timm8b
Vdac1
Timm9
Log2 Fold Change
-2 -1 0 1 2
1 (74 %)
3 (7 %)
2 (8 %)
Sham
ShLuc
ShBNIP3

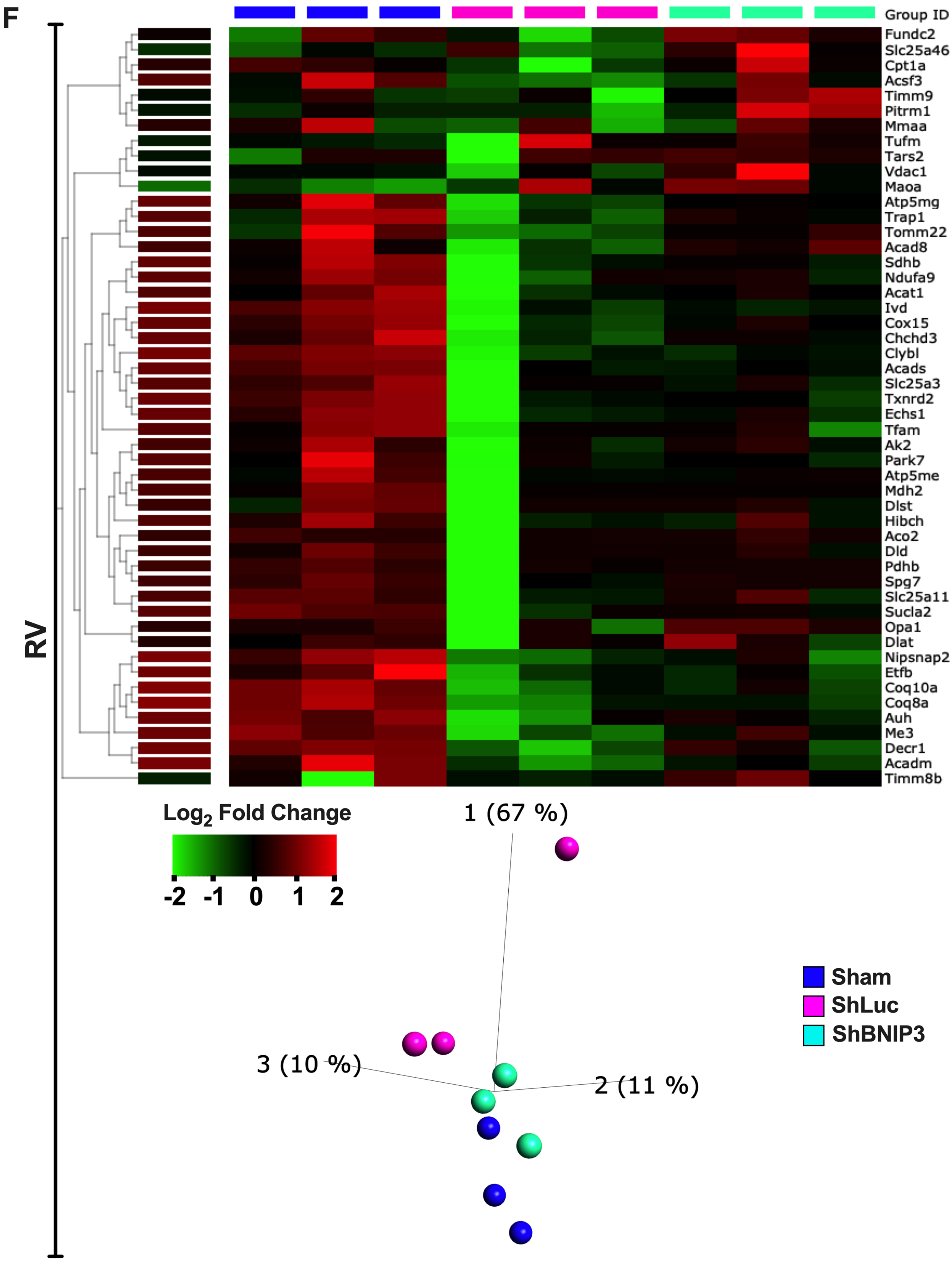
F
RV
Group ID
Fundc2
Slc25a46
Cpt1a
Acsf3
Timm9
Pitrm1
Mmaa
Tufm
Tars2
Vdac1
Maoa
Atp5mg
Trap1
Tomm22
Acad8
Sdhb
Ndufa9
Acat1
Ivd
Cox15
Chchd3
Clybl
Acads
Slc25a3
Txnrd2
Echs1
Tfam
Ak2
Park7
Atp5me
Mdh2
Dlst
Hibch
Aco2
Dld
Pdhb
Spg7
Slc25a11
Sucla2
Opa1
Dlat
Nipsnap2
Etfb
Coq10a
Coq8a
Auh
Me3
Decr1
Acadm
Timm8b
Log2 Fold Change
-2 -1 0 1 2
1 (67 %)
2 (11 %)
3 (10 %)
Sham
ShLuc
ShBNIP3

G

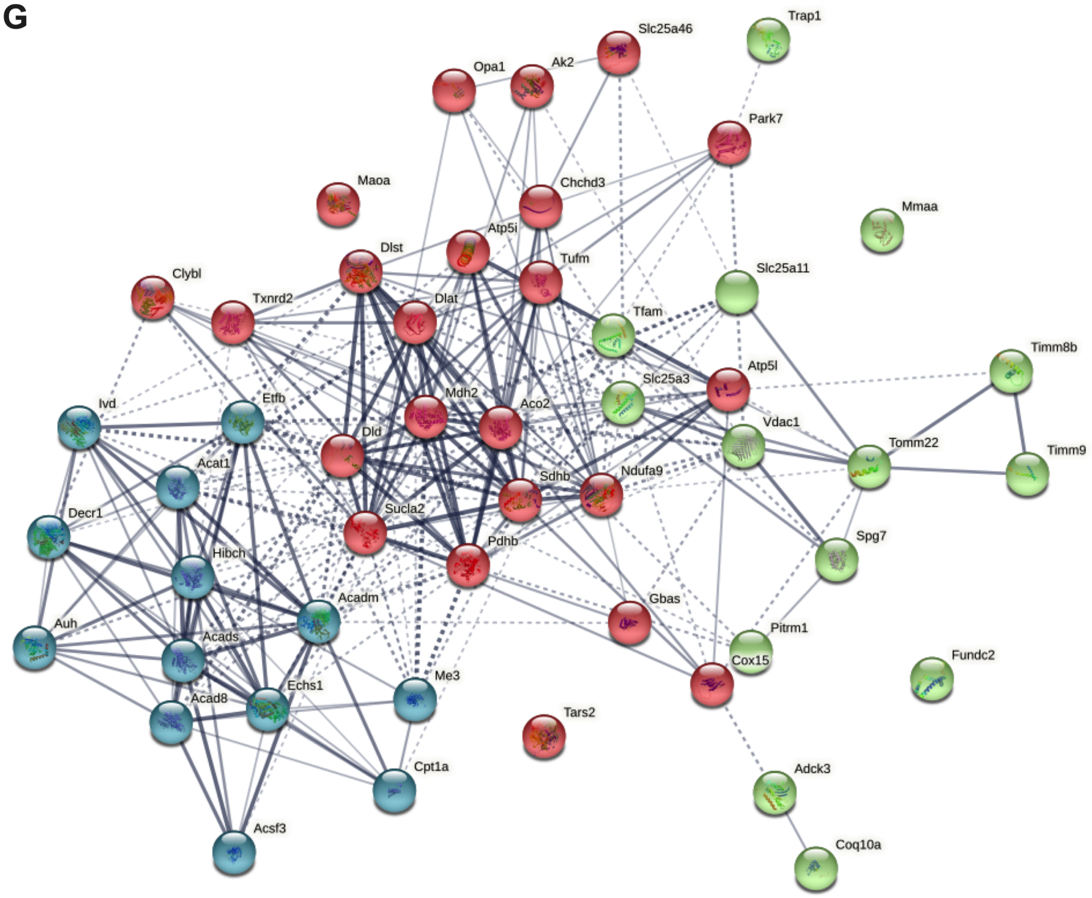

H

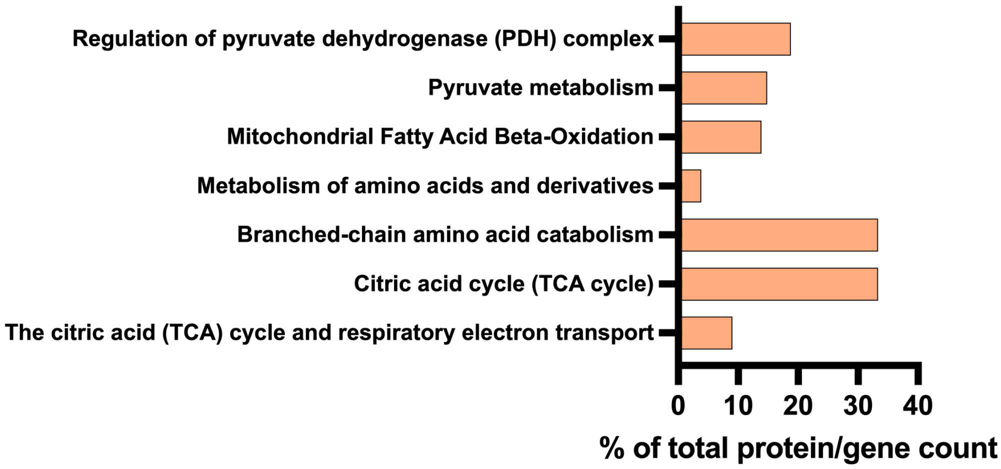

Figure S5. Visualization of the commonly identified mt-proteins between the LV and RV proteomic datasets. Heat maps and PCA plots show the relative $\log_2$ fold expression and the variance in biological samples, respectively, in Sham, ShLuc and ShBNIP3 groups of the commonly identified mt-proteins between LV and RV shotgun proteomic datasets that were downregulated in ShLuc vs Sham (**A-B**) and those that were upregulated in ShBNIP3 vs ShLuc (**E-F**). Protein-protein interaction (PPI) network of the commonly identified mt-proteins between LV and RV shotgun proteomic datasets that were downregulated in ShLuc vs Sham (**C**) and those that were upregulated in ShBNIP3 vs ShLuc (**G**), PPI enrichment p-value of 1.0e-16. Protein network was generated using STRING database. Each node represents a protein, whilst edges (lines) represent protein-protein associations based on physical and physiological interaction by confidence. The thicker the line is, the higher is the confidence. Each color represents a cluster of proteins with specific function. Bar graphs show some of the mt-reactome pathways that were downregulated in ShLuc vs Sham (**D**) and upregulated in ShBNIP3 vs ShLuc (**H**) by q-value. Data are presented as percentage of total protein count per reactome pathway.

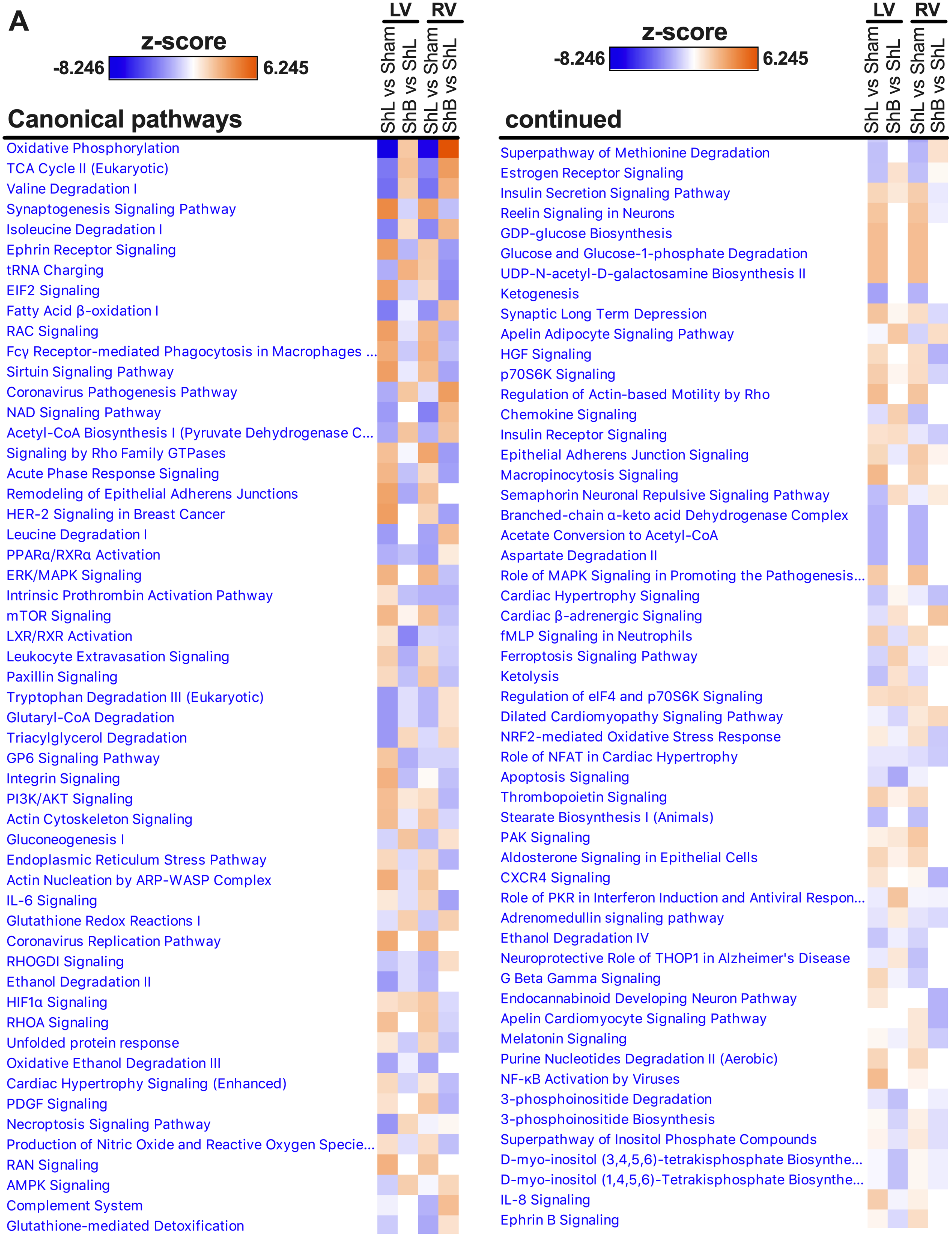
A
z-score
-8.246
6.245
LV
RV
ShL vs Sham
ShB vs ShL
ShL vs Sham
ShB vs ShL
Canonical pathways
Oxidative Phosphorylation
TCA Cycle II (Eukaryotic)
Valine Degradation I
Synaptogenesis Signaling Pathway
Isoleucine Degradation I
Ephrin Receptor Signaling
tRNA Charging
EIF2 Signaling
Fatty Acid β-oxidation I
RAC Signaling
Fcγ Receptor-mediated Phagocytosis in Macrophages ...
Sirtuin Signaling Pathway
Coronavirus Pathogenesis Pathway
NAD Signaling Pathway
Acetyl-CoA Biosynthesis I (Pyruvate Dehydrogenase C...
Signaling by Rho Family GTPases
Acute Phase Response Signaling
Remodeling of Epithelial Adherens Junctions
HER-2 Signaling in Breast Cancer
Leucine Degradation I
PPARα/RXRα Activation
ERK/MAPK Signaling
Intrinsic Prothrombin Activation Pathway
mTOR Signaling
LXR/RXR Activation
Leukocyte Extravasation Signaling
Paxillin Signaling
Tryptophan Degradation III (Eukaryotic)
Glutaryl-CoA Degradation
Triacylglycerol Degradation
GP6 Signaling Pathway
Integrin Signaling
PI3K/AKT Signaling
Actin Cytoskeleton Signaling
Gluconeogenesis I
Endoplasmic Reticulum Stress Pathway
Actin Nucleation by ARP-WASP Complex
IL-6 Signaling
Glutathione Redox Reactions I
Coronavirus Replication Pathway
RHOGDI Signaling
Ethanol Degradation II
HIF1α Signaling
RHOA Signaling
Unfolded protein response
Oxidative Ethanol Degradation III
Cardiac Hypertrophy Signaling (Enhanced)
PDGF Signaling
Necroptosis Signaling Pathway
Production of Nitric Oxide and Reactive Oxygen Specie...
RAN Signaling
AMPK Signaling
Complement System
Glutathione-mediated Detoxification
z-score
-8.246
6.245
LV
RV
ShL vs Sham
ShB vs ShL
ShL vs Sham
ShB vs ShL
continued
Superpathway of Methionine Degradation
Estrogen Receptor Signaling
Insulin Secretion Signaling Pathway
Reelin Signaling in Neurons
GDP-glucose Biosynthesis
Glucose and Glucose-1-phosphate Degradation
UDP-N-acetyl-D-galactosamine Biosynthesis II
Ketogenesis
Synaptic Long Term Depression
Apelin Adipocyte Signaling Pathway
HGF Signaling
p70S6K Signaling
Regulation of Actin-based Motility by Rho
Chemokine Signaling
Insulin Receptor Signaling
Epithelial Adherens Junction Signaling
Macropinocytosis Signaling
Semaphorin Neuronal Repulsive Signaling Pathway
Branched-chain α-keto acid Dehydrogenase Complex
Acetate Conversion to Acetyl-CoA
Aspartate Degradation II
Role of MAPK Signaling in Promoting the Pathogenesis...
Cardiac Hypertrophy Signaling
Cardiac β-adrenergic Signaling
fMLP Signaling in Neutrophils
Ferroptosis Signaling Pathway
Ketolysis
Regulation of eIF4 and p70S6K Signaling
Dilated Cardiomyopathy Signaling Pathway
NRF2-mediated Oxidative Stress Response
Role of NFAT in Cardiac Hypertrophy
Apoptosis Signaling
Thrombopoietin Signaling
Stearate Biosynthesis I (Animals)
PAK Signaling
Aldosterone Signaling in Epithelial Cells
CXCR4 Signaling
Role of PKR in Interferon Induction and Antiviral Respon...
Adrenomedullin signaling pathway
Ethanol Degradation IV
Neuroprotective Role of THOP1 in Alzheimer's Disease
G Beta Gamma Signaling
Endocannabinoid Developing Neuron Pathway
Apelin Cardiomyocyte Signaling Pathway
Melatonin Signaling
Purine Nucleotides Degradation II (Aerobic)
NF-κB Activation by Viruses
3-phosphoinositide Degradation
3-phosphoinositide Biosynthesis
Superpathway of Inositol Phosphate Compounds
D-myo-inositol (3,4,5,6)-tetrakisphosphate Biosynthe...
D-myo-inositol (1,4,5,6)-Tetrakisphosphate Biosynthe...
IL-8 Signaling
Ephrin B Signaling

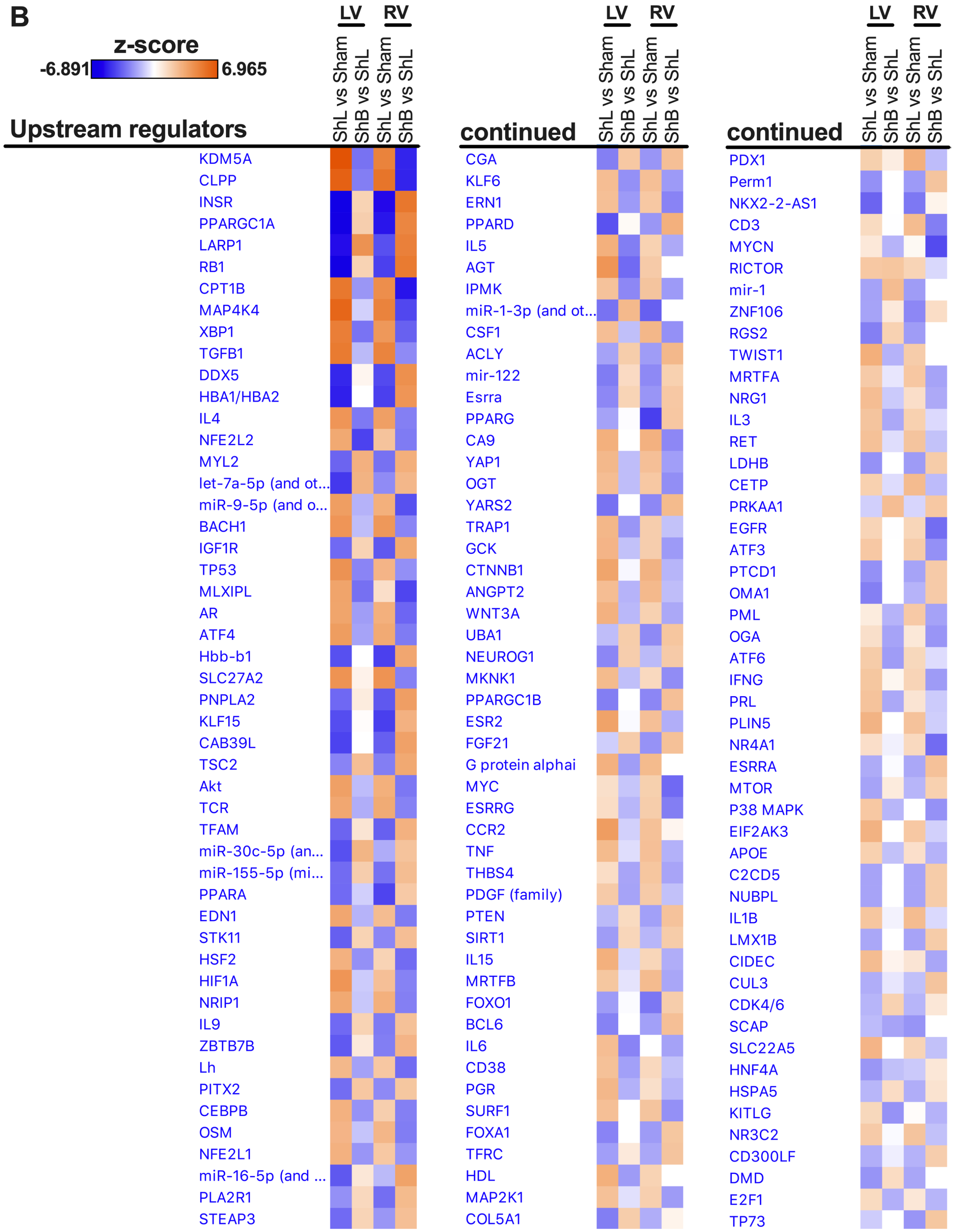
B
z-score
-6.891
6.965
LV
RV
ShL vs Sham
ShB vs ShL
ShL vs Sham
ShB vs ShL
Upstream regulators
KDM5A
CLPP
INSR
PPARGC1A
LARP1
RB1
CPT1B
MAP4K4
XBP1
TGFB1
DDX5
HBA1/HBA2
IL4
NFE2L2
MYL2
let-7a-5p (and ot...
miR-9-5p (and o...
BACH1
IGF1R
TP53
MLXIPL
AR
ATF4
Hbb-b1
SLC27A2
PNPLA2
KLF15
CAB39L
TSC2
Akt
TCR
TFAM
miR-30c-5p (an...
miR-155-5p (mi...
PPARA
EDN1
STK11
HSF2
HIF1A
NRIP1
IL9
ZBTB7B
Lh
PITX2
CEBPB
OSM
NFE2L1
miR-16-5p (and ...
PLA2R1
STEAP3
continued
CGA
KLF6
ERN1
PPARD
IL5
AGT
IPMK
miR-1-3p (and ot...
CSF1
ACLY
mir-122
Esrra
PPARG
CA9
YAP1
OGT
YARS2
TRAP1
GCK
CTNNB1
ANGPT2
WNT3A
UBA1
NEUROG1
MKNK1
PPARGC1B
ESR2
FGF21
G protein alphai
MYC
ESRRG
CCR2
TNF
THBS4
PDGF (family)
PTEN
SIRT1
IL15
MRTFB
FOXO1
BCL6
IL6
CD38
PGR
SURF1
FOXA1
TFRC
HDL
MAP2K1
COL5A1
continued
PDX1
Perm1
NKX2-2-AS1
CD3
MYCN
RICTOR
mir-1
ZNF106
RGS2
TWIST1
MRTFA
NRG1
IL3
RET
LDHB
CETP
PRKAA1
EGFR
ATF3
PTCD1
OMA1
PML
OGA
ATF6
IFNG
PRL
PLIN5
NR4A1
ESRRA
MTOR
P38 MAPK
EIF2AK3
APOE
C2CD5
NUBPL
IL1B
LMX1B
CIDEC
CUL3
CDK4/6
SCAP
SLC22A5
HNF4A
HSPA5
KITLG
NR3C2
CD300LF
DMD
E2F1
TP73

C

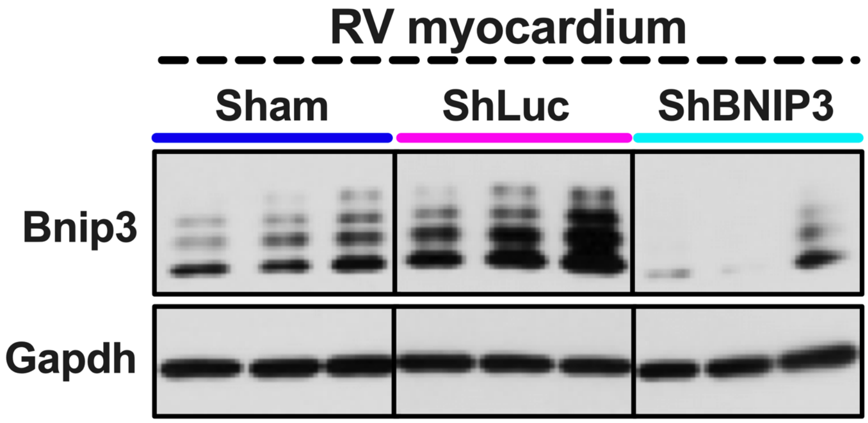

Figure S6. Enriched *Canonical pathways* and *Upstream regulators* in the LV and RV proteomic datasets. Heat maps of the *Canonical pathways* (**A**) and *Upstream regulators* (**B**) that were up-regulated/activated or down-regulated/inhibited in ShLuc vs Sham and ShBNIP3 vs ShLuc in LV and RV proteomic datasets. The orange and blue color intensities represent the z-score-based extent of up-regulation/activation or down-regulation/inhibition, respectively. The heat maps were generated in IPA after 'Core Analyses' of each of the two-group comparisons, i.e. ShLuc vs Sham and ShBNIP3 vs ShLuc, which were then compared with each other in IPA's 'Comparison Analyses' function. **C.** Immunoblotting show BNIP3 expression in RV myocardium of the Sham and HFrEF, ShLuc vs ShBNIP3, groups.

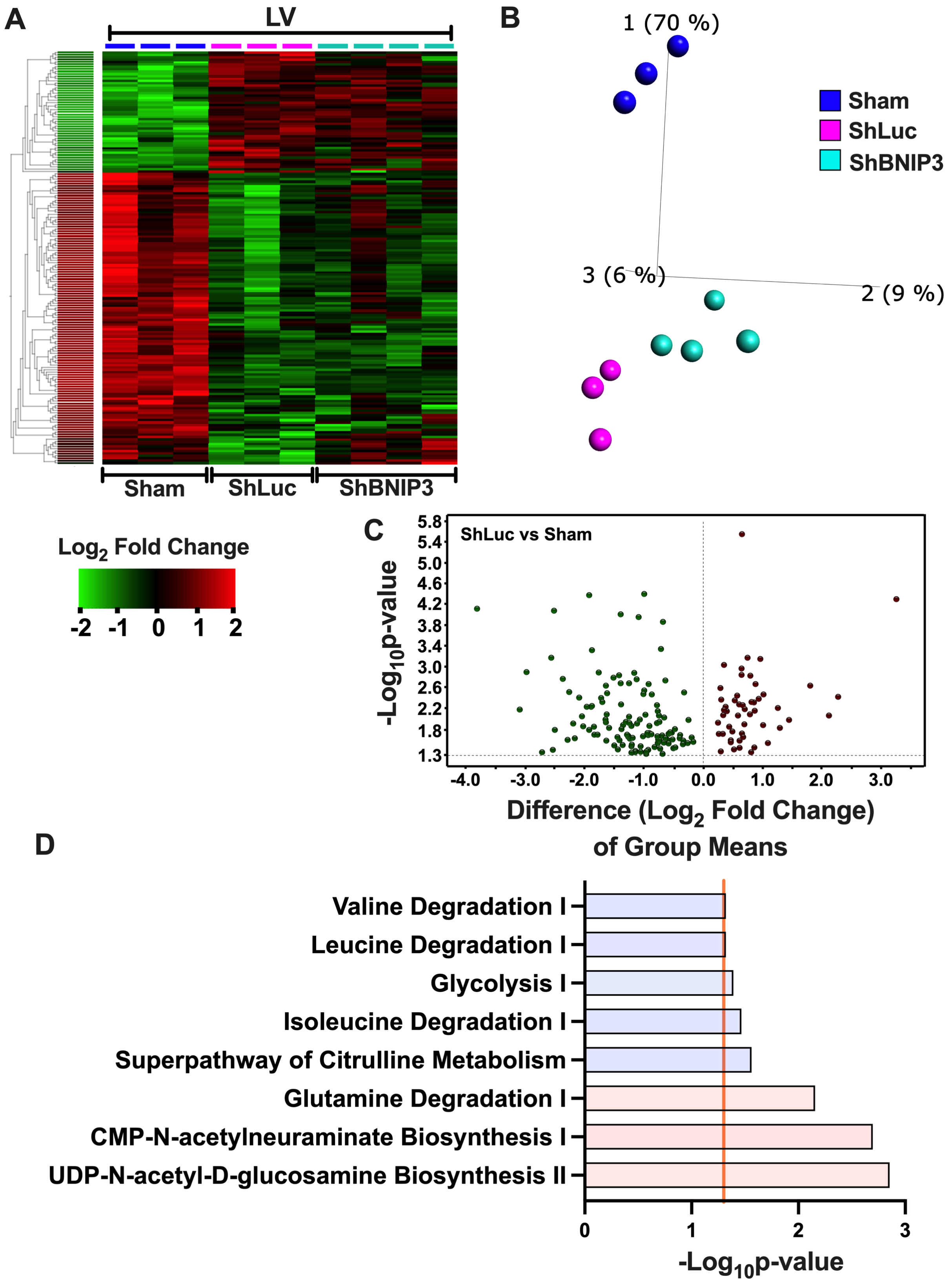

Figure S7. Visualization and validation of the differentially expressed metabolites in ShLuc vs Sham in the LV myocardium untargeted metabolomic dataset. A-B. Heat maps and PCA plots show the relative $\log_2$ fold expression and the variance in biological samples, respectively, in Sham, ShLuc and ShBNIP3 groups of the differentially abundant metabolites in ShLuc vs Sham. **C.** Volcano plot shows the Log_2 fold change of group means for the metabolites whose abundance increased (red) or decreased (green) in ShLuc vs Sham; taking a cutoff $-\text{Log}_{10}$ p-value of 1.3 ($P < 0.05$). **D.** Metabolic pathways that were predicted to be enhanced (orange bar) or attenuated (blue bar) in ShLuc vs Sham; taking a cutoff $-\text{Log}_{10}$ p-value of 1.3 (orange line).

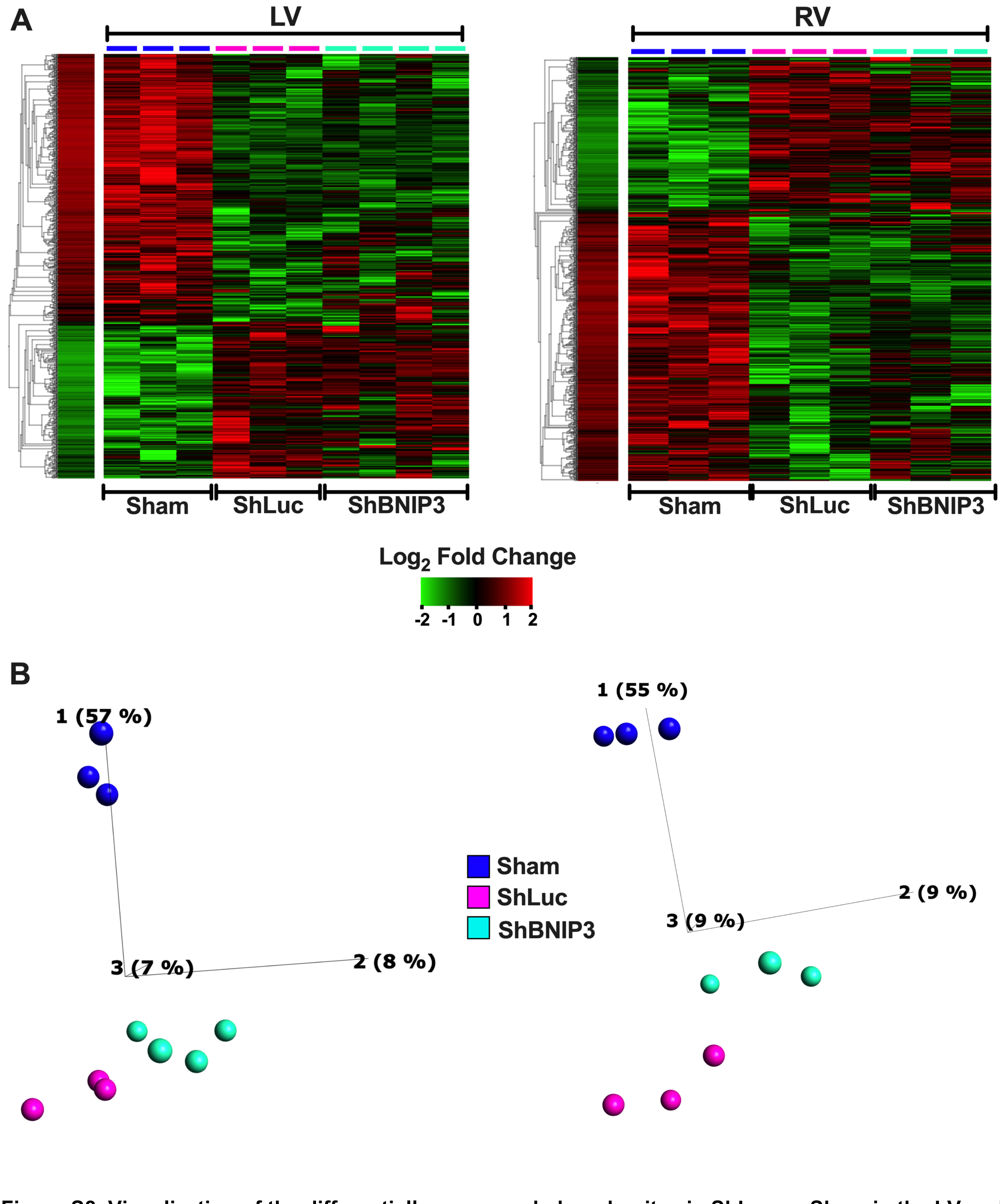

Figure S8. Visualization of the differentially expressed phosphosites in ShLuc vs Sham in the LV and RV myocardium p-proteomic datasets. A-B. Heat maps and PCA plots show the relative $\log_2$ fold expression and the variance in biological samples, respectively, in Sham, ShLuc and ShBNIP3 groups of the differentially expressed phosphosites in ShLuc vs Sham in LV (left), and RV (right) shotgun phosphoproteomic datasets.

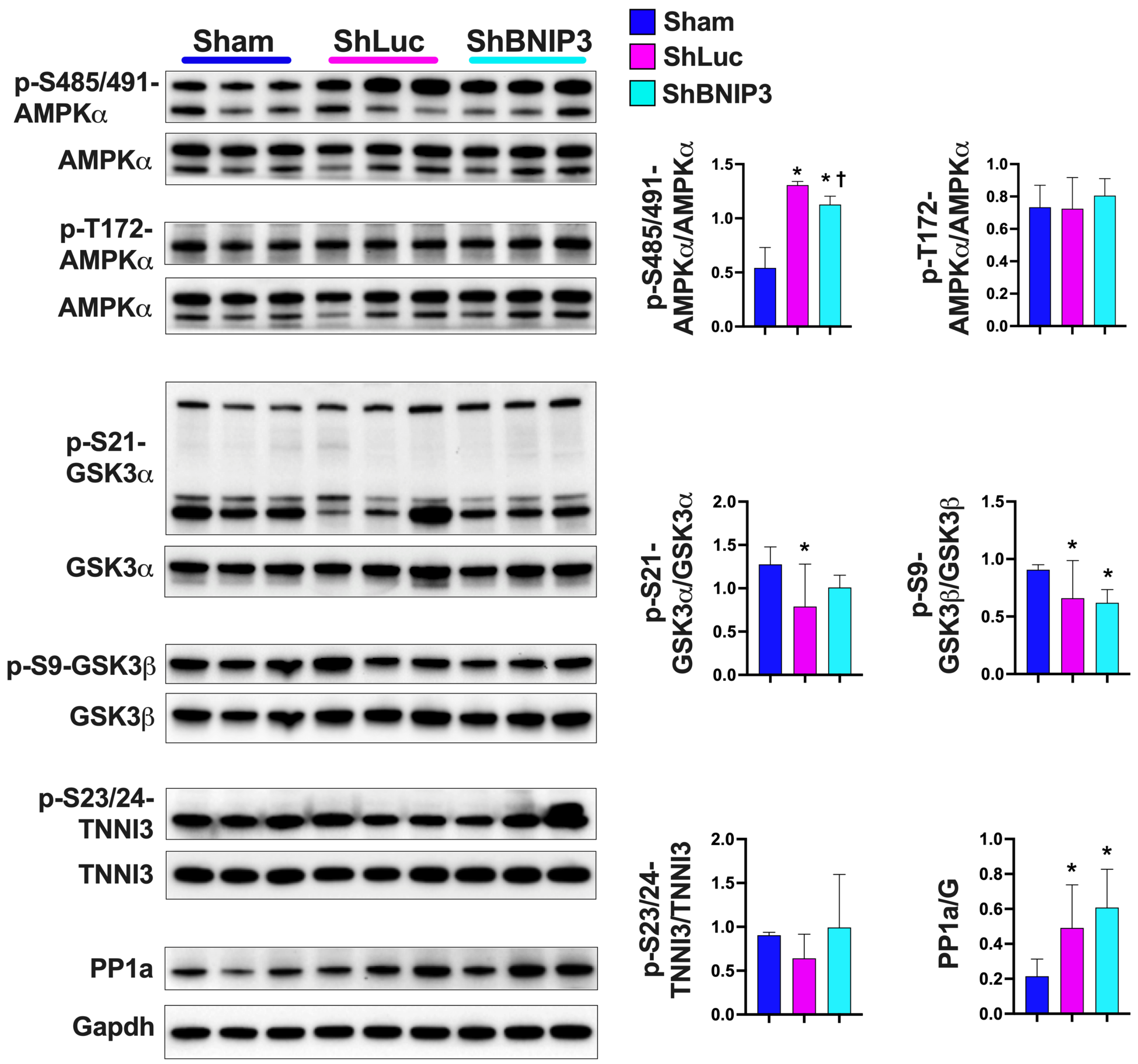

Figure S9. Western blots show the phosphorylation of AMPK at serine 485/491 and threonine 172 residues and downstream targets of PKA as well as the expression of the serine/threonine-protein phosphatase PP1-alpha (PP1a) in Sham, ShLuc and ShBNIP3 groups. Bar graphs are presented as mean ± SD, *P < 0.05 vs Sham, and †P < 0.05 vs. ShLuc.

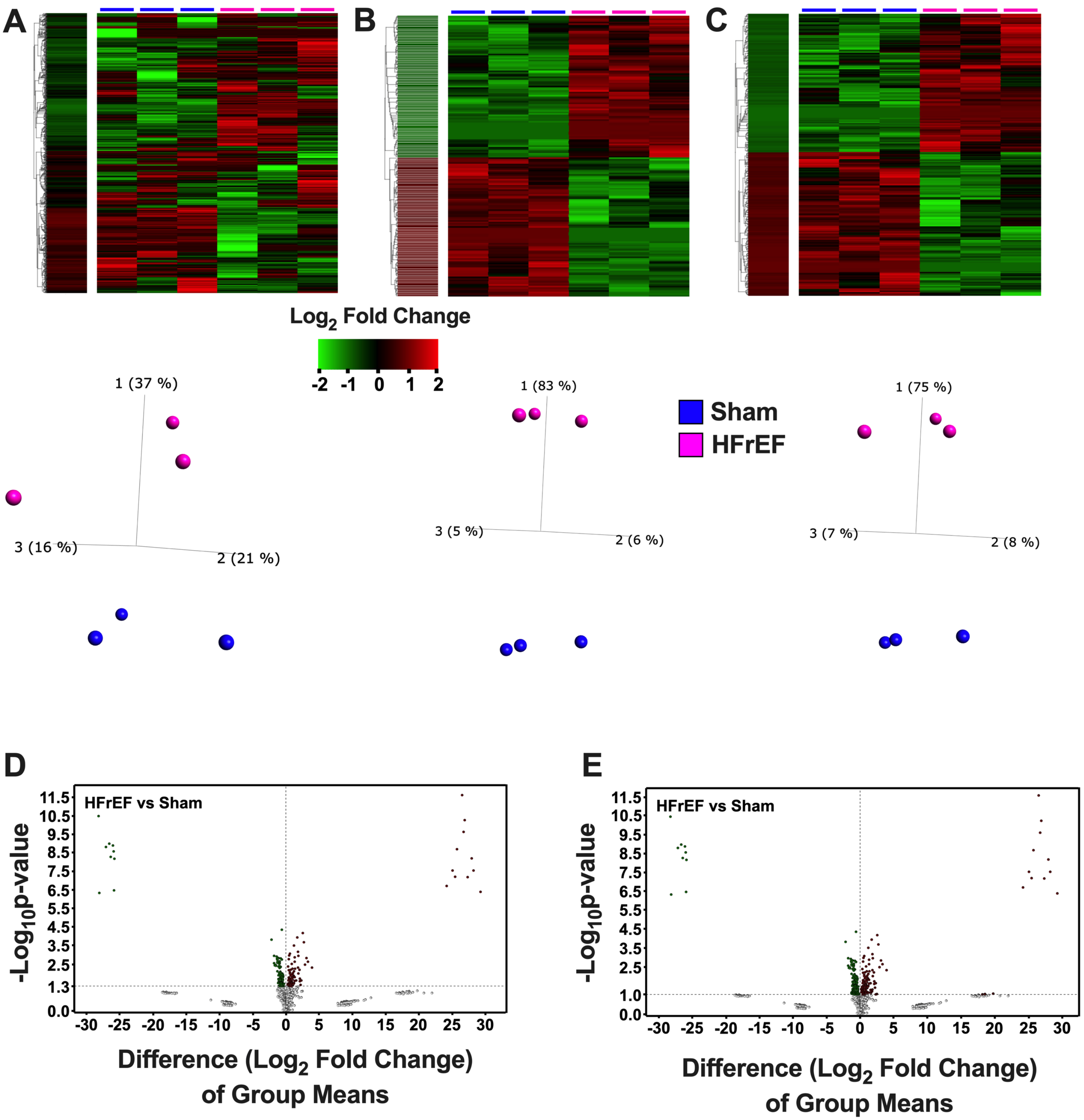
A
B
C
Log2 Fold Change
-2 -1 0 1 2
1 (37 %)
2 (21 %)
3 (16 %)
1 (83 %)
2 (6 %)
3 (5 %)
1 (75 %)
2 (8 %)
3 (7 %)
Sham
HFrEF
D
E
HFrEF vs Sham
-Log10p-value
Difference (Log2 Fold Change) of Group Means
HFrEF vs Sham
-Log10p-value
Difference (Log2 Fold Change) of Group Means

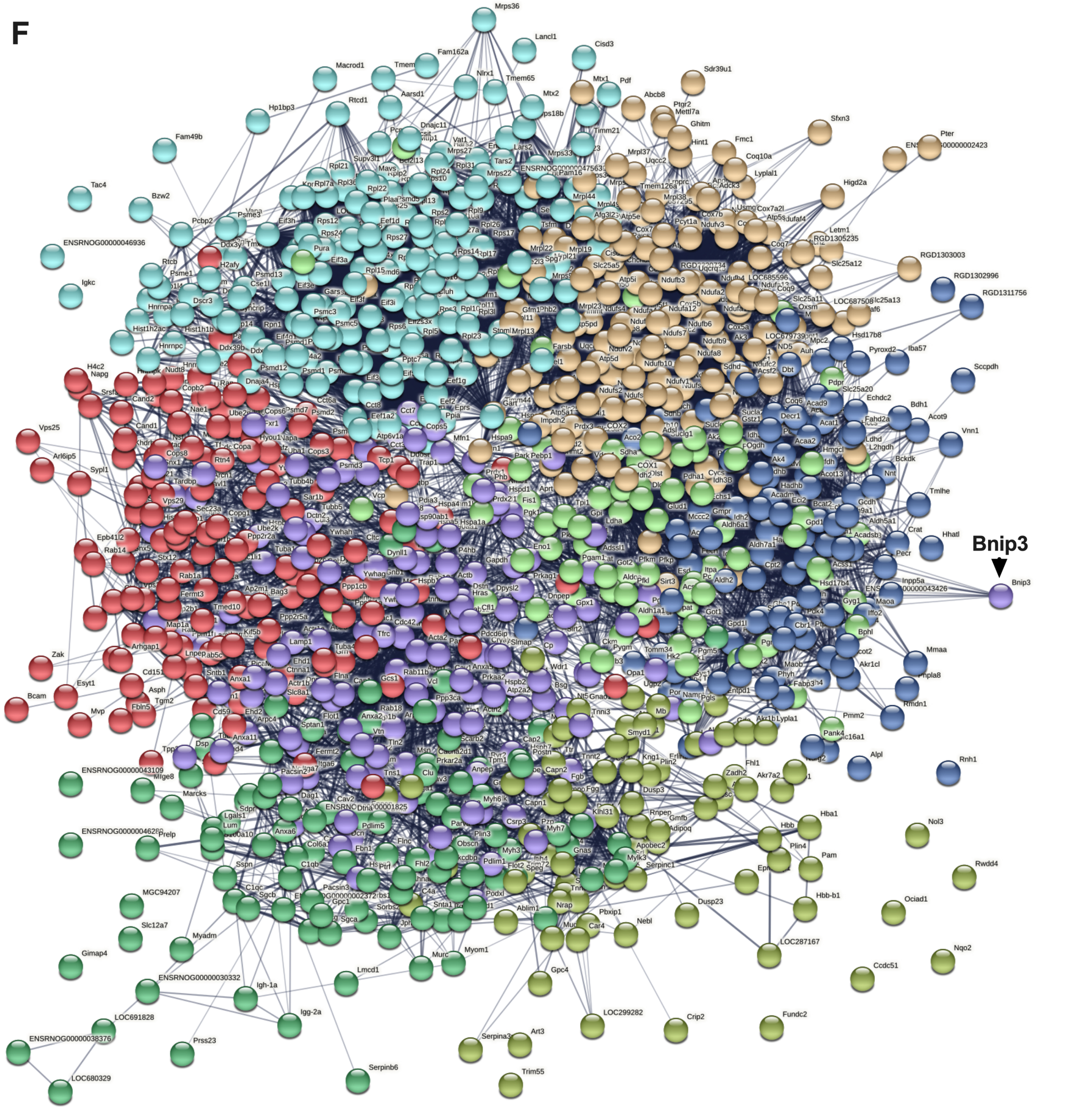

Figure S10. BNIP3 interactome in rat LV myocardium by BNIP3 Co-IP and mass spectrometry. A-C. Heat maps and PCA plots show the relative $\log_2$ fold expression and the variance in biological samples, respectively, in Sham and HFrEF rat LV myocardium of the total identified BNIP3 interacting proteins (**A**) and those that were differentially expressed taking a cutoff p-value of < 0.05 (**B**) and < 0.1 (**C**). **D-E.** Volcano plots show the Log_2 fold change of group means for the identified BNIP3 interacting proteins whose relative expression increased (red) or decreased (green) in HFrEF vs Sham rat samples taking a cutoff $-\text{Log}_{10}$ p-value of 1.3 (P < 0.05) (**D**) or 1.0 (P < 0.1) (**E**). **F.** PPI network of the total identified BNIP3 interacting proteins in rat LV myocardium, PPI enrichment p-value of 1.0e-16. Protein network was generated using STRING database. Each node represents a protein, whilst edges (lines) represent protein-protein associations based on physical and physiological interaction by confidence. The thicker the line is, the higher is the confidence. Each color represents a cluster of proteins with specific function.

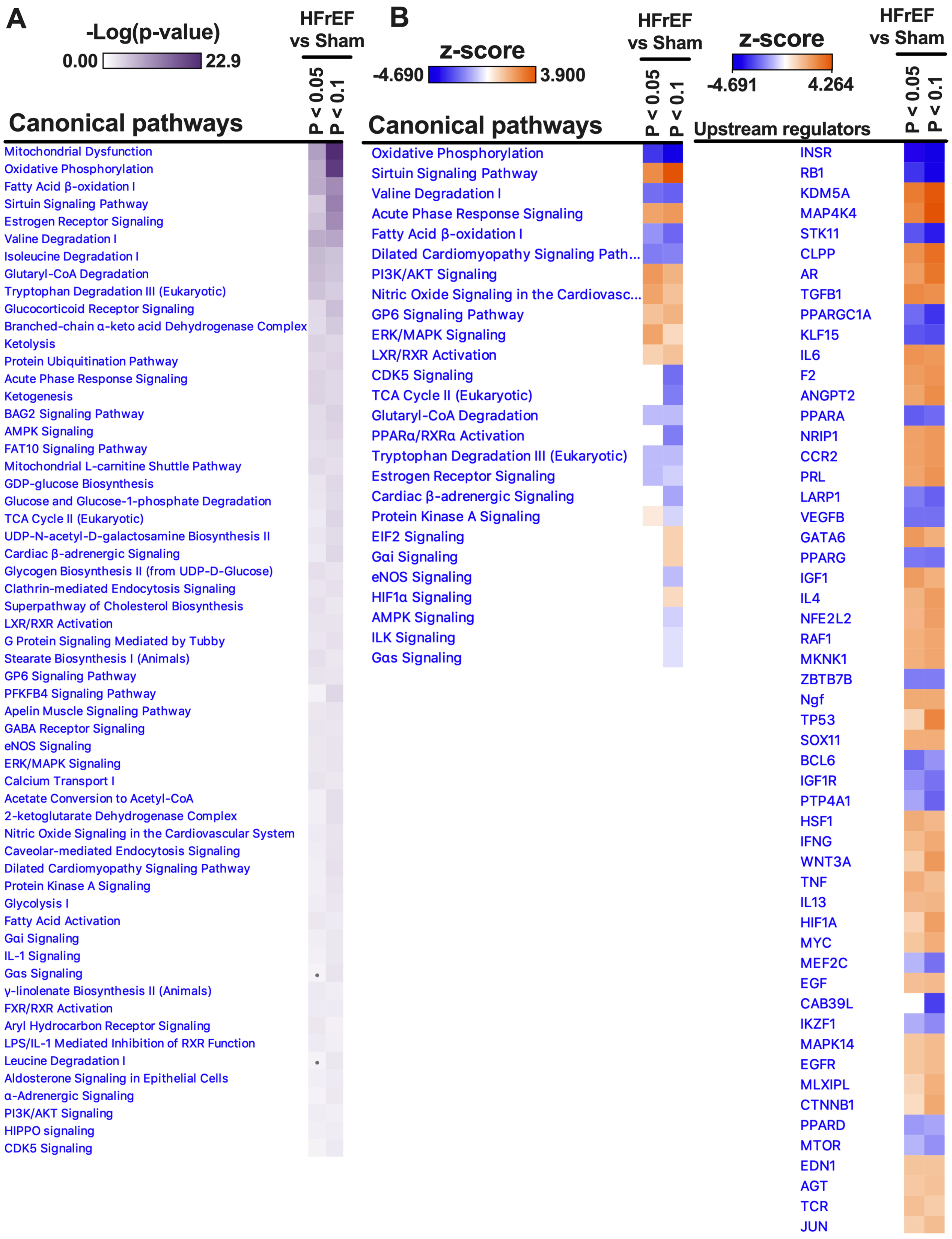
A
-Log(p-value)
0.00
22.9
HFrEF vs Sham
P < 0.05
P < 0.1
Canonical pathways
Mitochondrial Dysfunction
Oxidative Phosphorylation
Fatty Acid β-oxidation I
Sirtuin Signaling Pathway
Estrogen Receptor Signaling
Valine Degradation I
Isoleucine Degradation I
Glutaryl-CoA Degradation
Tryptophan Degradation III (Eukaryotic)
Glucocorticoid Receptor Signaling
Branched-chain α-keto acid Dehydrogenase Complex
Ketolysis
Protein Ubiquitination Pathway
Acute Phase Response Signaling
Ketogenesis
BAG2 Signaling Pathway
AMPK Signaling
FAT10 Signaling Pathway
Mitochondrial L-carnitine Shuttle Pathway
GDP-glucose Biosynthesis
Glucose and Glucose-1-phosphate Degradation
TCA Cycle II (Eukaryotic)
UDP-N-acetyl-D-galactosamine Biosynthesis II
Cardiac β-adrenergic Signaling
Glycogen Biosynthesis II (from UDP-D-Glucose)
Clathrin-mediated Endocytosis Signaling
Superpathway of Cholesterol Biosynthesis
LXR/RXR Activation
G Protein Signaling Mediated by Tubby
Stearate Biosynthesis I (Animals)
GP6 Signaling Pathway
PFKFB4 Signaling Pathway
Apelin Muscle Signaling Pathway
GABA Receptor Signaling
eNOS Signaling
ERK/MAPK Signaling
Calcium Transport I
Acetate Conversion to Acetyl-CoA
2-ketoglutarate Dehydrogenase Complex
Nitric Oxide Signaling in the Cardiovascular System
Caveolar-mediated Endocytosis Signaling
Dilated Cardiomyopathy Signaling Pathway
Protein Kinase A Signaling
Glycolysis I
Fatty Acid Activation
Gαi Signaling
IL-1 Signaling
Gαs Signaling
γ-linolenate Biosynthesis II (Animals)
FXR/RXR Activation
Aryl Hydrocarbon Receptor Signaling
LPS/IL-1 Mediated Inhibition of RXR Function
Leucine Degradation I
Aldosterone Signaling in Epithelial Cells
α-Adrenergic Signaling
PI3K/AKT Signaling
HIPPO signaling
CDK5 Signaling
B
z-score
-4.690
3.900
HFrEF vs Sham
P < 0.05
P < 0.1
Canonical pathways
Oxidative Phosphorylation
Sirtuin Signaling Pathway
Valine Degradation I
Acute Phase Response Signaling
Fatty Acid β-oxidation I
Dilated Cardiomyopathy Signaling Path...
PI3K/AKT Signaling
Nitric Oxide Signaling in the Cardiovasc...
GP6 Signaling Pathway
ERK/MAPK Signaling
LXR/RXR Activation
CDK5 Signaling
TCA Cycle II (Eukaryotic)
Glutaryl-CoA Degradation
PPARα/RXRα Activation
Tryptophan Degradation III (Eukaryotic)
Estrogen Receptor Signaling
Cardiac β-adrenergic Signaling
Protein Kinase A Signaling
EIF2 Signaling
Gαi Signaling
eNOS Signaling
HIF1α Signaling
AMPK Signaling
ILK Signaling
Gαs Signaling
z-score
-4.691
4.264
HFrEF vs Sham
P < 0.05
P < 0.1
Upstream regulators
INSR
RB1
KDM5A
MAP4K4
STK11
CLPP
AR
TGFB1
PPARGC1A
KLF15
IL6
F2
ANGPT2
PPARA
NRIP1
CCR2
PRL
LARP1
VEGFB
GATA6
PPARG
IGF1
IL4
NFE2L2
RAF1
MKNK1
ZBTB7B
Ngf
TP53
SOX11
BCL6
IGF1R
PTP4A1
HSF1
IFNG
WNT3A
TNF
IL13
HIF1A
MYC
MEF2C
EGF
CAB39L
IKZF1
MAPK14
EGFR
MLXIPL
CTNNB1
PPARD
MTOR
EDN1
AGT
TCR
JUN

C

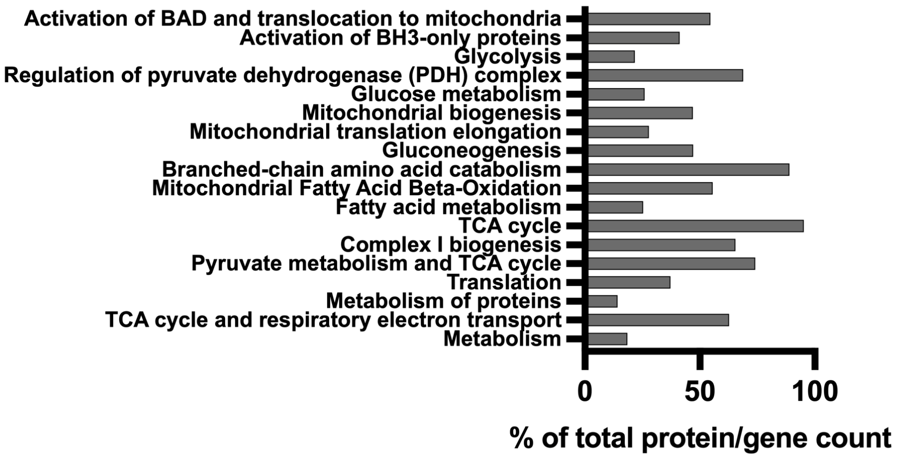

D

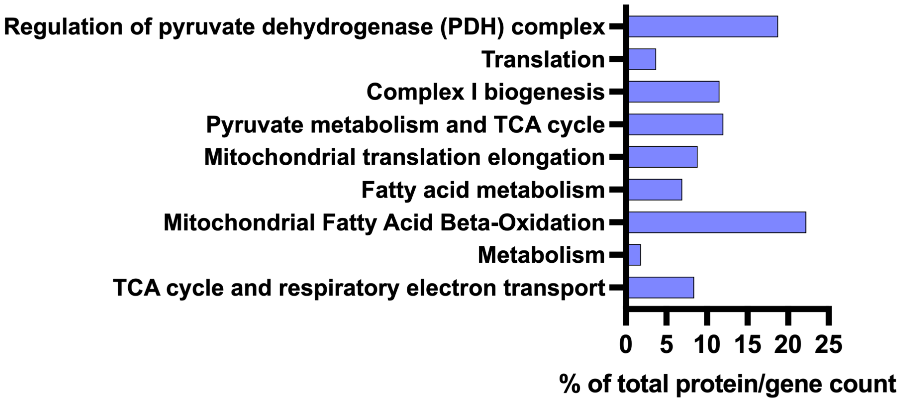

E

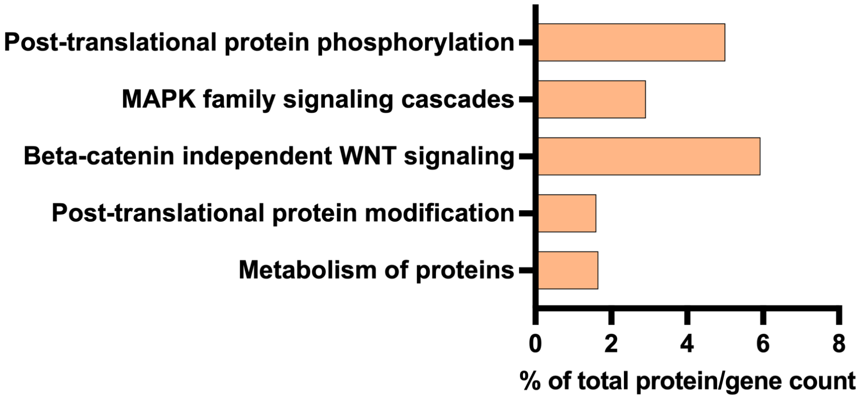

Figure S11. BNIP3 interactome enriched *Canonical Pathways*, *Upstream regulators*, and *Reactome pathways* in rat LV myocardium. A. Heat maps of the *Canonical pathways* that were enriched by -Log_{10} p-value in BNIP3 interactome HFrEF vs Sham rat LV myocardium. **B.** Heat maps of the *Canonical pathways* and *Upstream regulators* that were up-regulated/activated or down-regulated/inhibited in BNIP3 interactome HFrEF vs Sham rat LV myocardium. The orange and blue color intensities represent the z-score-based extent of up-regulation/activation or down-regulation/inhibition, respectively. In both A and B, the heat maps were generated in IPA after 'Core Analyses' of the two-group comparison (HFrEF vs Sham) taking a cutoff p-value of < 0.05 vs < 0.1, which were then compared with each other in IPA's 'Comparison Analyses' function. **C-E.** BNIP3 interactome enriched reactome pathways by q-value for the total identified BNIP3 interacting proteins in rat LV myocardium (**C**) and those that were differentially downregulated (**D**) or upregulated (**E**) in HFrEF vs Sham. Data are presented as percentage of total protein count per reactome pathway.

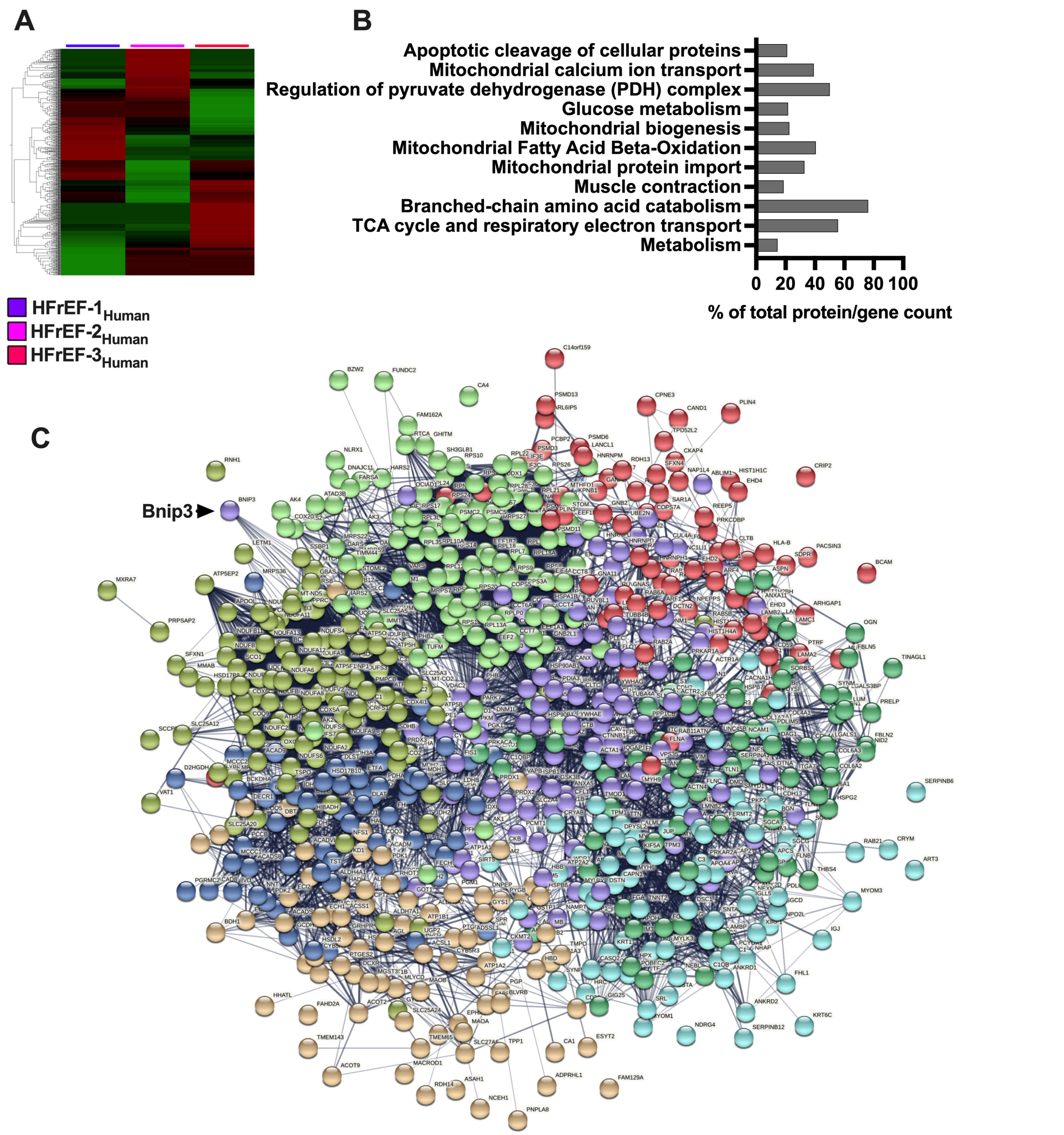

Figure S12. Visualization of BNIP3 interactome in Human HFrEF LV myocardium. A. Heat map show the relative $\log_2$ fold expression of the total identified BNIP3 interacting proteins for each of the human HFrEF LV myocardium biological sample. **B.** BNIP3 interactome enriched reactome pathways by q-value for the total identified BNIP3 interacting proteins in human HFrEF LV myocardium. Data are presented as percentage of total protein count per reactome pathway. **C.** PPI network of the total identified BNIP3 interacting proteins in human HFrEF LV myocardium, PPI enrichment p-value of 1.0e-16. Protein network was generated using STRING database. Each node represents a protein, whilst edges (lines) represent protein-protein associations based on physical and physiological interaction by confidence. The thicker the line is, the higher is the confidence. Each color represents a cluster of proteins with specific function.

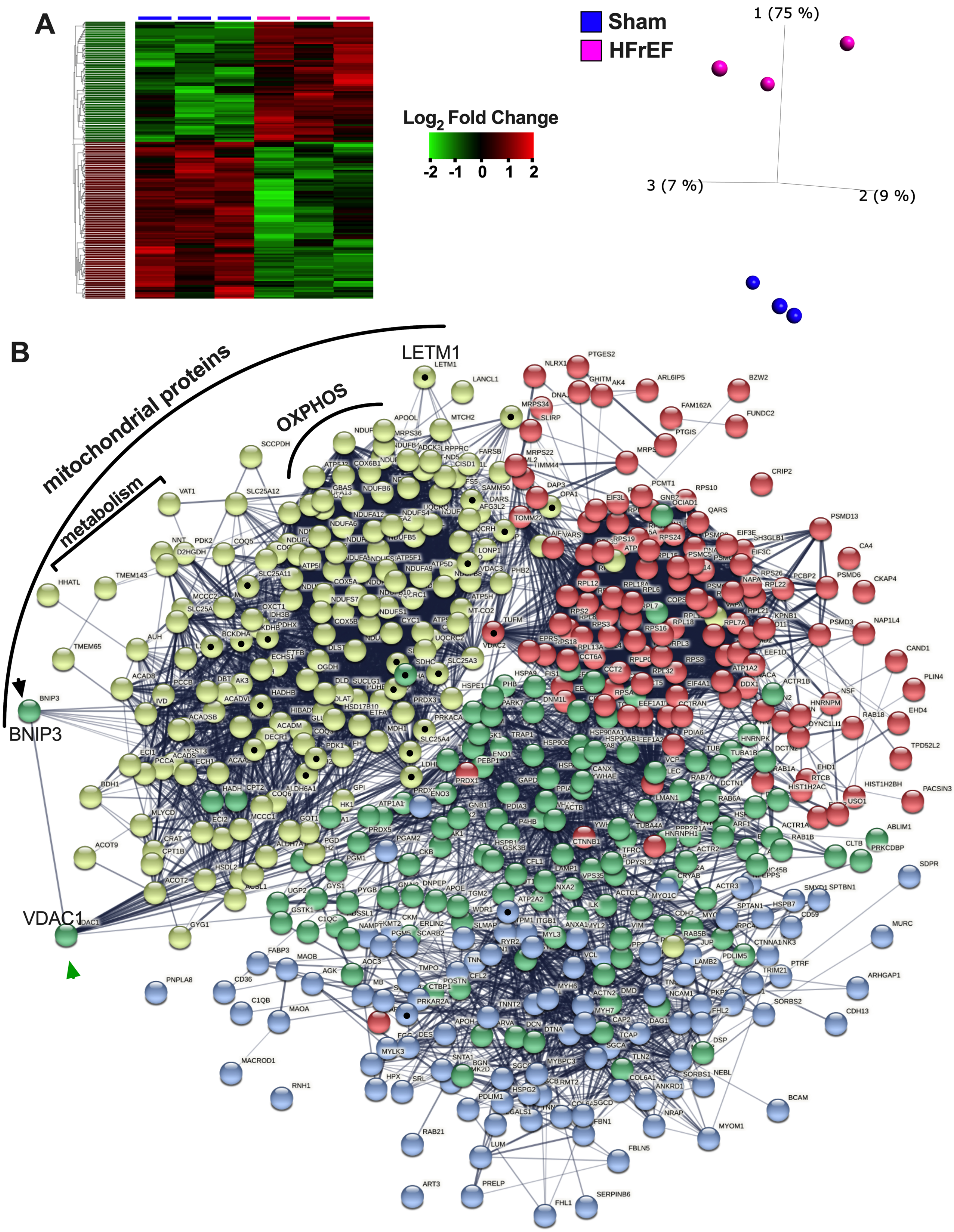

Figure S13. Visualization of the commonly identified BNIP3 interacting proteins in human and rat LV myocardium. A. Heat maps and PCA plots show the relative $\log_2$ fold expression and the variance in

biological samples, respectively, of the commonly identified BNIP3 interacting proteins that were differentially expressed in HFrEF vs Sham with a cutoff p-value of < 0.1. **B.** PPI network of the commonly identified BNIP3 interacting proteins in rat and human HFrEF LV myocardium, PPI enrichment p-value of 1.0e-16. Protein network was generated using STRING database. Each node represents a protein, whilst edges (lines) represent protein-protein associations based on physical and physiological interaction by confidence. The thicker the line is, the higher is the confidence. Each color represents a cluster of proteins with specific function. Highest interaction was between BNIP3 and VDAC1. Black and green arrowheads point at BNIP3 and VDAC1 nodes, respectively.

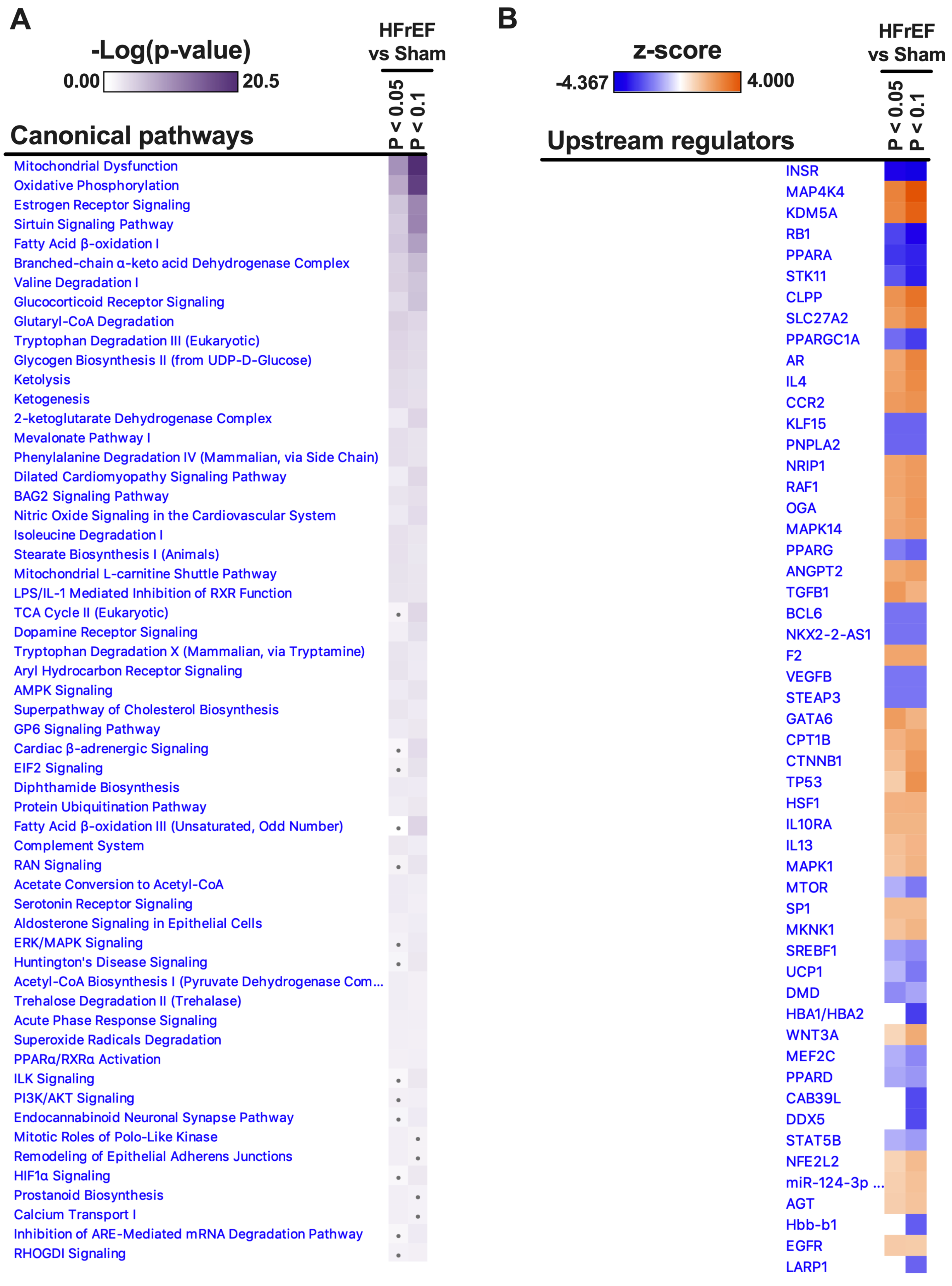
A
-Log(p-value)
0.00
20.5
HFrEF vs Sham
P < 0.05
P < 0.1
Canonical pathways
Mitochondrial Dysfunction
Oxidative Phosphorylation
Estrogen Receptor Signaling
Sirtuin Signaling Pathway
Fatty Acid β-oxidation I
Branched-chain α-keto acid Dehydrogenase Complex
Valine Degradation I
Glucocorticoid Receptor Signaling
Glutaryl-CoA Degradation
Tryptophan Degradation III (Eukaryotic)
Glycogen Biosynthesis II (from UDP-D-Glucose)
Ketolysis
Ketogenesis
2-ketoglutarate Dehydrogenase Complex
Mevalonate Pathway I
Phenylalanine Degradation IV (Mammalian, via Side Chain)
Dilated Cardiomyopathy Signaling Pathway
BAG2 Signaling Pathway
Nitric Oxide Signaling in the Cardiovascular System
Isoleucine Degradation I
Stearate Biosynthesis I (Animals)
Mitochondrial L-carnitine Shuttle Pathway
LPS/IL-1 Mediated Inhibition of RXR Function
TCA Cycle II (Eukaryotic)
Dopamine Receptor Signaling
Tryptophan Degradation X (Mammalian, via Tryptamine)
Aryl Hydrocarbon Receptor Signaling
AMPK Signaling
Superpathway of Cholesterol Biosynthesis
GP6 Signaling Pathway
Cardiac β-adrenergic Signaling
EIF2 Signaling
Diphthamide Biosynthesis
Protein Ubiquitination Pathway
Fatty Acid β-oxidation III (Unsaturated, Odd Number)
Complement System
RAN Signaling
Acetate Conversion to Acetyl-CoA
Serotonin Receptor Signaling
Aldosterone Signaling in Epithelial Cells
ERK/MAPK Signaling
Huntington's Disease Signaling
Acetyl-CoA Biosynthesis I (Pyruvate Dehydrogenase Com...
Trehalose Degradation II (Trehalase)
Acute Phase Response Signaling
Superoxide Radicals Degradation
PPARα/RXRα Activation
ILK Signaling
PI3K/AKT Signaling
Endocannabinoid Neuronal Synapse Pathway
Mitotic Roles of Polo-Like Kinase
Remodeling of Epithelial Adherens Junctions
HIF1α Signaling
Prostanoid Biosynthesis
Calcium Transport I
Inhibition of ARE-Mediated mRNA Degradation Pathway
RHOGDI Signaling
B
z-score
-4.367
4.000
HFrEF vs Sham
P < 0.05
P < 0.1
Upstream regulators
INSR
MAP4K4
KDM5A
RB1
PPARA
STK11
CLPP
SLC27A2
PPARGC1A
AR
IL4
CCR2
KLF15
PNPLA2
NRIP1
RAF1
OGA
MAPK14
PPARG
ANGPT2
TGFB1
BCL6
NKX2-2-AS1
F2
VEGFB
STEAP3
GATA6
CPT1B
CTNNB1
TP53
HSF1
IL10RA
IL13
MAPK1
MTOR
SP1
MKNK1
SREBF1
UCP1
DMD
HBA1/HBA2
WNT3A
MEF2C
PPARD
CAB39L
DDX5
STAT5B
NFE2L2
miR-124-3p ...
AGT
Hbb-b1
EGFR
LARP1

C

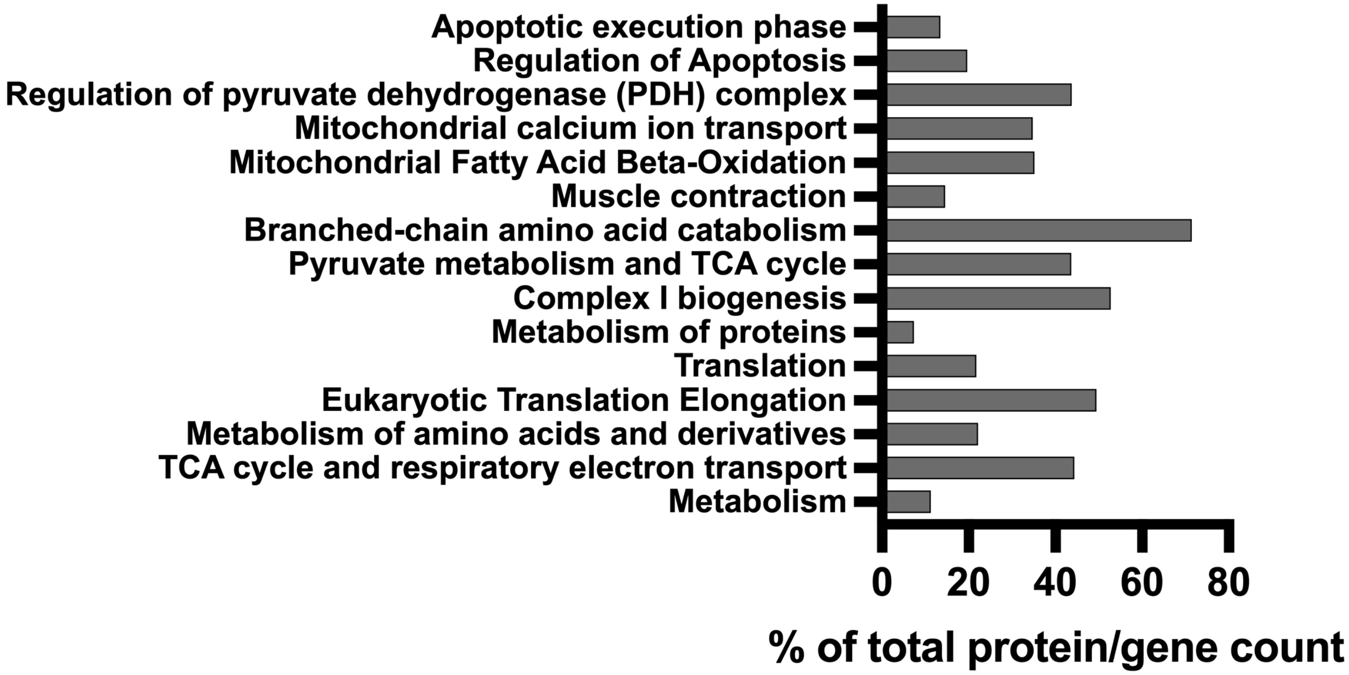

Figure S14. Enriched canonical pathways and Upstream regulators of the commonly identified BNIP3 interacting proteins in rat and human LV myocardium. Heat maps of the *Canonical pathways* that were enriched by -Log_{10} p-value (**A**) and *Upstream regulators* (**B**) that were up-regulated/activated or down-regulated/inhibited in HFrEF vs Sham of the commonly identified BNIP3 interacting proteins between rat and human LV myocardium. The orange and blue color intensities represent the z-score-based extent of up-regulation/activation or down-regulation/inhibition, respectively. Heat maps were generated in IPA after ‘Core Analyses’ of the two-group comparison (HFrEF vs Sham) taking a cutoff p-value of < 0.05 vs < 0.1, which were then compared with each other in IPA’s ‘Comparison Analyses’ function. **C.** BNIP3 interactome enriched reactome pathways by q-value for the commonly identified BNIP3 interacting proteins between human and rat LV myocardium. Data are presented as percentage of total protein count per reactome pathway.
